# Prognostic Significance of *WWOX*/*HIF1A* Ratio in Cancer Subtypes: Insights into Metabolism, ECM, and EMT

**DOI:** 10.3390/biology14091151

**Published:** 2025-09-01

**Authors:** Izabela Baryła, Raneem Y. Hammouz, Kinga Maciejek, Andrzej K. Bednarek

**Affiliations:** Department of Molecular Carcinogenesis, Medical University of Lodz, Zeligowskiego 7/9, 90-752 Lodz, Poland; izabela.baryla@umed.lodz.pl (I.B.); raneem.hamouz@umed.lodz.pl (R.Y.H.); kinga.maciejek@student.umed.lodz.pl (K.M.)

**Keywords:** WWOX, HIF1A, EMT, ECM, metabolism, pathways, tumour aggressiveness, patient outcomes

## Abstract

WWOX and HIF1α proteins are involved in cancer progression; their functions are closely related. WWOX binds HIF1α through its WW domains, sequestering it in the cytoplasm and inhibiting its transcriptional activity. This study evaluates the prognostic significance of the *WWOX*/*HIF1A* interaction across cancers, breast cancer subtypes, glioblastoma (GBM), low-grade glioma (LGG), and hepatocellular carcinoma (HCC) through gene expression and pathway analysis focused on metabolism, ECM, and epithelial–mesenchymal transition. In breast cancer, metabolic pathways correlated with good prognosis in basal subtypes. HER2 subtypes showed enrichment in DNA replication pathways. Luminal A subtypes showed favourable prognosis via TNF and PI3K/AKT signalling, while luminal B subtypes had poor prognosis tied to metabolic activity; genes associated with good prognosis mirrored those tied to poor prognosis in luminal A. In HCC, enhanced metabolic activity was associated with good prognosis. In contrast, poor prognosis involved TNF signalling and cytoskeleton-related pathways, indicating more aggressive tumour behaviour. In LGG, good prognosis was linked to metabolic and cAMP pathways, while poor outcomes involved TNF, cell cycle, apoptosis, and focal adhesion pathways. GBM showed similar patterns: metabolic and cAMP pathways indicated better outcomes, while NFKB, TNF, JAK-STAT, and PI3K/AKT pathways marked poor prognosis. These findings suggest the *WWOX*/*HIF1A* ratio is a robust prognostic marker and a possible guide for developing targeted treatments.

## Simple Summary

*WWOX* is a gene that plays a key role in regulating cellular processes related to maintaining homeostasis and protecting against cancer, through interactions with many proteins and signalling pathways. One of the important partners of WWOX is the hypoxia inducible factor HIF1α. Through this interaction, WWOX modulates cancer cell metabolism, which serves as an important prognostic factor. This study assessed the prognostic significance of the *WWOX*/*HIF1A* ratio in various cancers: breast cancer subtypes, glioblastoma multiforme, low-grade glioma, and hepatocellular carcinoma. It was found that in breast cancer, the *WWOX*/*HIF1A* ratio allows for the identification of groups of patients with favourable and unfavourable prognosis—in basal and HER2 breast cancer subtypes, high *WWOX*/*HIF1A* was associated with a more favourable prognosis, whereas in luminal A and B subtypes, it correlated with a less favourable prognosis. In the remaining analysed cancers, a higher *WWOX*/*HIF1A* expression ratio also allowed for determining the prognosis; in brain tumours and hepatocellular carcinoma, it clearly correlated with better survival. In conclusion, the *WWOX*/*HIF1A* expression ratio might be considered as a potential biomarker determining the prognosis of cancer patients.

## 1. Introduction

Cancer, a complex and multifaceted disease characterized by genetic alterations, disruption of normal cellular processes, uncontrolled cell growth, and proliferation [1,2]. A key feature of cancer cells is also their ability to adapt to diverse and often hostile microenvironments [3,4,5]. This malignant growth and easy adaptation often involve the reprogramming of cellular metabolism [3]. Cancer cells often prefer glycolysis as their main energy source, even when oxygen is readily available—a phenomenon known as the Warburg effect [6,7,8]. This seemingly paradoxical glycolysis preference despite the oxygen availability, which produces less ATP per unit of glucose compared to oxidative phosphorylation (OXPHOS), is a hallmark of cancer metabolism [9]. To promote this metabolic shift, cancer cells upregulate HIF1α, a key regulator of cellular adaptation to hypoxia. HIF1α enhances glycolysis by upregulating glucose transporters, such as GLUT1, and glycolytic enzymes like hexokinase, pyruvate kinase, and lactate dehydrogenase, to increase glucose flux through glycolysis to lactate. Simultaneously, HIF1α regulates the expression of pyruvate dehydrogenase kinase 1 (*PDK1*), inhibiting mitochondrial respiration and further enhancing glycolysis [6]. The excessive activation of HIF1α in cancer, despite oxygen availability, is often driven by oncogenes (e.g., ERBB2, PI3K, Ras, protein kinase B/AKT, mTOR) and mutations in tumour suppressors (e.g., Von Hippel–Lindau (VHL) and PTEN) [6,10]. This leads to increased transcription of glycolytic genes by HIF1α and subsequent lactate production.

It is important to note that aerobic glycolysis is not uniform across all tumour types or even within a single tumour microenvironment, reflecting the complex interplay of factors influencing cancer metabolism [6]. One of these factors is the physical interaction between the WW domain of WW domain-containing oxidoreductase WWOX and HIF1α [11]. WWOX is recognized as a crucial tumour suppressor, not only inhibiting tumour formation but also regulating essential pathways involved in cancer progression [12,13,14]. WWOX is implicated in the regulation of glycolysis, fatty acid degradation, and other pathways that generate acetyl-CoA, a key molecule in energy production, highlighting its role in metabolism and cellular homeostasis [15,16]. Since the HIF1α transcription factor is a main factor for cancer metabolic adaptation, WWOX regulation of HIF1 is particularly relevant. WWOX modulates HIF1α protein levels, diminishes its transactivation function, and coordinates glucose metabolism and the Warburg effect [11,17]. WWOX deficiency is associated with enhanced glycolysis and decreased mitochondrial respiration, making cells more tumourigenic. The knockout of WWOX in various cell lines leads to increased expression of the HIF1α protein and its accumulation in the nucleus, where it can exert its transcriptional effects [11,17]. *WWOX* KO inhibits HIF1α hydroxylation, a process that normally marks HIF1α for proteasomal degradation. Conversely, WWOX overexpression suppresses HIF1 activity, confirming its role in modulating HIF1α transactivation function through direct physical interaction.

While the precise mechanism by which WWOX affects HIF1α remains under investigation, it has been proposed that WWOX can interact with HIF1α and inhibit its transactivation function, possibly by sequestering HIF1α in the cytoplasm, or WWOX is necessary for HIF1α to be labelled for degradation in the proteasome under normoxic conditions [11]. Most importantly, disrupting the WWOX-HIF1α interaction results in increased expression of *HIF1* target genes, including glucose transporters, glycolysis enzymes, and Krebs cycle inhibitors. These molecular changes are accompanied by metabolic shifts indicative of increased glycolysis, such as increased glucose uptake, enhanced enzymatic activity of hexokinase and lactate dehydrogenase, and elevated lactate production [11,17]. On the other hand, low oxygen consumption, ATP production, and reduced levels of the TCA cycle intermediates were also observed. These findings highlight the critical role of WWOX in regulating cellular metabolism [11]. However, WWOX’s function as a tumour suppressor gene is much more complex. The HIF1α protein is not WWOX’s only partner. Its binding with other proteins, including several transcription factors, means these interactions trigger diverse effects within the cell.

The main purpose of the Warburg effect is to provide the energy necessary for uncontrolled cell growth and proliferation [2]. It confers several advantages to tumour cells, including rapid energy provision via ATP biosynthesis, increased availability of biosynthetic intermediates, and diminished production of reactive oxygen species (ROS), thereby contributing to cancer cell protection from apoptosis [18]. This process leads to the generation of metabolites that are implicated in different oncogenic pathways and facilitates the uptake and incorporation of nutrients into the biomass needed to generate new cells, enhancing proliferation [19,20]. This translates into the observation that gene expression profiles related to the Warburg effect hold promise for prognostic purposes, but more importantly, to better explain the complex and diverse metabolism of cancer cells. Glycolytic phenotypes are generally linked to more advanced disease stages with poorer survival rates in cancer patients [21].

The benefits of the Warburg effect on cancer also result from its influence on the alteration of the tumour microenvironment (TME). It is known to actively promote cancer progression and poor tumour immunosurveillance, which is a significant issue in the context of treatment response [20]. Excess lactate produced by overly intense glycolysis is responsible for acidification of the TME. Higher lactate levels are associated with a less favourable prognosis in cancer patients, and metastatic tumours exhibit higher lactate levels in the TME compared to non-metastatic tumours [20,22].

The Warburg effect has long been known as a key mechanism for controlling metabolism in cancer cells, but only recently has its role been emphasized in activating immune cells (including macrophages, T cells, and NK cells) that modulate the TME and influence the course of cancer. Lactate modulates immune cell functions, reshaping T cells and macrophages into immunosuppressive phenotypes such as tumour-promoting regulatory T cells (Tregs) and M2-like tumour-associated macrophages (TAMs) [23]. This immunosuppressive shift supports tumour growth and maintenance. Additionally, lactate-induced acidification of the TME inhibits CD8+ T-cell functionality via p38/JNK pathway suppression and reduces the anti-tumoural activity of natural killer (NK) cells through mTOR pathway inhibition [20]. What is more, lactate plays a critical role in tumour growth and metastasis by promoting angiogenesis through the activation of pathways such as NF-κB, PI3K-AKT-CREB, and HIFs, which upregulate pro-angiogenic factors like amphiregulin (AREG), IL-8, and VEGF. These factors stimulate new blood vessel formation, which is essential for tumour survival and progression [22,24,25,26,27]. Lactate’s signalling properties within the TME further enhance its capacity to drive cancer development and spread.

All these data confirm that the Warburg effect is not only a metabolic phenomenon source but also a mechanism that modulates cancer signalling and organizes the TME. WWOX, by tightly regulating HIF1α, is responsible for this mechanism in cancer cells consequently associated with cancer cell proliferation, invasiveness, patients’ survival, and treatment response. WWOX and HIF1α were studied separately and extensively in many cancers. However, to date, there is a paucity of data available examining the effect of WWOX/HIF1α signalling pathway on patient survival in breast cancer, hepatocellular, and glioblastoma. Our study aimed to investigate whether the *WWOX*/*HIF1A* ratio affects the overall survival of patients, whether it allows for the identification of differences between cancer subtypes, and through which pathways it exerts its regulatory effects.

### 1.1. WWOX/HIF1A and Breast Cancer

Breast cancer, the most frequently diagnosed cancer worldwide and the leading cause of cancer deaths in women in Europe, is characterized by genetic and clinical heterogeneity and a hypoxic microenvironment resulting from rapid cell proliferation driven by the Warburg effect, which promotes aggressive disease progression, metastasis, and treatment resistance [28,29,30,31].

HIF1α plays a crucial role in adapting to this hypoxic stress. It is overexpressed and activated in breast cancer, particularly in precursor lesions, early-stage disease, and the aggressive triple-negative breast cancer (TNBC) subtype [30]. It drives the Warburg effect, which is perpetuated through mutations and epigenetic changes, promoting proliferation, metastasis, and drug resistance [32]. In contrast, *WWOX* expression is significantly reduced or absent in 96.6% of TNBC patients, which correlates with increased EMT, metastasis, chemotherapy resistance, and shortened survival [12,33,34,35,36,37,38]. Lower levels of WWOX in lymph node metastases than in primary tumours support its role as a marker of breast cancer aggressiveness [12]. This inverse relationship between WWOX and HIF1α highlights the key link between loss of WWOX and enhanced Warburg effect in breast cancer.

WWOX tumour suppressor function was studied in several breast cancer cell lines. However, research findings concerning both *WWOX* and *HIF1A* are very limited. Downregulation of *WWOX* in the MCF7 cell line (derived from the luminal A subtype) resulted in upregulation of key glycolytic genes via activation of HIF1α, such as *GLUT1*, *HK2*, *PKM2*, and *PDK1*. At the same time, restoration of *WWOX* has the opposite effect [11]. Furthermore, in human breast cancer samples, WWOX expression was inversely correlated with the level of the glucose transporter GLUT1, a direct target of HIF1α. Furthermore, studies in *wwox*-deficient mouse models showed increased tumour formation, an effect abolished by HIF1α depletion, highlighting the complex interaction between these two factors [11]. Data support that WWOX inhibits excessive HIF1α transactivation, acting as a protective factor, a mechanism that is impaired in breast cancer [11]. This disruption of WWOX-mediated metabolic control contributes to the Warburg effect and its downstream consequences. Altered glucose metabolism, essential for cellular energy supply [39], leads to increased glycolysis and uncontrolled proliferation of breast cancer cells. The byproduct of glycolysis, lactic acid, accumulates in the TME, resulting in a more acidic pH that further promotes tumour progression by affecting angiogenesis, immunosuppression, proliferation, and survival [40].

Not only are *HIF1A* and *WWOX* differentially expressed between malignant and benign breast cancer tissue, but this is also true for the expression of key glycolysis genes. *GLUT1*, *HK2*, *PFK1*, and *GAPDH* are significantly overexpressed in malignant breast cancer tissue [32,41]. Furthermore, PFK1 and GAPDH show even higher protein expression levels in malignant breast cancer tissues from obese women. Furthermore, breast cancer reprograms energy metabolism in tumour-associated adipose tissue, increasing HK2 expression in malignant tumours compared to benign tumours [32].

TNBC does not respond to hormonal therapy or anti-HER2 drugs due to the lack of appropriate receptors, and although targeted therapies and immunotherapy may partially limit its aggressiveness, the clinical benefit is small. Therefore, a promising treatment strategy for refractory TNBC seems to be to target the Warburg effect, as confirmed by numerous in vitro studies. Single inhibitors of glycolysis often fail due to the metabolic flexibility of cancer cells; therefore, future therapies should target HIF1α (e.g., via WWOX), which may more effectively inhibit aberrant glycolysis [32].

### 1.2. WWOX/HIF1A and Hepatocellular Carcinoma

Another cancer in which the perturbation of WWOX-HIF1α interaction and its impact on carcinogenesis has been studied is hepatocellular carcinoma (HCC) [42]. HCC is the predominant histological subtype of liver cancer, representing approximately 90% of all primary liver cancer cases, and it ranks as the third leading cause of cancer-related deaths globally [43]. The major risk factors for the development of HCC are chronic HBV/HCV infections, alcohol abuse, and exposure to xenobiotics such as aflatoxins. The increasing incidence of non-alcoholic fatty liver disease (NAFLD) is also contributing to HCC cases. Most HCC cases arise in the context of cirrhosis, with key pathogenic mechanisms including chronic inflammation, DNA damage, oxidative stress, and TME interactions. These factors underscore the complexity of HCC etiopathogenesis and highlight the need for personalized therapeutic approaches [44,45]. HCC is a hypermetabolic tumour that consumes more oxygen than the surrounding normal tissues [46]; however, only about 50–70% of HCC cases exhibit hypermetabolic activity using PET imaging [47]. Over-proliferating HCC cells consume oxygen, which, together with the lack of adequate tumour vascularization, leads to hypoxic conditions [46]. The HIF1α expression in HCC tissues is higher than in corresponding adjacent tissues, and HCC patients with higher HIF1α expression have poorer prognosis. It has been established a relationship between HIF1α and tumour cell proliferation, invasion, metastasis, recurrence, and vascular proliferation of HCC [46,48].

Like in breast cancer, HIF1 activates in HCC cells key enzymes, which are involved in glucose metabolism and glycolysis [46], like GLUT1 [48], HK2, ENO1, GAPDH, PFKL, PGK1, PFKFB3, LDHA [46], and PDK1. Consequently, an excess of glycolysis but reduction of oxidative phosphorylation and oxygen consumption by the mitochondria is observed in HCC [46]. In HCC, excessive glycolysis results in increased lactate production, which is exported from the cell by MCT transporters, leading to a decrease in intracellular pH [46]. This process also acidifies the TME and promotes angiogenesis, metastasis, drug resistance, and immunosuppression [48].

HCC patients exhibit chromosomal regions of copy number loss, with one being the WWOX locus [42]. HCC tissues are decreased or absent in WWOX expression compared to their matched normal tissues [42,49]. Moreover, HCC patients with reduced WWOX mRNA expression have worse survival outcomes than patients with higher [49]. The low expression of WWOX correlates with poor tumour differentiation, the present microvascular invasion, and advanced stage, which points to an aggressive tumour phenotype [42]. What is more interesting, lower WWOX expression is also observed in cancer-adjacent liver tissue. Probably, this allows us to assume that WWOX loss can be an early event in liver carcinogenesis [42].

WWOX mRNA and protein expression are also lower in many HCC cell lines than in normal liver cell lines [49]. The WwoxΔHep mouse model confirms that loss of WWOX accelerates HCC development following exposure to carcinogens. Even tumours in mice exposed to a carcinogen, without manipulation of WWOX expression, showed reduced WWOX expression compared to healthy tissue, and gene inactivation of WWOX promoted cell proliferation and tumour progression [42]. WWOX loss is associated with enhanced survival of a hepatoma cell line, whereas WWOX overexpression induces apoptosis and suppresses proliferation. HCC cell proliferation, migration, and invasion are associated with overexpression of Warburg effect genes [43], which increase aerobic glycolysis and lactate production, promoting angiogenesis, migration, and immunosuppression in HCC [48]. In the WwoxΔHep mouse model, both carcinogen-treated and untreated mice exhibited nuclear HIF1α localization along with upregulated glycolytic HIF1α-target genes compared to controls, highlighting *WWOX* loss as a driver of metabolic dysregulation in HCC. These metabolic changes were observed already at the pre-tumour stage, suggesting that they play a crucial role in promoting HCC development. The findings collectively demonstrate that WWOX loss initiates early metabolic reprogramming essential for HCC development, as its absence impairs suppression of tumourigenic pathways during initial disease stages [42].

### 1.3. WWOX/HIF1A and Brain Tumours

Glioblastoma (GBM) is the most aggressive and highly malignant brain primary tumour with an inferior prognosis and a median overall survival of only 14.6–20.9 months [19,21,50]. The poor survival is due to the highly invasive, chemoresistant, and recurrent nature of GBM. One of the reasons, among others, is the ability of GBM cancer cells to undergo the metabolic shift Warburg effect. The Warburg phenotypes were enriched in the mesenchymal GBM cells subgroup, whereas functional mitochondrial metabolism predominated in healthy tissues [21].

One of the indicators of poor prognosis in GBM patients is the overexpression of HIF1α. It correlated with shorter OS [51,52] but is not linked to progression-free survival (PFS) [52]. The HIF1α expression increases gradually with the increase of the grade of glioma [53] and in GBM patients whose tumours reoccurred after treatment [51]. *HIF1A* mRNA levels are higher in IDH wild-type GBM than in those with an IDH mutation [51], confirming the characteristic metabolic profile of IDH mutant tumours, including reduced glycolysis [51]. In glioblastoma, increased HIF1α expression, driven by altered tumour suppressors like p53 and STAT, promotes the Warburg effect. This results in increased glucose concentration in the brain, which is correlated with increased malignancy in GBMs. It promotes GBM cell proliferation, therapeutic resistance, and intracranial growth [19,54]. The role of glucose metabolism in brain cancer cell proliferation is important because the brain uses large amounts of it as its main source of energy [55,56]. What is more, GBM patients harbour a mutation in the genes directly involved in glycolysis as *HK2* and *GPI*, *LDHA*, *LDHB*, and *LDHD*, in addition to mutations in genes responsible for metabolic regulation, such as *TP53*, *HIF1A*, *STAT3*, *MTOR* [50], which most likely influence the GBM prognosis. Beyond glycolysis, Warburg effect genes regulate non-glycolytic processes; e.g., PKM2 enhances GBM cell survival by phosphorylating Bcl-2 to inhibit apoptosis and glioma malignancy [57].

Lactic acidosis is common in GBM and interrupts a cascade of biochemical reactions that alter metabolism and signalling pathways [54,58]. This further acidifies the TME, promoting infiltration by immunosuppressive immune cells. Their activation contributes to both disease progression and poor prognosis in GBM patients [50]. Acidification of the glioma TME activates prometastatic genes such as *MMP2*, *MMP7*, *PLAU*, and suppresses antimetastatic ones (*MTSS1*, *TIMP2*, *CTSK*) [54]. It also contributes to the development of drug resistance in GBM [58]. On the other hand, silencing *LDHA* in GBM cells reduces the level of lactate and glucose utilization. This results in a significant reduction in the number of colonies, increased apoptosis, and attenuates tumour growth and invasion of GBM cells [54].

The importance of *WWOX* in modulating the Warburg effect in GBM has already been reported. The *WWOX* overexpression in the T98G cell line induces changes in the expression of about 3000 genes, most of which are involved in metabolic processes [59]. Manipulation of WWOX level led to a slowdown in cell proliferation, increased apoptosis, and enhanced mitochondrial redox potential, which directly affects the attenuation of the Warburg effect [59]. A reduced invasive potential of GBM was also observed [59]. This is particularly important given that reduced WWOX expression is observed in GBM patients. WWOX expression correlates positively with the antiapoptotic gene Bcl2 and the cell proliferation marker Ki67 in GBM samples [60].

GBM is a unique model of metabolic heterogeneity, with cells switching between aerobic glycolysis and OXPHOS, supporting survival and requiring personalized therapy [21]. Patient heterogeneity also includes a subgroup with high WWOX expression linked to a cancer-promoting profile and lack of prognostic benefit, which may be important in the future in qualification for personalized GBM therapy [61].

In the presented work we analysed TCGA data, exploring how the *WWOX*/*HIF1A* ratio is associated with differentiation of tumour transcriptomes of breast cancer, hepatocellular carcinoma, and brain tumours. We determine diverse metabolic profiles and patients’ survival, as well as specific markers in tumour metabolism associated with the differential *WWOX*/*HIF1A* ratio.

## 2. Materials and Methods

### 2.1. Data Extraction

Gene expression profile data for glioblastoma (159 samples), hepatocellular carcinoma (371 samples), low-grade glioma (515 samples), and breast cancer (505 samples) were obtained from The Cancer Genome Atlas (TCGA) database “http://gdac.broadinstitute.org/ (accessed on 28 November 2024)”. These datasets were level 3 normalized using the Illumina HiSeq RNA-seq “http://gdac.broadinstitute.org/ (accessed on 28 November 2024)” platform and included associated clinical information. Data acquisition was conducted through the TCGA Genomic Data Commons (GDC) portal “http://gdac.broadinstitute.org/ (accessed on 28 November 2024)” in compliance with TCGA data access policies. To ensure data integrity and compatibility, patients with missing clinical or gene expression values were excluded from the analysis. Extracted data encompassed gene expression levels, survival outcomes, and clinical characteristics. A summary of clinical traits for the analysed cohorts is provided in Supplementary Table S1.

### 2.2. Cutpoint Determination

Optimal cutpoints for stratifying patients based on their *WWOX*/*HIF1A* expression ratio were identified using the Evaluate Cutpoints application in R [62]. This tool integrates R packages such as survival, OptimalCutpoints, maxstat, and ggplot2 [63] to identify statistically significant cutpoints for continuous variables. Patients were divided into two groups: those with a *WWOX*/*HIF1A* ratio above the cutpoint, “Group Above”, and those below it, “Group Below”. Kaplan–Meier survival curves were generated to visualize survival differences between these groups.

### 2.3. Multivariate Analysis

Multivariate Factor Analysis (MFA) was performed to explore relationships among gene expression profiles and patient subgroups. The analysis utilized the R packages FactoMineR [64] and factoextra [65]. Row clustering was based on Pearson’s distance metric with complete agglomeration methods, enabling identification of distinct patient subgroups. Unsupervised hierarchical clustering was conducted using Pearson correlation and pairwise complete-linkage methods via the gplots [66] package to explore interaction patterns based on median expression values. To assess the independent prognostic impact of key genes, multivariate Cox proportional hazards regression was performed (Supplementary Figures S1–S7). The models included the *WWOX*/*HIF1A* ratio, age at diagnosis, and the expression levels of genes listed in Table 1, which were identified as contributing to prognosis according to the *WWOX*/*HIF1A* ratio for each cancer subtype. All gene expression variables and the ratio were treated as continuous predictors and, when required, were log2-transformed and standardized (z-score) prior to analysis. Age was included as a continuous variable.

Cox regression models were fit using the *coxph* function from the R package survival (version 3.8-3) [67]. Hazard ratios (*HR*), 95% confidence intervals (CI), and Wald test *p*-values were reported for each covariate. Forest plots were generated with ggplot2 [68] to visualize the results. Model discrimination was assessed using the concordance index (*C*-*index*).

The proportional hazards assumption was tested for all variables using Schoenfeld residuals (cox.zph); no significant violations were observed. For each variable, HR  >  1 indicated increased risk, and HR  <  1 indicated a protective effect. Statistical significance was considered at *p*  <  0.05. For variables with extreme *HR* values or wide confidence intervals, results were interpreted within their biological context and evaluated alongside model fit indices (AIC, *C-index*).

### 2.4. Differential Gene Expression Analysis

Differentially expressed genes (DEGs) between stratified patient groups were identified using the EdgeR [69] package in R. Statistically significant differences in gene expression associated with *WWOX*/*HIF1A* ratio-based stratification were confirmed. Statistical significance was determined with a false discovery rate (FDR) threshold of <0.05, applying the Benjamini–Hochberg procedure to control for multiple testing.

### 2.5. Association Analysis Between DEGs and Patient Prognosis

Survival analyses demonstrated whether a higher or lower *WWOX*/*HIF1A* ratio correlated with improved patient outcomes. Pathways linked to favourable or unfavourable/poor prognoses were investigated, focusing on ECM, EMT, and Warburg-related genes (Supplementary Tables S2–S8). Functional classification of DEGs involved literature reviews, database searches, and gene ontology (GO) enrichment analysis using tools such as srplots [70]. FDR threshold of < 0.05 was applied for DEG identification in each phenotype separately. Upregulated and downregulated gene lists derived from transcriptomic data were mapped onto KEGG pathway maps using the KEGG Mapper Color tool “https://www.genome.jp/kegg/mapper/color.html (accessed on 1 November 2023)” [71] (Supplementary File S1). Differential expression patterns were visualized, with yellow indicating favourable/good prognosis and pink indicating unfavourable/poor prognosis.

### 2.6. Reproducibility and Data Availability

All analyses were conducted in R (version 4.5.0), and the corresponding scripts, Supplementary Materials will be made publicly available to ensure transparency and reproducibility. Data handling and sharing comply with TCGA data access policies.

## 3. Results

### 3.1. Breast Cancer-Basal Subtype

**Figure 1:**
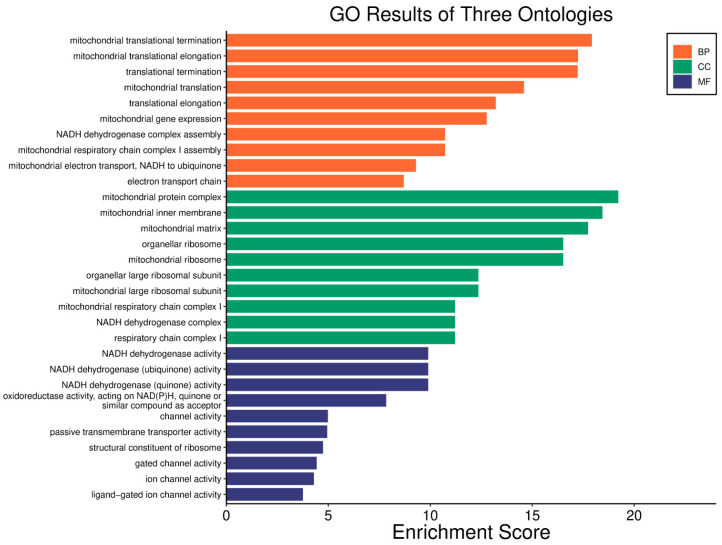
Basal subtype with high *WWOX*/*HIF1A* ratio: good prognosis ontology. Patients exhibiting a higher *WWOX*/*HIF1A* ratio showed significantly improved survival rates. This favourable prognosis was associated with metabolic shifts favouring oxidative phosphorylation over glycolysis, which limits tumour proliferation and supports tumour suppression. BP—Biological process, CC—Cellular Component, MF—Molecular Function.

**Figure 2:**
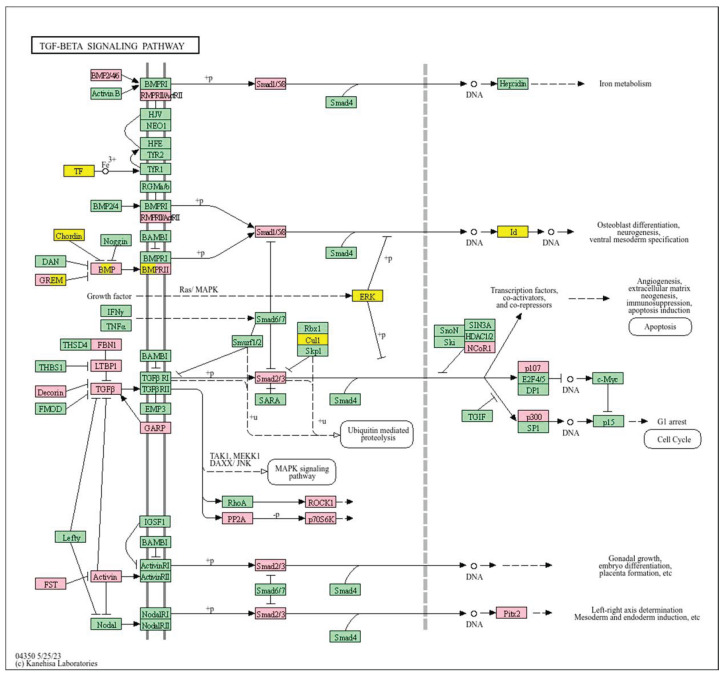
TGF-β signalling pathway in basal subtype with prognostic implications. The TGF-β signalling pathway is differentially expressed in the basal subtype and stratifies prognosis according to the *WWOX*/*HIF1A* ratio. Good prognosis tumours show higher expression of genes involved in oxidative metabolism and tumour suppression. In the figure, yellow indicates good prognosis, pink indicates poor prognosis, and green indicates genes with unchanged expression.

**Figure 3:**
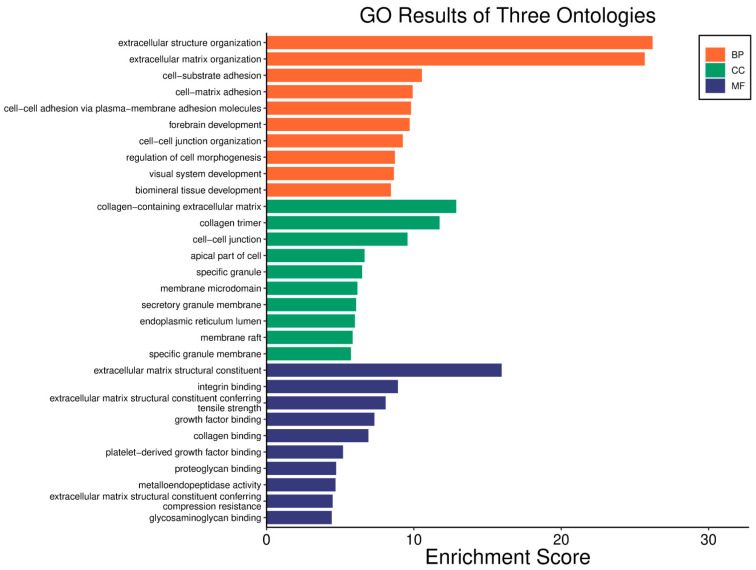
Basal subtype with low *WWOX*/*HIF1A* ratio: poor prognosis ontology. Patients with a lower *WWOX*/*HIF1A* ratio demonstrated poorer survival outcomes. This adverse prognosis correlated with increased cell adhesion and extracellular matrix (ECM) remodelling, which enhances tumour invasiveness and aggressiveness. BP—Biological process, CC—Cellular Component, MF—Molecular Function.

**Figure 4:**
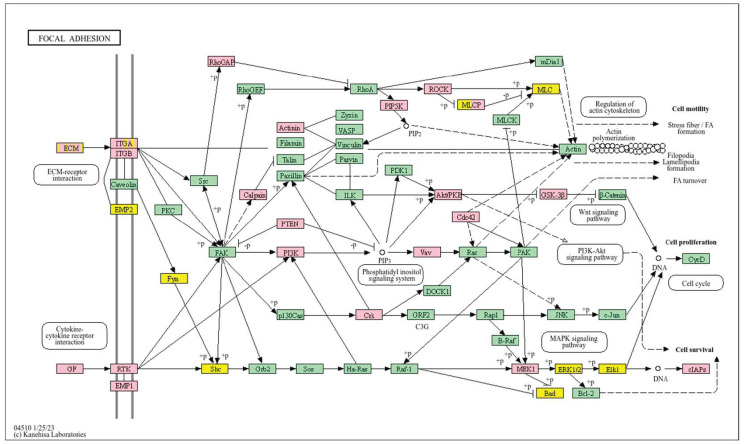
Focal adhesion signalling pathway in basal subtype with prognostic implications. The focal adhesion signalling pathway also varies according to the *WWOX*/*HIF1A* ratio. Activation of oncogenic pathways such as PI3K-Akt, MAPK, Ras, and ErbB promotes cell survival and proliferation, exacerbating tumour progression. Poor prognosis tumours display elevated expression of genes involved in glycolysis, mesenchymal transition, and immune evasion. Yellow denotes good prognosis, pink denotes poor prognosis, and green indicates genes with unchanged expression.

Survival analyses revealed a significant positive correlation between a higher *WWOX*/*HIF1A* ratio and improved patient outcomes. This association is linked to the regulation of metabolic processes, reduced oxidative stress, and enhanced tumour suppression (Figure 1 and Figure 2). Proper lipid metabolism and maintenance of transcriptional fidelity further contribute to better prognosis. Conversely, a lower *WWOX*/*HIF1A* ratio is associated with aggressive tumour behaviour characterized by enhanced glycolysis, angiogenesis, immune evasion, and dysregulated survival signalling pathways (Figure 3 and Figure 4).

Gene expression analysis (Supplementary Table S2) underscored the prognostic relevance of the *WWOX*/*HIF1A* ratio across multiple gene categories. Poor prognosis correlated with higher expression of *HIF1A* and extracellular matrix genes such as *CCR7*, *PLEKHA2*, and *KDR*, as well as mesenchymal markers *FOXC2*, *DDR2*, and *CDH11*. These genes had negative log2 fold change (*log2fc*), indicating they are upregulated in poor prognosis tumours.

In contrast, good prognosis tumours exhibited significantly higher WWOX expression (*log2fc* = 1.17), resulting in a markedly increased *WWOX*/*HIF1A* ratio (*log2fc* = 1.86). Epithelial genes also differed by prognosis: poor outcomes were linked to elevated expression of *MUC1*, *LAMA3*, *LAMA1*, and *LAMA2*. Glycolytic (Warburg effect-related) genes such as *PIK3CG*, *PRKCB*, *LDHAL6A*, and *SLC2A1* were upregulated in poor prognosis cases. Conversely, oxidative metabolism genes like *OGDHL* were elevated alongside *WWOX* in good prognosis tumours.

In basal breast cancer cases with a high *WWOX*/*HIF1A* ratio, pathways favouring oxidative phosphorylation (Complexes I–V: *NDUFA/B/C/S/V*, *SDHC*, *COX*, *ATP6* family) were enriched, reducing reliance on glycolysis and limiting tumour proliferation (Figure 2 and Figure 4). Enhanced lipid metabolism, including the linoleic acid pathway, prevented the accumulation of tumour-promoting metabolites. Transcriptional and translational fidelity was maintained through robust RNA polymerase and ribosome function (MRPL, MRPS, RPL, FAU proteins), as well as mRNA surveillance mechanisms involving RNA export factors (*NXF1*, *NXT1*), pre-mRNA processing proteins (*CPSF4*, *CSTF3*), RNA-binding proteins (*FUS*, *MAGOH*), and phosphatases (*PPP1CA*, *PPP2R2B*). Balanced nucleotide and nitrogen metabolism supported DNA and RNA synthesis without promoting excessive proliferation. Additionally, stress response pathways enhanced detoxification and DNA repair, reducing genomic instability. Proper hormonal signalling ensured controlled growth and differentiation.

Conversely, a low *WWOX*/*HIF1A* ratio corresponded to poor prognosis driven by heightened HIF1A activity and diminished tumour suppression. Key oncogenic pathways—including PI3K-Akt (*CREB1*, *GSK3B*, *RPS6KB1*, *TSC1*), MAPK (*MAP3K2*, *MAP2K1*, *EGFR*, *PDGFRB*, *CRK*, *ELK4*, *FOS*, *NFKB1*, *IL1A*), Ras (*PDGFRB*, *HGF*, *GAB1*, *PIK3CA*, *MAP2K1*, *NFKB1*, *NTF3*), and ErbB (*CDKN1A*, *EGFR*, *ERBB2*)—were activated, promoting proliferation and survival. Hypoxia-induced HIF1 signalling (*EP300*, *ALDOB*, *FLT1*, *HMOX1*) drove angiogenesis and metabolic adaptation. Dysregulated cell adhesion and ECM interactions (*HSPG2*, *RELN*, *GNA13*, *CHRM4*, *F2R*, *MAP2K1*, *RDX*) enhanced invasiveness, while TGF-β pathway activation (*SMAD1/2*, *BMP6*, *BMPR2*, *INHBA*, *DCN*, *PITX2*, *FST*, *RPS6KB1*) promoted EMT and migration (Figure 2). Immune evasion was evident through upregulation of *NFKB1*, *IFNAR1*, *TLR4*, *IL1A/B/R1*, and *NOS2*, facilitating tumour escape from immune surveillance.

To further assess the prognostic relevance of the *WWOX*/*HIF1A* ratio and related gene signatures in Table 1, multivariate Cox proportional hazards regression was performed on the basal breast cancer cohort (*n* = 95, events = 11) (Supplementary Figure S1). Tumour stage emerged as a significant independent predictor of survival, with a hazard ratio (HR) of 11.35 (95% confidence interval [CI]: 2.11–61.05, *p* = 0.005), indicating markedly worse outcomes for patients with higher stage disease. *WWOX* expression was also significantly associated with improved survival (HR = 1.014 per unit increase, 95% CI: 1.001–1.027, *p* = 0.034). Neither patient age (HR = 1.05, 95% CI: 0.98–1.12, *p* = 0.20) nor the *WWOX*/*HIF1A* ratio (*HR* ≈ 1.18 × 10^−13^, *p* = 0.17) showed statistically significant associations with survival. Other variables, including *HIF1A* (*p* = 0.55), *CD81*, *GATA3* (trend, *p* = 0.089), and *MGMT*, did not significantly predict outcomes. The overall model demonstrated good predictive accuracy with a concordance index of 0.80 (standard error = 0.078).

These results suggest that clinical stage and WWOX expression are the strongest independent prognostic factors in basal breast cancer within this cohort. Although the *WWOX*/*HIF1A* ratio did not reach statistical significance, its large effect estimate and biological associations warrant further investigation in larger cohorts to clarify its prognostic utility.

### 3.2. Breast Cancer-HER2 Subtype

**Figure 5:**
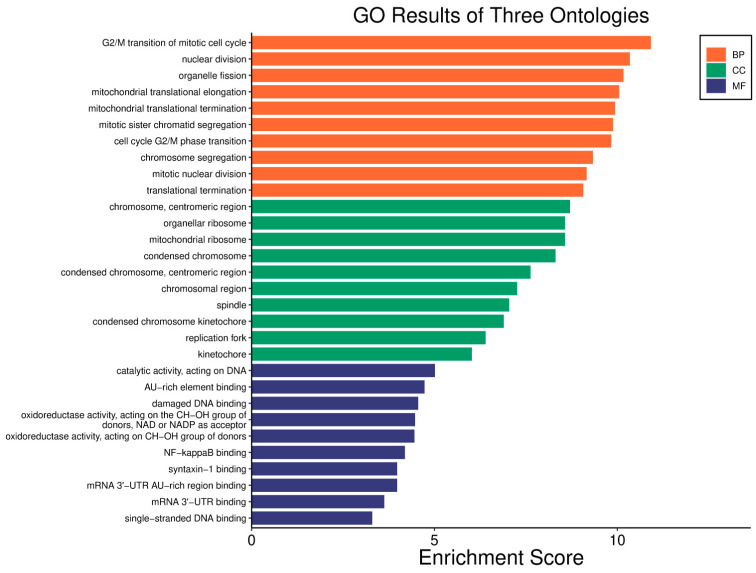
HER2 subtype with high *WWOX*/*HIF1A* ratio: good prognosis ontology. Patients with a high *WWOX*/*HIF1A* prognostic ratio exhibited favourable clinical outcomes. This good prognosis profile is characterized by molecular features that support tumour suppression and metabolic homeostasis. BP—Biological process, CC—Cellular Component, MF—Molecular Function.

**Figure 6:**
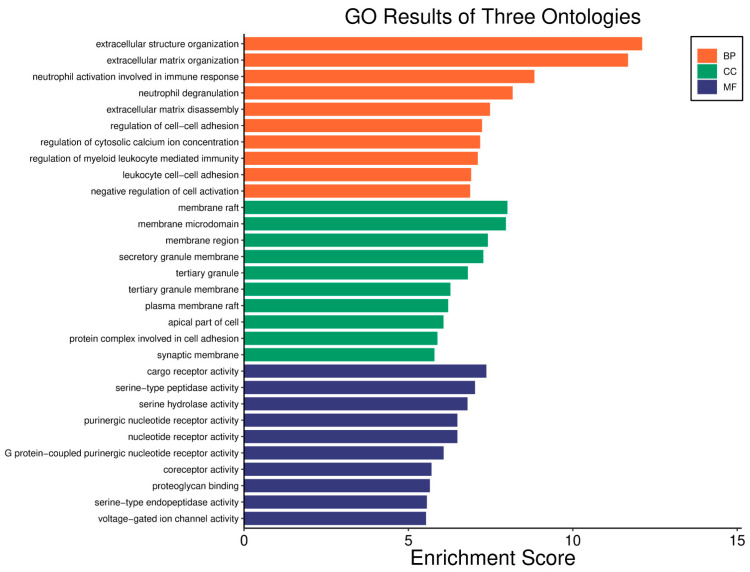
HER2 subtype with low *WWOX*/*HIF1A* ratio: poor prognosis ontology. Conversely, patients with a low *WWOX*/*HIF1A* prognostic ratio showed poor survival outcomes. This adverse prognosis is associated with molecular signatures promoting tumour aggressiveness and progression. BP—Biological process, CC—Cellular Component, MF—Molecular Function.

The *WWOX*/*HIF1A* ratio is significantly higher in the good prognosis cohort (*log2fc* = 1.8), indicating that a favourable balance between these genes correlates with improved clinical outcomes (Supplementary Table S3). Figure 5 shows gene ontology for the high *WWOX*/*HIF1A* ratio group linked to clinical outcomes, while Figure 6 presents ontology analysis for the low *WWOX*/*HIF1A* ratio group, indicating poor prognosis, both in the HER2 subtype.

Within the ECM gene category, TGFB2 is upregulated in poor prognosis tumours compared to good prognosis (*log2fc* = −0.72), highlighting its role in promoting tumour progression. Epithelial markers such as *KRT5* and *LAMA1* also show elevated expression in the poor prognosis cohort (*log2fc* = −0.94 and −0.98, respectively), suggesting their contribution to a more aggressive phenotype. Conversely, *WWOX* expression is significantly higher in the good prognosis group (*log2fc* = 1.48), reinforcing its tumour-suppressive function.

**Figure 7:**
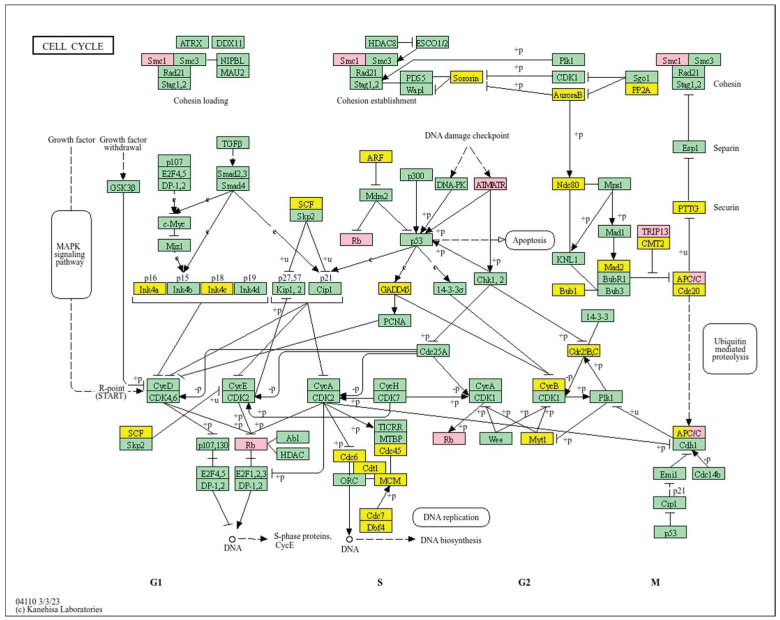
Cell cycle pathway in HER2 subtype with prognostic implications. The cell cycle pathway is differentially expressed in the HER2 breast cancer subtype, stratifying prognosis based on the *WWOX*/*HIF1A* ratio. Yellow indicates good prognosis, pink represents poor prognosis, and green indicates genes with unchanged expression.

Analysis of cell cycle (Figure 7) reveals that a high *WWOX*/*HIF1A* ratio enhances DNA repair and replication pathways, including base excision repair (*MCM3/7*, *PRIM1/2*, *RFC2/3/4*, *FEN1*, *POLB*, *MUTYH*, *SMUG1*, *APEX2*, *NEIL3*, *DDB2*), homologous recombination and mismatch repair (*RAD51C*, *RBBP8*, *RPA2/3*, *BLM*, *MUS81*, *RAD54L*, *EME1*), and nucleotide excision repair (*RAD23A*). These mechanisms maintain genomic stability and prevent mutations that drive tumour progression. Proper cell cycle regulation is supported by genes such as *GADD45G*, *CCNB1/2/3*, *ANAPC13*, *PKMYT1*, and *CDKN2A/C*, ensuring controlled proliferation. Metabolic flexibility is favoured through pathways involving pyruvate, fructose, mannose, and galactose metabolism, promoting oxidative metabolism over glycolysis. Elevated WWOX expression may also induce senescence in premalignant cells, limiting tumour formation. Additionally, intact motor protein and ribosome functions (MRPs, RPS27L, FAU) support cellular homeostasis and prevent malignant transformation.

**Figure 8:**
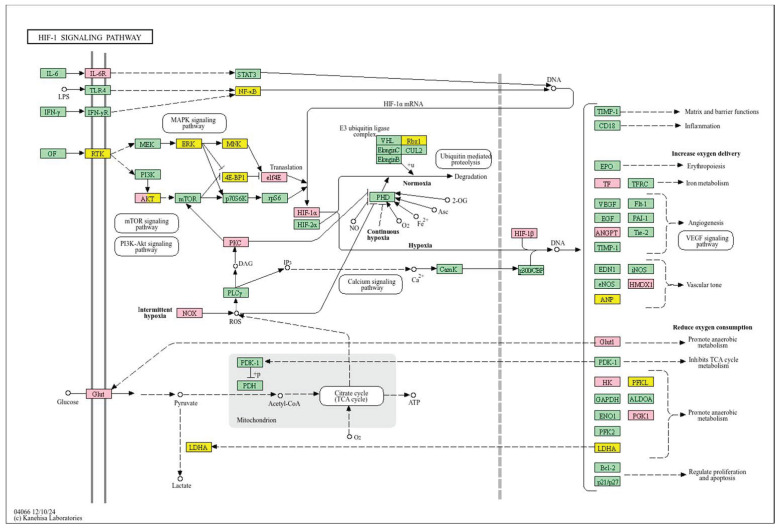
HIF1 signalling pathway in HER2 subtype with prognostic implications. The HIF1 signalling pathway also varies according to the *WWOX*/*HIF1A* ratio in HER2 tumours. Poor prognosis tumours show elevated HIF1A levels accompanied by upregulation of glycolytic enzymes such as PIK3CG and VEGFA, supporting metabolic adaptations favouring tumour growth. In contrast, good prognosis cases maintain higher WWOX expression, suggesting its role in counteracting metabolic reprogramming associated with malignancy. Yellow denotes good prognosis, pink denotes poor prognosis, and green indicates genes with unchanged expression.

In contrast, poor prognosis tumours with low *WWOX*/*HIF1A* ratios activate pathways that promote aggressive tumour behaviour. These include TGF-β/BMP signalling (*BMPs*, *ID1*, *TGIF2*, *CHRD*, *DCN*, *FBN1*) (Supplementary File S1), alterations in cytoskeletal dynamics and cell adhesion molecules (CAMs, integrins, FGF, APC2) that enhance invasion and metastasis, and chronic inflammatory signalling driven by HIF1A overexpression via the HIF1 signalling pathway (Figure 8). Stem cell regulatory pathways are also activated, increasing cancer cell stemness and contributing to therapy resistance and recurrence. Dysregulated cytokine–cytokine receptor interactions (*CXCL7*, *CDCs*, *CCR4/6/8*, *CCL22*, *XCL1*) further promote immune evasion and metastatic potential.

Multivariate Cox proportional hazards regression (*n* = 57, events = 10) assessed the independent prognostic value of the ratio alongside clinical factors and gene expressions (Supplementary Figure S2). Age (HR = 1.21 per year increase, 95% CI: 1.04–1.40, *p* = 0.012) and tumour stage (HR = 5.73, 95% CI: 1.06–31.05, *p* = 0.043) were significant predictors of survival. *WWOX* expression showed a trend toward significance (HR = 1.007, 95% CI: 0.999–1.016, *p* = 0.088). The *WWOX*/*HIF1A* ratio estimated an extremely large hazard ratio (HR = 3.72 × 10^−12^) with a wide confidence interval (95% CI: 6.41 × 10^−31^ to 2.15 × 10^7^) and did not reach statistical significance (*p* = 0.23), likely reflecting the small sample size and presence of outliers. Other genes included in the model from Table 1 did not show significance. The model demonstrated strong predictive ability (concordance index = 0.918).

### 3.3. Breast Cancer-Luminal A Subtype

In the Luminal A subtype, a higher *WWOX*/*HIF1A* ratio is paradoxically associated with poor prognosis. Metabolism-related genes reflect this trend: the *WWOX*/*HIF1A* ratio is significantly higher in poor-prognosis cases compared to good-prognosis cases (*log2fc* = −2.04) (Supplementary Table S4). Specifically, *WWOX* expression is markedly reduced in poor-prognosis tumours (*log2fc* = −1.34), while *HIF1A* expression is elevated (*log2fc* = 1.09). Other metabolism-related genes, such as *LDHC* (*log2fc* = −1.08), *ALDH3A1* (*log2fc* = −0.64), and *G6PC2* (*log2fc* = −0.62), are downregulated in poor prognosis cases, potentially contributing to worse outcomes. Conversely, genes including *HK3* (*log2fc* = 0.54), *LDHAL6A* (*log2fc* = 0.55), *ADH6* (*log2fc* = 0.65), *PRKCB* (*log2fc* = 0.82), and *ADH1C* (*log2fc* = 1.22) are upregulated and associate with better prognosis.

ECM-related genes follow a similar pattern. The chemokine receptor *CCR7* shows increased expression in good-prognosis cases (*log2fc* = 0.88). Mesenchymal markers such as *CDH2* (*log2fc* = 0.57) and *DDR2* (*log2fc* = 0.63) are elevated in poor-prognosis tumours, suggesting that upregulation of mesenchymal genes may contribute to more aggressive tumour phenotypes.

**Figure 9:**
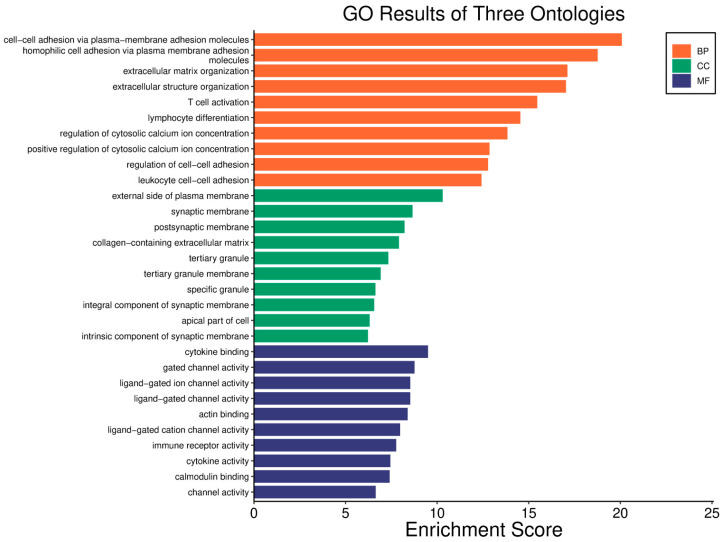
Luminal A subtype with low *WWOX*/*HIF1A* ratio: good prognosis ontology. Patients with a low *WWOX*/*HIF1A* prognostic ratio exhibited favourable clinical outcomes, characterized by molecular signatures associated with better tumour control. BP—Biological process, CC—Cellular Component, MF—Molecular Function.

**Figure 10:**
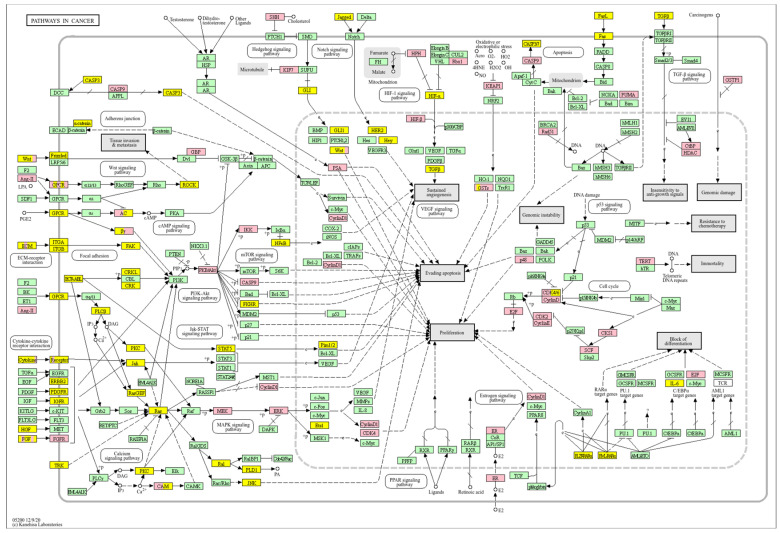
Differential expression in signalling pathways in luminal A subtype. Several signalling pathways are differentially expressed in the Luminal A subtype, with prognostic implications based on the *WWOX*/*HIF1A* ratio. Good prognosis tumours predominantly show enrichment in ECM receptor, TNF, JAK-STAT, and PI3K-AKT signalling pathways. In contrast, poor-prognosis tumours are associated with upregulation of cell cycle and genomic damage pathways. Yellow indicates good prognosis, pink signifies poor prognosis, and green indicates genes with unchanged expression.

A low *WWOX*/*HIF1A* ratio in Luminal A tumours correlates with pathways linked to favourable prognosis, including modulation of metabolic pathways, reduced glycolysis, and regulation of cell survival and proliferation (Figure 9). Downregulation of this axis can disrupt glucose metabolism and predispose cells to metabolic dysfunctions, influencing tumour behaviour and patient outcomes. Key pathways such as PI3K-Akt (involving *FGFs*, *CDK6*, *ERBB2*, *NRAS*, *IGF1R*, *HGF*) are critical for cell survival and metabolism. Their activation in Luminal A tumours may promote controlled cell growth without excessive proliferation, contributing to favourable clinical outcomes. Interactions between cytokines and chemokines and their signalling pathways regulate immune responses and cell recruitment into the TME, enhancing antitumour immunity. Additional hematopoietic and immunological pathways support an immune microenvironment that may inhibit tumour progression. Cell adhesion molecules, focal adhesion, and regulation of the actin cytoskeleton (*COL4/6*, *LAMA2/4*, *LAMB1*, *LAMC1*, *ITGAs*, *VCL*, *PXN*) are essential for maintaining cellular integrity and preventing metastasis, thereby contributing to better survival. Other pathways, such as JAK-STAT signalling, fatty acid degradation, and retinol metabolism, also appear to maintain cellular homeostasis and reduce tumour aggressiveness when WWOX expression is relatively high compared to *HIF1A* (Figure 10). WWOX has been shown to influence HIF1α activity, which is pivotal in cellular responses to hypoxia.

**Figure 11:**
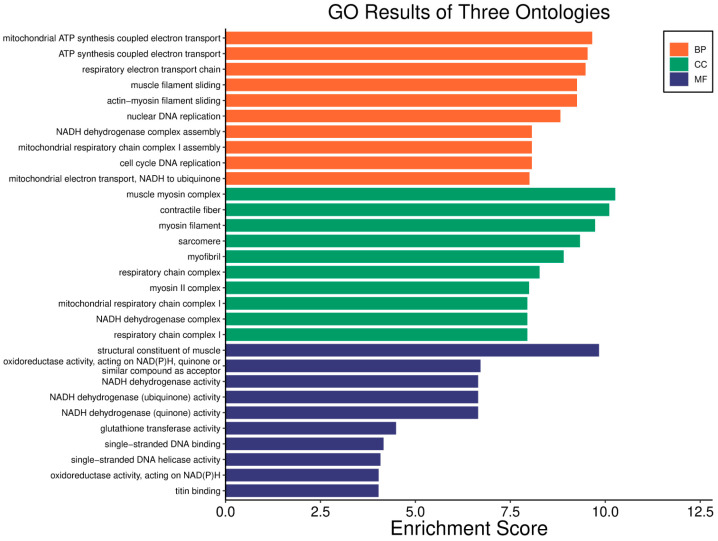
Luminal A subtype with high *WWOX*/*HIF1A* ratio: poor prognosis ontology. Conversely, patients with a high *WWOX*/*HIF1A* prognostic ratio showed poorer survival outcomes, linked to molecular features promoting tumour aggressiveness. BP—Biological process, CC—Cellular Component, MF—Molecular Function.

**Figure 12:**
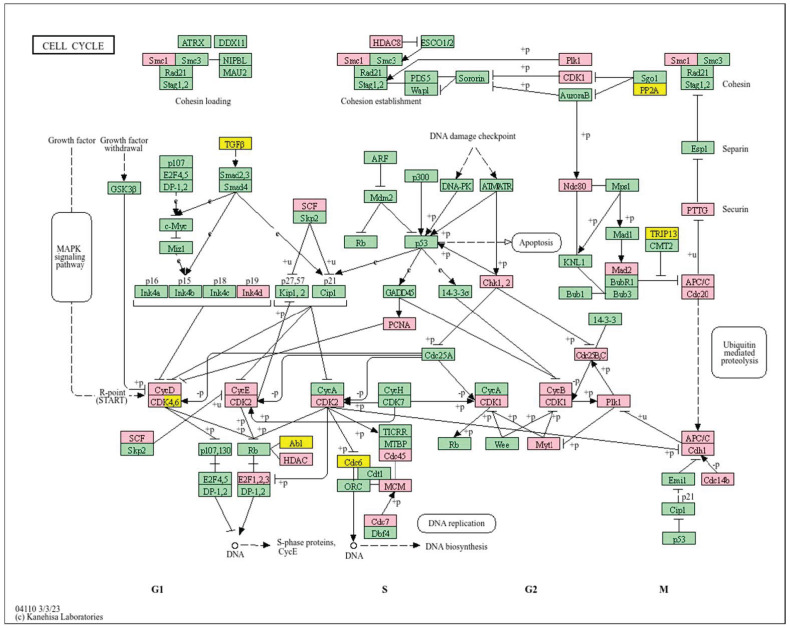
Cell cycle genes differentially expressed in luminal A subtype. Cell cycle-related genes exhibit differential expression linked to prognosis in the Luminal A subtype. Yellow indicates favourable prognosis, pink denotes less favourable outcomes, and green indicates genes with unchanged expression.

In contrast, a high *WWOX*/*HIF1A* ratio is associated with pathways indicative of poor prognosis, including genomic instability, uncontrolled proliferation, and tumour progression (Figure 11). Dysregulation of DNA replication and cell cycle pathways (Figure 12) leads to unchecked tumour growth, while deficiencies in mismatch repair and base excision repair contribute to genomic instability and mutation accumulation. Oxidative phosphorylation components (*NDFs*, *COXs*, *TCIRG1*, *LHPP*) and chemical carcinogenesis pathways involving reactive oxygen species (*CYP1B1*, *GSTs*, *AKT2*, *IKBKB*, *MAPK3*, *MAP2K2*) exacerbate DNA damage and promote tumour aggressiveness. Alterations in the p53 signalling pathway (*GTSE1*, *BBC3*, *PERP*, *CASP9*, *AIFM2*), a key regulator of cell cycle arrest and apoptosis, are linked to worse outcomes when *WWOX* is downregulated relative to HIF1A. Furthermore, pathways involved in xenobiotic metabolism (*GSTs*, *DHDH*, *AKR7A2*, *HSD11B1L*, *CYP1B1*, *UGTs*), drug metabolism, nucleotide metabolism, and homologous recombination (*RAD51*) reflect the tumour’s ability to adapt to environmental stressors and resist therapy.

To determine the independent impact of the *WWOX*/*HIF1A* ratio, age, stage, and selected gene expressions, we conducted multivariate Cox proportional hazards regression (*n* = 216, events = 18) (Supplementary Figure S3). In this model, age emerged as a significant independent predictor of survival (*HR* = 1.05 per year, 95% CI: 1.01–1.10, *p* = 0.025). Notably, the *WWOX*/*HIF1A* ratio was also independently associated with poor prognosis (HR = 2.76, 95% CI: 1.01–7.52, *p* = 0.047), confirming its relevance as a molecular risk factor in this cohort. Neither tumour stage nor individual gene expressions (*WWOX*, *HIF1A*, *HK3*, *LDHAL6A*, *ADH6*, *PRKCB*, *MYH7*, *FABP3*, *CYP4F2*, *MTHFR*) reached statistical significance (*p* > 0.1). The model demonstrated good discrimination (concordance index = 0.80, SE = 0.057). These findings suggest that, unlike other subtypes, a high *WWOX*/*HIF1A* ratio in Luminal A breast cancer is an adverse prognostic marker, independently of age and stage. This ratio integrates the effects of multiple metabolic, ECM, and cell cycle factors, highlighting potential subtype-specific roles for WWOX-HIF1A interplay in tumour biology and outcome.

### 3.4. Breast Cancer-Luminal B Subtype

Similar to the Luminal A subtype, a higher *WWOX*/*HIF1A* ratio in Luminal B tumours correlates with poor prognosis. Epithelial genes exhibit marked differences between prognostic groups (*log2fc* = −2.53), with genes such as *LAMA2* (*log2fc* = 0.61) and KRT5 (*log2fc* = 1.50) upregulated in good prognosis cases. Mesenchymal marker CDH2 also follows this trend, showing lower expression in poor prognosis tumours (*log2fc* = 0.51). ECM-related genes *PIK3R1* and *TGFB2* demonstrate contrasting profiles (*log2fc* = −0.5 and 0.61, respectively). Metabolism genes, including *PFKFB1* (*log2fc* = −0.75) and *ALDH3A1* (*log2fc* = −0.70), are downregulated in poor-prognosis cases (Supplementary Table S5).

**Figure 13:**
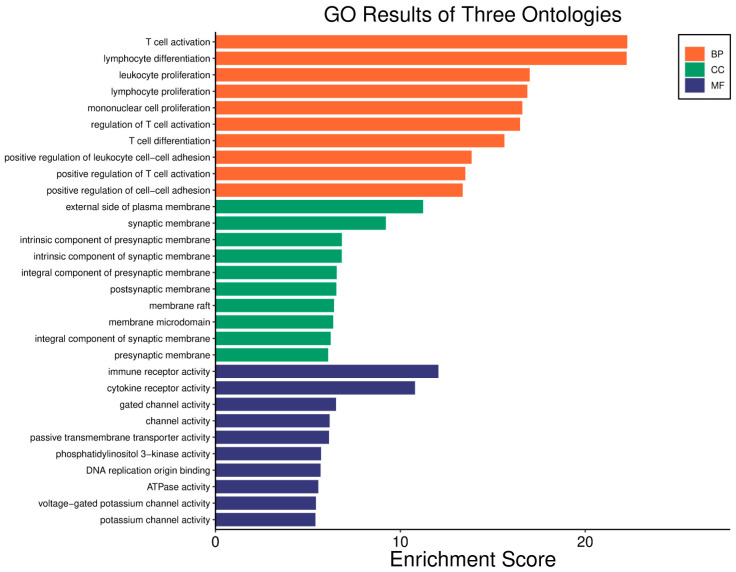
Luminal B subtype with low *WWOX*/*HIF1A* ratio: good prognosis ontology. Conversely, a low *WWOX*/*HIF1A* ratio correlates with better prognosis. Enhanced activation of T cell and B cell receptor signalling pathways promotes adaptive immunity, improving tumour surveillance and control. Elevated WWOX levels support cell adhesion molecule function, maintaining tissue architecture and preventing metastasis. BP—Biological process, CC—Cellular Component, MF—Molecular Function.

Good prognosis is associated with activation of cytokine–cytokine receptor interactions and chemokine signalling, facilitating immune cell recruitment and anti-tumour responses. The JAK-STAT pathway mediates cytokine and growth factor signalling, supporting immune function and normal cellular activity when regulated alongside WWOX. Balanced PI3K-Akt pathway activation with high WWOX levels supports normal cell function without excessive proliferation. The NF-κB signalling pathway regulates immune responses and inflammation, maintaining a balance that improves outcomes. Enhanced natural killer cell-mediated cytotoxicity contributes to anti-tumour immunity when WWOX is adequately expressed. Proper regulation of the actin cytoskeleton preserves cell shape and motility, reducing metastasis risk under favourable *WWOX*/*HIF1A* ratios. Metabolic alterations in breast tumour progression include changes in central carbon metabolism and increased glycine and proline synthesis, especially in metastatic cells (Figure 13). The cell cycle pathway is crucial for regulating proliferation; its dysregulation leads to aggressive tumour phenotypes. Adequate WWOX expression supports proper cell cycle regulation (e.g., *CHEK1*) and DNA replication (*POLA1/2*), preventing genomic instability. The ErbB pathway (*GRB2*, *CRKL*, *PAK2/3*), particularly *ERBB2*, is involved in cell proliferation and survival. Balanced activation with high WWOX expression supports normal cellular functions without promoting excessive growth. The Wnt pathway (CCND2) regulates cell differentiation and development; proper control may prevent tumour progression when WWOX is sufficient.

**Figure 14:**
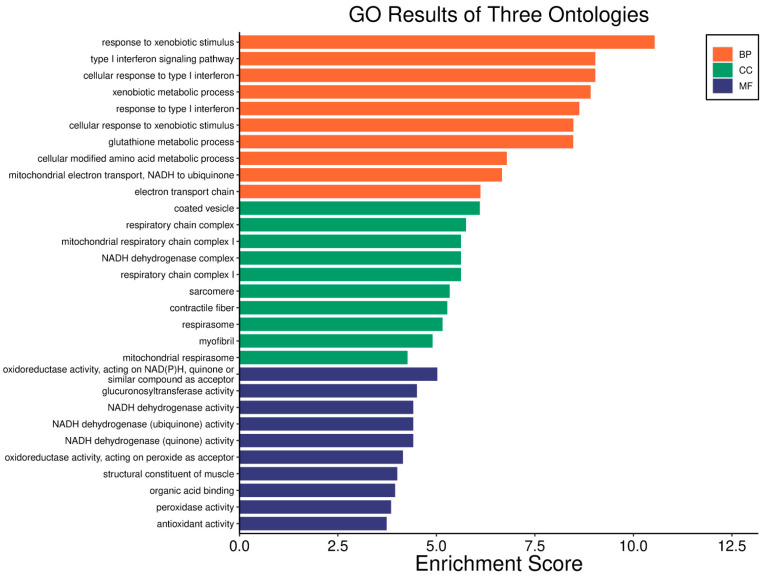
Luminal B subtype with high *WWOX*/*HIF1A* ratio: poor prognosis ontology. In the Luminal B subtype, a high *WWOX*/*HIF1A* ratio is associated with poor prognosis. Although oxidative phosphorylation (involving *ATP12A*, *ATP6AP1*, *LHPP*) is essential for energy production, its dysregulation can increase reactive oxygen species (ROS), leading to DNA damage if not adequately countered by WWOX. This effect is compounded by disruptions in glutathione metabolism, which elevate oxidative stress and contribute to cancer progression. BP—Biological process, CC—Cellular Component, MF—Molecular Function.

**Figure 15:**
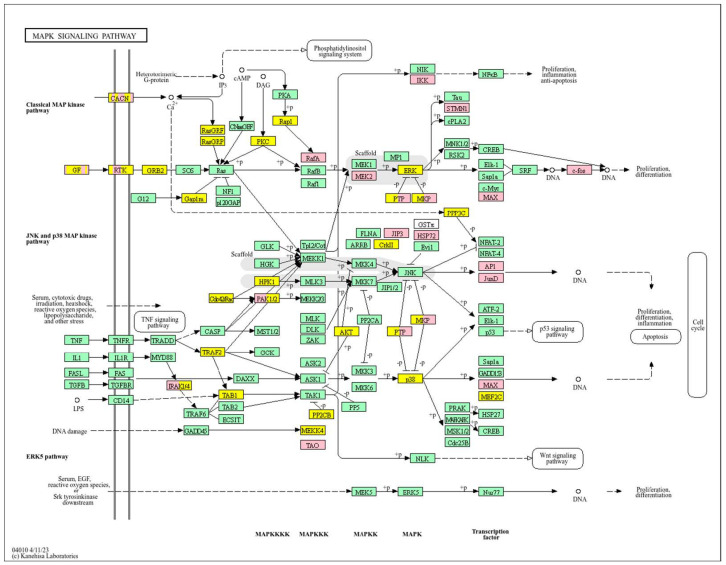
Cell cycle genes differentially expressed in luminal B subtype. Cell cycle-related genes show differential expression linked to prognosis. Yellow indicates good prognosis, pink signifies poor prognosis, and green indicates genes with unchanged expression.

A high *WWOX*/*HIF1A* ratio is linked to dysregulation of DNA repair mechanisms, including DNA replication, nucleotide excision repair (NER), and base excision repair (BER), leading to increased mutations and tumour progression due to low WWOX expression (Figure 14). The oestrogen signalling pathway, particularly relevant in Luminal B tumours, may promote proliferation and survival if not properly regulated by WWOX. The MAPK signalling pathway also contributes to uncontrolled tumour growth when dysregulated. Focal adhesion and ECM–receptor interactions become disrupted under low WWOX conditions, facilitating invasion and metastasis. Insufficient WWOX levels may impair cellular senescence, allowing damaged cells to proliferate unchecked (Figure 15). Additional pathways such as cell cycle regulation, xenobiotic metabolism by cytochrome P450 (*CYP2C9*), and nitrogen metabolism can influence cancer progression, although their direct links to the *WWOX*/*HIF1A* ratio require further investigation.

To determine the independent prognostic value of these molecular features, we performed multivariate Cox proportional hazards regression (*n* = 119, events = 14) (Supplementary Figure S4). In this analysis, tumour stage emerged as the only significant independent predictor (*HR* = 3.38, 95% CI: 1.17–9.72, *p* = 0.024), with higher stage associated with a greater risk of adverse outcome. The *WWOX*/*HIF1A* ratio demonstrated a strong trend toward poor prognosis (*HR* = 7.92, 95% CI: 0.37–168.6) but did not reach statistical significance (*p* = 0.185), likely reflecting the modest cohort size and presence of high-variance cases. Individual expression of WWOX was marginally associated with risk (*HR* = 1.003, *p* = 0.0596), while TP53I11 expression was associated with a small reduction in risk (*HR* = 0.999, *p* = 0.039). Other variables (age, HIF1A, immune or DNA repair genes) did not show independent effects. The overall model demonstrated good predictive discrimination (concordance index = 0.816).

These findings underscore the complexity of prognostic stratification in Luminal B breast cancer: while transcriptomic analysis shows the *WWOX*/*HIF1A* ratio is strongly associated with pathway changes and biological features linked to risk, in multivariate analyses, only clinical stage retains independent significance. The ratio’s effect size remains notable, suggesting that in larger or more granular cohorts, its statistical significance might become clearer.

### 3.5. Hepatocellular Carcinoma

Survival analysis in HCC reveals that a higher *WWOX*/*HIF1A* ratio is significantly associated with improved prognosis (Supplementary Table S6). Among epithelial markers, *KRT19* expression is markedly reduced in good-prognosis patients compared to poor-prognosis cases (*log2fc* = −3.10). Similarly, mesenchymal gene *ITGB6* is significantly downregulated in good-prognosis tumours (*log2fc* = −2.22), underscoring the role of mesenchymal transition in tumour aggressiveness. Metabolic genes further differentiate prognosis: *PFKFB1* expression is elevated in good-prognosis tumours (*log2fc* = −1.59), reflecting more regulated metabolism compared to aggressive tumours exhibiting metabolic dysregulation.

**Figure 16:**
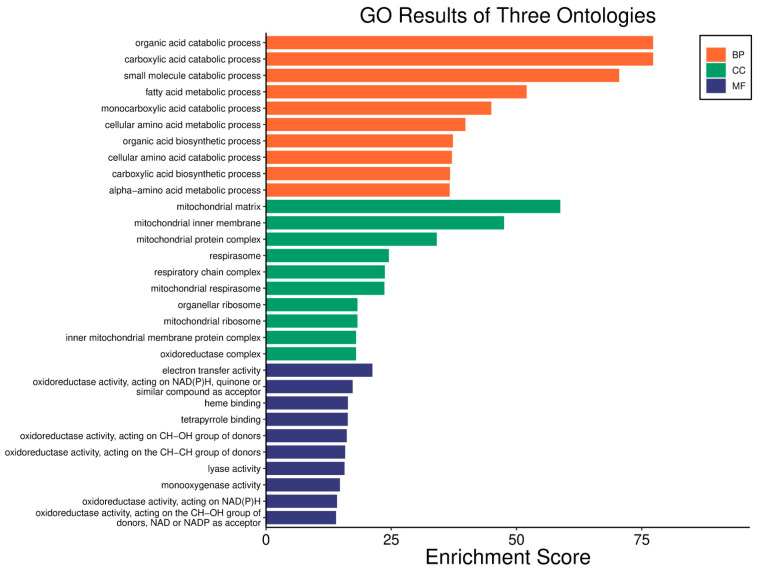
HCC with high *WWOX*/*HIF1A* ratio: good prognosis ontology. Patients exhibiting a high *WWOX*/*HIF1A* ratio demonstrate favourable clinical outcomes characterized by enhanced metabolic regulation and cellular homeostasis. BP—Biological process, CC—Cellular Component, MF—Molecular Function.

A high *WWOX*/*HIF1A* ratio in HCC activates pathways that enhance metabolic homeostasis and detoxification. Cytochrome P450 and glutathione metabolism enzymes (*CBR1*, *SULT2A1*, *HSD11B1*, *EPHX1*) neutralize carcinogens and oxidative stress, reducing the risk of carcinogenesis (Figure 16). Energy metabolism favours oxidative phosphorylation and the tricarboxylic acid (TCA) cycle (*OGDHL*, *PCK1*, *ACO1*, *FH*) over glycolysis, limiting tumour growth. Regulation of lipid and amino acid metabolism prevents toxin accumulation. The PPAR pathway (*APOA1/2/5*, *APOC3*) supports fatty acid oxidation and energy balance, which improves clinical outcomes. cAMP (*ADCY1/2/8/10*) and AMPK (*INS*, *IRS1*, *LEP*, *HNF4A*) pathways control metabolism by increasing oxidative phosphorylation and inhibiting glycolysis, consistent with observed metabolic shifts. ABC transporters (*ABCB4/5/6*) facilitate drug and metabolite clearance, influencing drug resistance and treatment efficacy.

**Figure 17:**
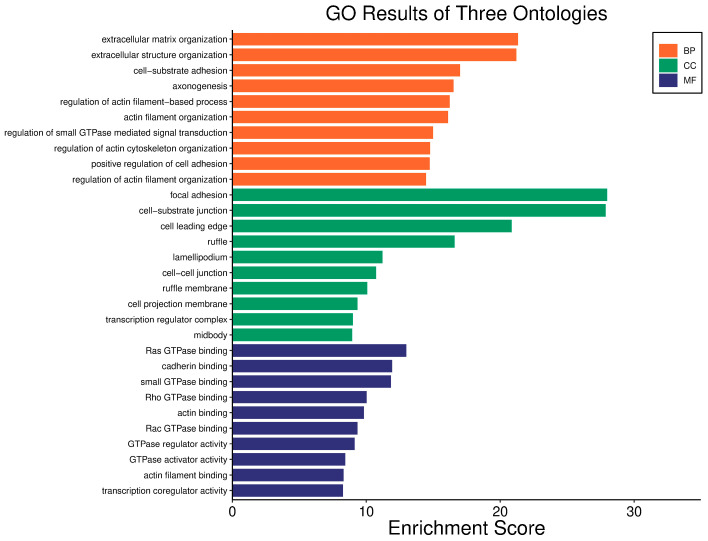
HCC with low *WWOX*/*HIF1A* ratio: poor prognosis ontology. Conversely, a low *WWOX*/*HIF1A* ratio correlates with poor prognosis, marked by aggressive tumour behaviour and adverse molecular signatures. BP—Biological process, CC—Cellular Component, MF—Molecular Function.

**Figure 18:**
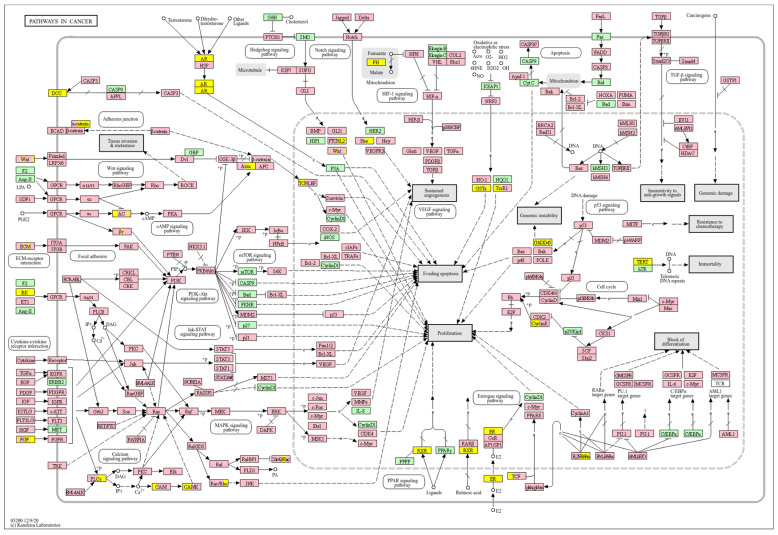
Differentially expressed pathways in HCC stratified by *WWOX*/*HIF1A* ratio. Key signalling pathways vary significantly with prognosis. Yellow indicates good prognosis, pink signifies poor prognosis, and green indicates genes with unchanged expression.

A low *WWOX*/*HIF1A* ratio is associated with poor prognosis in HCC, primarily through increased HIF1A expression. HIF1A activates the oncogenic PI3K-Akt (*IGF1R*, *VEGFA*), MAPK (*KRAS*, *NRAS*), and VEGF pathways, promoting proliferation, cell survival, and angiogenesis under hypoxic conditions (Figure 17). It also promotes tumour vascularization, blocks apoptosis, and promotes rapid growth through cell cycle deregulation. Additionally, it activates immune evasion mechanisms, including PD-L1/PD-1 checkpoint pathways (*CD274*), and tumour microenvironment remodelling and cell invasion by regulating adhesion molecules and promoting EMT. Other pathways, such as Ras (*RAF1*, RALBP1, *RASSF1*), TGF-β (*CREB3L2/3*, *CREB5*, *TCL1A*), Rap1, Hippo, and axonal guidance, further facilitate tumour migration and spread (Figure 18).

Multivariate Cox proportional hazards regression was performed (*n* = 370, events = 89) (Supplementary Figure S5). Age at diagnosis emerged as a significant independent predictor of survival (*HR* = 1.03 per year, 95% CI: 1.01–1.05, *p* = 0.0005). The *WWOX*/*HIF1A* ratio was associated with a protective effect (*HR* = 0.58, 95% CI: 0.23–1.48), though this did not reach statistical significance (*p* = 0.26). Other variables, including individual *WWOX* and *HIF1A* expression, *MMP1*, *CD36*, *FGF19*, *ITGB1*, *HNF4A*, were not significant predictors (*p* > 0.05). Overall, the model demonstrated moderate discrimination (concordance index = 0.64).

These results suggest that while a high *WWOX*/*HIF1A* ratio marks favourable molecular features and reduced tumour aggressiveness in HCC, clinical factors—especially age—remain the strongest independent prognostic indicators. The ratio’s large effect estimates but lack of statistical significance in multivariate analysis likely reflects both biologic stratification and the influence of clinical covariates and cohort heterogeneity, highlighting the need for validation in larger or independent datasets.

### 3.6. Glioblastoma

**Figure 19:**
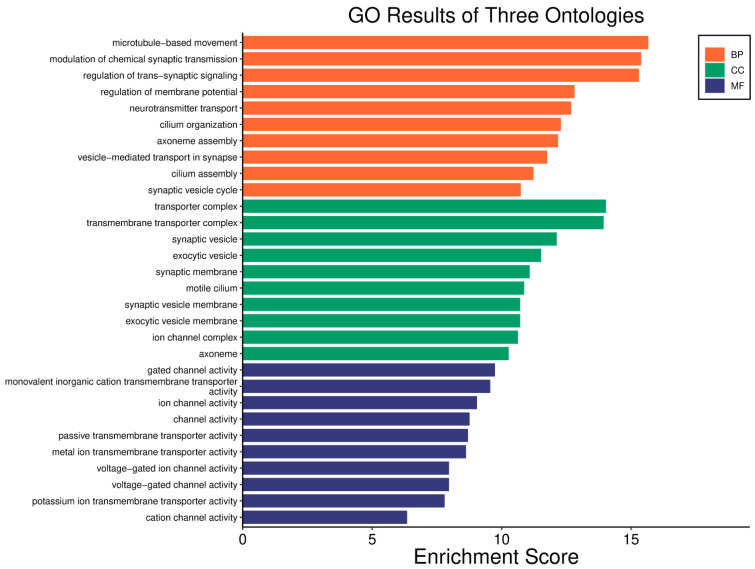
Glioblastoma with high *WWOX*/*HIF1A* ratio: good prognosis ontology. This figure illustrates molecular features and pathways enriched in GBM tumours exhibiting a high *WWOX*/*HIF1A* ratio, associated with favourable prognosis. BP—Biological process, CC—Cellular Component, MF—Molecular Function.

In GBM, a higher *WWOX*/*HIF1A* ratio is linked to better patient outcomes, reflecting the tumour’s biological behaviour and signalling pathways (Figure 19). WWOX expression is increased (*log2fc* = 0.66) and HIF1A expression is decreased (*log2fc* = −0.79) in cases with a favourable prognosis, suggesting better outcomes by promoting apoptosis and limiting adaptation to hypoxia. A high *WWOX*/*HIF1A* ratio (*log2fc* = 1.22) suggests a favourable balance between these processes (Supplementary Table S7). Epithelial genes, such as *KRT8* (*log2fc* = −0.77), *LAMA2* (*log2fc* = −0.69), and *COL4A1* (*log2fc* = −0.54), are downregulated in cases with a favourable prognosis (Supplementary Table S7). Many mesenchymal genes exhibit downregulation in good prognosis cases, including *CDH11* (*log2fc* = −0.51) and *SNAI1* (*log2fc* = −0.89), which are associated with EMT. Several metabolism-related genes exhibit decreased expression in good prognosis cases, including *PIK3CG* (*log2fc* = −1.03) and *HK3* (*log2fc* = −0.92). Upregulated genes such as *ACSS1* (*log2fc* =0.50), *PFKFB2* (*log2fc* = 0.57), *GCK* (*log2fc* =0.59), and *PGAM2* (*log2fc* =0.59) indicate potential metabolic adaptations that may support tumour growth, though their impact is less pronounced compared to the *WWOX*/*HIF1A* ratio.

**Figure 20:**
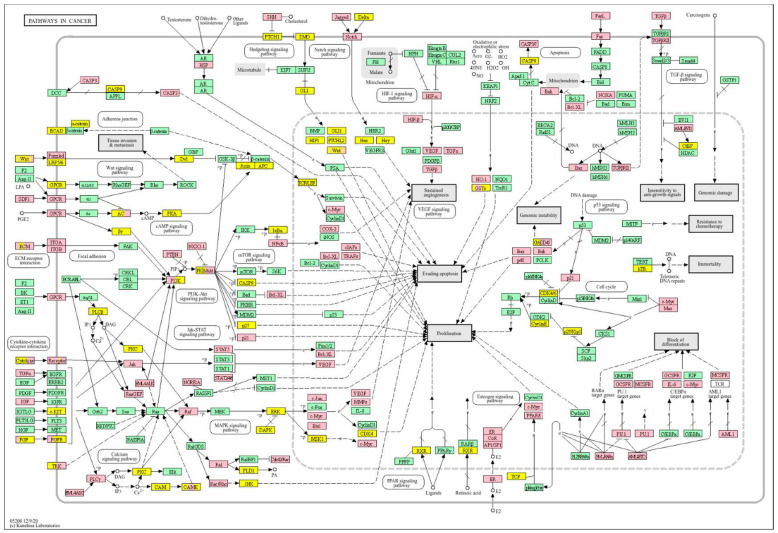
Differentially expressed pathways in glioblastoma based on *WWOX*/*HIF1A* ratio. This figure summarizes key signalling pathways differentially regulated in GBM tumours stratified by *WWOX*/*HIF1A* ratio. Yellow indicates good prognosis, pink signifies poor prognosis, and green indicates genes with unchanged expression. The figure emphasizes the balance between apoptotic and hypoxia-adaptive mechanisms influencing clinical outcomes.

Pathway analysis in good prognosis cases (Figure 20) revealed enrichment of cAMP (*MAPL3/10*, *PIK3R1*, *PLD2*, *PRKACB*), hedgehog, and Wnt (*CACYBP*, *MAK10*, *CER1*, *NOTUM*) pathways, which support apoptosis and cell regulation. Motor proteins (*TUBA1C*, *TUBB6*, *DYNLT3*, *KLC3*) and carbon metabolism (*PGP*, *HAO1*, *PRPS1*, *HIBCH*, *RGN*) help maintain cell structure and energy production.

**Figure 21:**
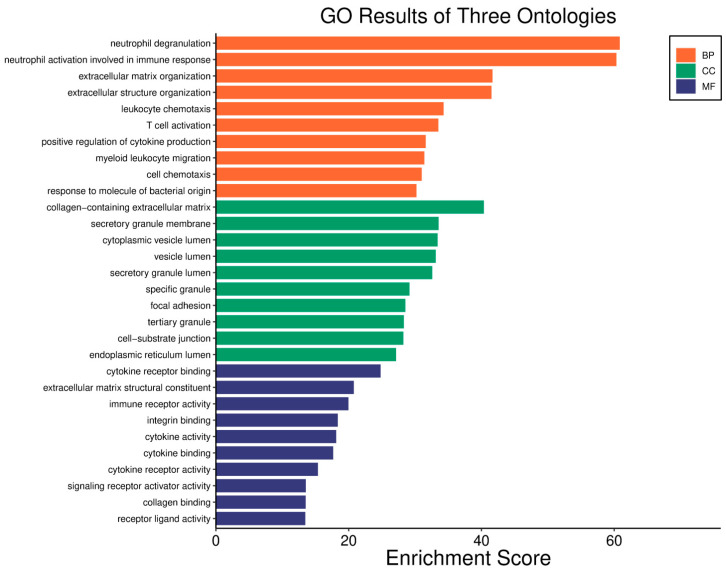
Glioblastoma with low *WWOX*/*HIF1A* ratio: poor prognosis ontology. This figure depicts the molecular landscape of GBM tumours with a low *WWOX*/*HIF1A* ratio and poor prognosis. BP—Biological process, CC—Cellular Component, MF—Molecular Function.

In contrast, a low *WWOX*/*HIF1A* ratio correlates with pathways linked to poor prognosis (Figure 21), such as TNF signalling, HIF1 (*ICAM1*), NF-kappa B (*RELB*, *IL1B*, *BBCL2A1*, *BLC2L1*) signalling, the PI3K-Akt (*ITGA/Bs*) pathway, and the p53 signalling pathway. These pathways are frequently activated in GBM and are associated with increased cell survival, proliferation, and resistance to apoptosis (*FAS*, *CASP3/7/10*, *RIPK1*, *CASP8/10*). These pathways drive increased cell survival, proliferation, and resistance to apoptosis (*FAS*, *CASP3/7/10*, *RIPK1*, *CASP8/10*). Overexpression of HIF1A under hypoxic conditions further exacerbates tumour aggressiveness by promoting adaptive survival mechanisms.

In this multivariate Cox proportional hazards regression model (*n* = 158, events = 106) (Supplementary Figure S6), increasing patient age was the strongest and only statistically significant independent predictor of survival (*HR* = 1.05 per year, 95% CI: 1.03–1.07, *p* = 8.04 × 10^−6^). The *WWOX*/*HIF1A* ratio demonstrated a large effect estimate (HR = 56.1, 95% CI: 0.03–99,080), but this did not reach statistical significance (*p* = 0.29), likely due to wide confidence intervals and high data variability. Neither *WWOX* (*HR* = 0.9987, 95% CI: 0.996–1.001, *p* = 0.26), *HIF1A* (*HR* = 1.00, *p* = 0.83), nor other included variables were significant independent prognosticators, with the exception of *CDK4* (*HR* close to 1, *p* = 0.047) and *CDKN1B* (*HR* = 0.9996, *p* = 0.014), which showed borderline significance.

The multivariate model had moderate predictive performance (concordance index = 0.686, SE = 0.028), and global tests for model fit were significant (likelihood ratio test *p* = 6 × 10^−5^).

### 3.7. Low Grade Glioma

In LGG, a higher *WWOX*/*HIF1A* ratio (*log2fc* = 1.62) correlates with a better prognosis, as confirmed by gene expression data (Supplementary Table S8). *WWOX* is elevated (*log2fc* = 0.72), and *HIF1A* expression is decreased (*log2fc* = −1.26). ECM genes, e.g., *TGFB2* (*log2fc* = −1.43), are downregulated in cases with a good prognosis, indicating a complex role of the ECM. Elevated expression of epithelial markers, such as *CDH1* (*log2fc* = 5.73) and *KRT19* (*log2fc* = 1.67), indicates the importance of epithelial integrity in LGG progression. The expression of mesenchymal genes, *MMP9* (*log2fc* = −3.34) and *VIM* (*log2fc* = −1.69), is reduced, which is associated with lower aggressiveness. Among metabolic genes, *HK3* (*log2fc* = −1.64) and *VEGFA* (*log2fc* = −1.29) are downregulated, while *ACSS1* (*log2fc* = −0.80) and *PFKFB2* (*log2fc* = −0.64) are upregulated, suggesting metabolic adaptation.

**Figure 22:**
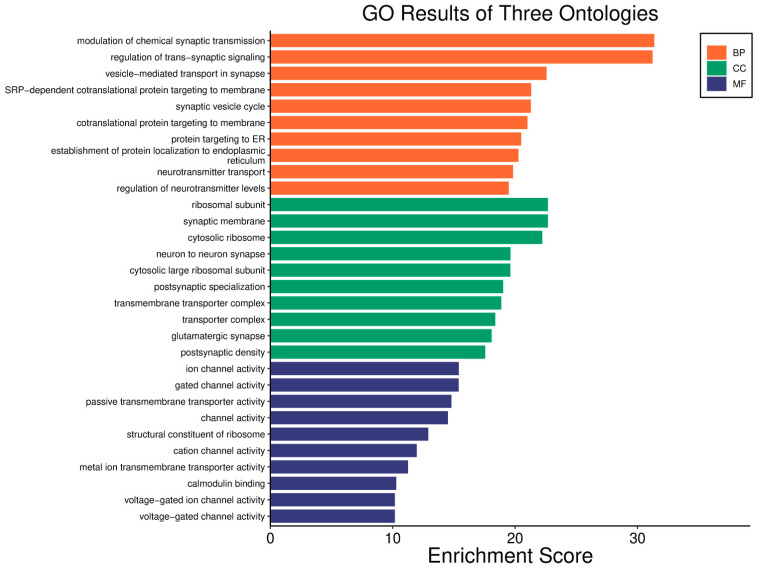
LGG with high *WWOX*/*HIF1A* ratio: good prognosis ontology. This figure depicts molecular features enriched in low-grade gliomas exhibiting a high *WWOX*/*HIF1A* ratio, associated with favourable prognosis. BP—Biological process, CC—Cellular Component, MF—Molecular Function.

**Figure 23:**
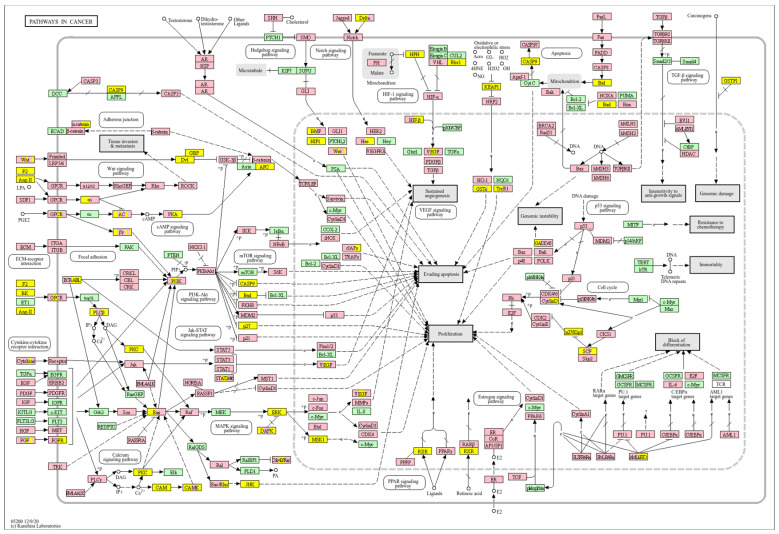
Differentially expressed pathways in LGG based on *WWOX*/*HIF1A* ratio. This figure summarizes key signalling and metabolic pathways differentially regulated in LGG tumours stratified by *WWOX*/*HIF1A* ratio. Yellow indicates pathways enriched in good-prognosis tumours, pink denotes pathways upregulated in poor-prognosis tumours, emphasizing the balance between metabolic regulation, immune response, and tumour aggressiveness, and green indicates genes with unchanged expression.

**Figure 24:**
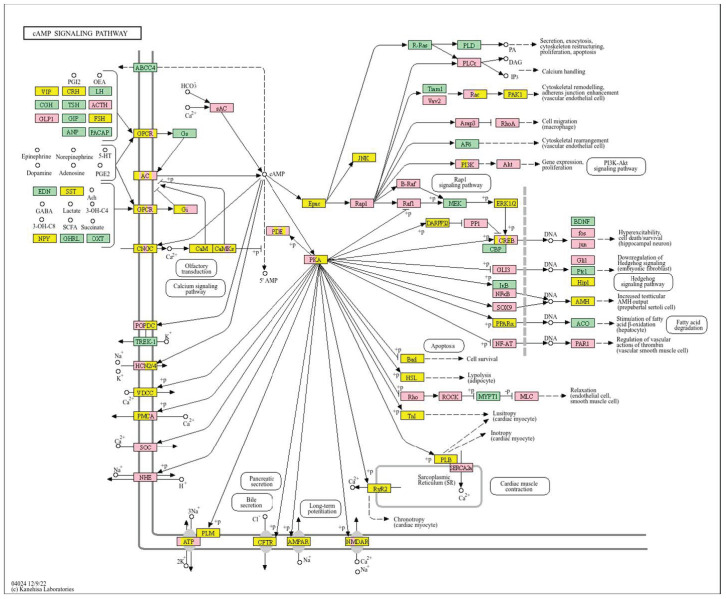
This figure provides a detailed view of the cAMP pathway’s involvement in prognosis for lower-grade glioma (LGG), emphasizing how the *WWOX*/*HIF1A* ratio influences tumour cell growth and survival. Pathway activity is associated with patient outcomes: yellow indicates pathways linked to good prognosis, pink denotes those associated with poor prognosis, andgreen indicates genes with unchanged expression.

In LGG, pathways associated with a good prognosis include oxidative phosphorylation (*LHPP*, *PPA1*, *CYC1*), supported by a high *WWOX*/*HIF1A* ratio, which improves energy metabolism and reduces tumour aggressiveness (Figure 22). Furthermore, proper regulation of carbon metabolism pathways (*TALDO1*, *PGP*, *GPT*, *GPT2*, *ME1*) limits metabolic flexibility, supporting treatment. Activation of the Wnt pathway (*TBL1Y*, *LGR5*, *CCND3*) promotes normal proliferation and inhibits neoplastic transformation with a favourable *WWOX*/*HIF1A* ratio (Figure 23). AMPK (*CAMKK2*, *CREBs*, *PPP2R5A/B*, *PPP2RC/D*) and cAMP (*BAD*, *PLN*) pathways regulate energy balance and induce autophagy and apoptosis, inhibiting tumour cell growth (Figure 24).

**Figure 25:**
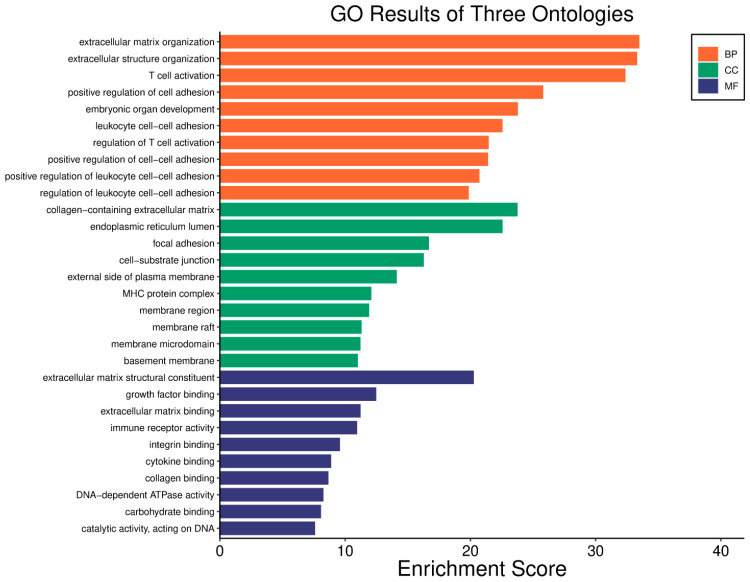
LGG with low *WWOX*/*HIF1A* ratio: poor prognosis ontology. This figure illustrates the molecular landscape of LGG tumours with a low *WWOX*/*HIF1A* ratio and poor prognosis. It highlights alterations in immune-related pathways that promote tumour progression and immune evasion. BP—Biological process, CC—Cellular Component, MF—Molecular Function.

**Figure 26:**
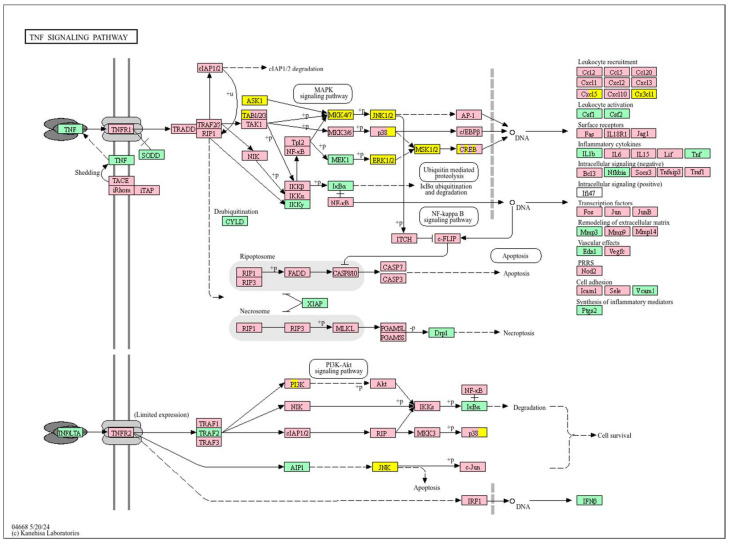
Additional differentially expressed pathways in LGG stratified by *WWOX*/*HIF1A* ratio. This figure further details pathways associated with prognosis in LGG, highlighting the role of energy metabolism, apoptosis, and immune signalling in determining clinical outcomes. Yellow denotes good prognosis, pink denotes poor prognosis, and green indicates genes with unchanged expression.

Conversely, in the bad prognosis *WWOX*/*HIF1A* ratio, the interaction between *WWOX* and *HIF1A* could exacerbate the aggressiveness of gliomas by disrupting normal cellular processes such as cell cycle regulation (MMR, BER, HR) (*E2F4*, *SFN*, *CUL1*, *SKP2*, *TGFB2/3*, *ESCO2*), apoptosis (*BAK1*, *BAX*), and DNA replication (*DNA2*, *POLE/2*, *POLD1/3*, *PCNA*, *PRIM2*, *MCMs*, *RFC2/3/4*). Additionally, pathways like the PI3K-Akt signalling pathway, which is involved in cell survival and proliferation, could be affected by alterations in the *WWOX*/*HIF1A* axis. Other relevant pathways in cancer, such as focal adhesion, cytoskeleton regulation, and chemokine signalling (*VCL*, *THBS1-4*, *FLNA*, *LAMA2/4/5*, *HSPG2*), might also be influenced indirectly by changes in the *WWOX*/*HIF1A* ratio, potentially impacting the overall prognosis of low-grade glioma patients. Prognosis of low-grade glioma based on the *WWOX*/*HIF1A* ratio requires consideration of the role of immunological pathways that influence the tumour microenvironment (Figure 25) and disease progression, and immunological risk assessment allows for effective differentiation of patients and identification of those who may benefit from aggressive treatment, e.g., radiotherapy. Immune-related pathways such as TNF signalling (Figure 26) (*IDH3B/G*, *MDH1*, *ACO2*, *SDHA*, *OGDHL*) and JAK/STAT3 (PIAS2/3, PTPN2, AKT1/2, MCL1, CISH, AOX1) signalling are enriched in gliomas and play a critical role in their progression [72]. Additionally, the expression of checkpoint molecules like PD-L1 is associated with a worse prognosis and may indicate a potential target for immunotherapy.

To clarify the independent effect of these features, multivariate Cox proportional hazards regression was performed (*n* = 515, events = 92) (Supplementary Figure S7). In this model, patient age was the only significant independent predictor of survival (*HR* = 1.07 per year, 95% CI: 1.05–1.09, *p* = 1.3 × 10^−14^). The *WWOX*/*HIF1A* ratio showed a substantial estimated protective effect (HR = 0.54, 95% CI: 0.03–8.51), although this did not reach statistical significance (*p* = 0.66). Neither WWOX (*HR* ≈ 1, *p* = 0.52) nor HIF1A (*HR* ≈ 1, *p* = 0.77) expression individually predicted survival when adjusted for age and the ratio. The overall model discrimination was high (concordance index = 0.78).

These results indicate that while the *WWOX*/*HIF1A* ratio informs tumour biology and stratifies pathway activation in LGG, its independent prognostic value in multivariate survival analysis is less robust than that of clinical variables, such as age. Nonetheless, the molecular profile associated with a high ratio retains biological relevance and may serve as a component of integrated prognostic models or therapeutic targeting in future studies.

## 4. Discussion

The *WWOX*/*HIF1A* transcription ratio has emerged as a critical biomarker in cancer prognosis, offering valuable insights into tumour biology and informing therapeutic strategies. HIF-1α, a master regulator of cellular responses to hypoxia, modulates essential pathways such as angiogenesis, glycolysis, and autophagy [73]. Under normoxia, HIF-1α is hydroxylated by prolyl hydroxylases and degraded via the Von Hippel–Lindau (VHL) protein [74], but under hypoxia, it stabilizes to promote the Warburg effect by enhancing glycolysis and reducing oxidative phosphorylation, supporting tumour growth and survival [75]. Notably, dysregulation of HIF-1α is a hallmark of various cancers, including HCC, GBM, and breast cancer.

WWOX is one of the recognized regulators of HIF-1α activity. The WWOX protein directly interacts with HIF-1α via its WW domain, regulating both its stability and activity [76]. Through direct binding, WWOX sequesters HIF1A in the cytoplasm, thereby reducing its transcriptional activity. Additionally, WWOX modulates HIF1A-mediated signalling indirectly by associating with other transcription factors and signal transduction proteins, such as Dishevelled (DVL) proteins in the Wnt pathway and AP-2 transcription factors. Importantly, loss of WWOX amplifies HIF-1α-driven glycolysis and tumour progression, while also disrupting metabolic balance by impairing p53-mediated oxidative phosphorylation [15]. Beyond its metabolic roles, WWOX also influences the TME by modulating ECM remodelling and mesenchymal transition [77,78]. The tumour suppressor WWOX is frequently altered in numerous malignancies and is strongly associated with cancer cell differentiation and invasiveness.

In this discussion, we aim to integrate these findings, linking the differentially expressed genes to the broader context of the *WWOX*/*HIF1A* axis as a critical modulator of tumour aggressiveness and patient outcomes. By examining and evaluating the distinct prognostic associations across diverse malignancies and by highlighting the role of metabolic, ECM, and EMT pathways, we emphasize the potential of the *WWOX*/*HIF1A* ratio as a valuable biomarker for cancer prognosis.

### 4.1. Metabolic Reprogramming, EMT, Invasiveness, and Angiogenesis Across Tumour Types

Our studies have established that the *WWOX*/*HIF1A* ratio is a key regulatory axis in cancer biology, orchestrating cellular differentiation, metabolic reprogramming, invasiveness, EMT regulation, angiogenesis, and immune evasion in multiple tumour types. We observed that a low *WWOX*/*HIF1A* ratio promotes tumour progression by amplifying hypoxia-induced responses, whereas a high ratio contributes to microenvironmental stability and suppresses metastatic features. Our analyses confirm its subtype specificity, reflecting a complex molecular interaction that ultimately shapes clinical outcomes.

**Figure 27:**
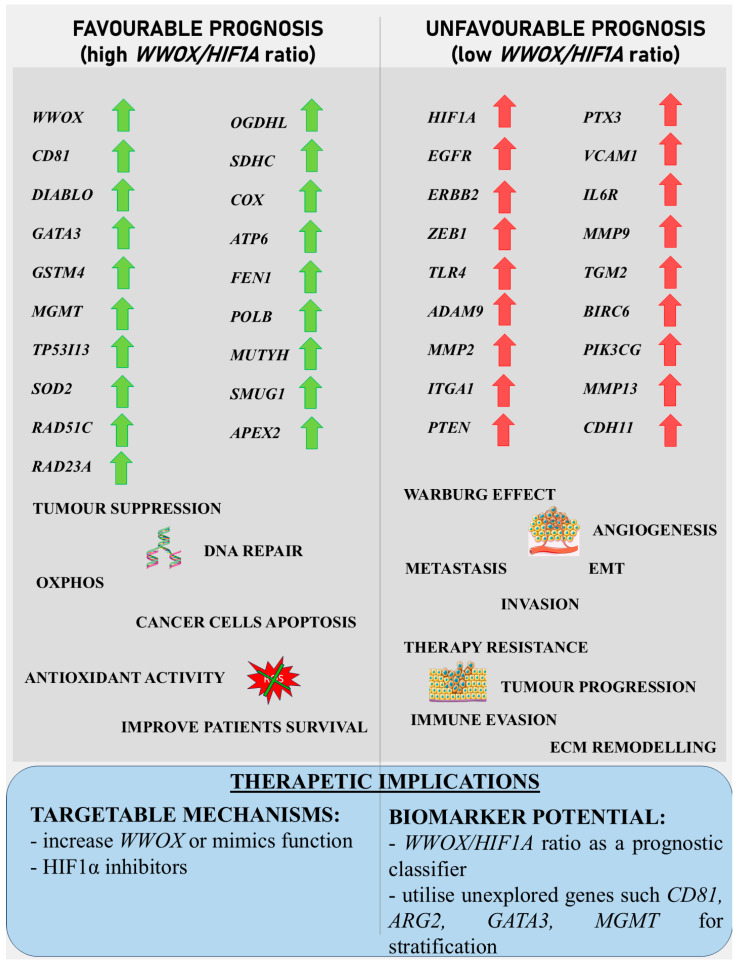
An illustration of the prognostic significance of the *WWOX*/*HIF1A* expression ratio in basal-like and HER2-positive breast cancer subtypes. Green color indicates the change in expression of genes associated with favorable prognosis, and red with unfavorable prognosis.

In basal-like breast cancer (Figure 27), we found that a low *WWOX*/*HIF1A* ratio is associated with particularly aggressive disease because it promotes EMT via ZEB1 and ADAM9, facilitates ECM remodelling via MMP2 and ITGA1, and enables immune evasion via NOTCH1 and TLR4. This molecular constellation highlights the hypoxia–invasion–inflammation nexus [17,79]. We further confirmed the role of HIF1A in the hypoxic tumour core, supporting its pro-metastatic role [17,80]. This is consistent with established hypoxia mechanisms, whereby HIF1A stabilization induces EMT via VEGF upregulation and E-cadherin repression [81,82]. On the other hand, we showed that higher WWOX expression promotes oxidative phosphorylation over glycolysis, thereby attenuating the Warburg effect and limiting tumour proliferation [9]. This metabolic change prevents the accumulation of tumour-promoting metabolites and aberrant protein synthesis [83] and aberrant protein synthesis [84]. Furthermore, we have linked a favourable prognosis in this context to further support the regulation of gene expression, such as DIABLO [85], GATA3 [86], GSTM4 [87], MGMT [88], TP53I13, and SOD2 [89], although GATA3 and MGMT remain underutilized as therapeutic targets [86,90].

A similar pattern is observed in HER2-positive breast cancers (Figure 27). We correlated a high *WWOX*/*HIF1A* ratio with improved prognosis through the activity of genes such as ARG2, S100A1, MT2A, EIF4EBP1, KLF4, BTG3, BAG1, and WWOX itself. EIF4EBP1 restricts oncogenic protein synthesis [91], MT2A mitigates oxidative stress [92], and ARG2 helps maintain metabolic balance [93]. Conversely, we identified that tumours with low ratios enrich inflammatory/ECM-disrupting genes, such as IL6R, VCAM1, and MMP13, which collectively drive immune suppression and metastasis, in accordance with a poor prognosis and increased invasiveness. Here we identified that MMP9, MMP13, VCAM1, and PTX3 overexpression not only promotes ECM remodelling and angiogenesis but also facilitates tumour infiltration, thereby reconfiguring the TME to favour migration and metastasis. Moreover, we observed that HIF1A upregulation enhances hypoxic adaptation and vascular remodelling, further compounding the invasive potential of these tumours [94]. Notably, VEGF—a key EMT inducer—synergizes with inflammatory cytokines such as IL-6 to accelerate invasion, thus creating an autocrine loop that perpetuates malignancy [81,95]. This further highlights the multifaceted nature of the *WWOX*/*HIF1A* axis in orchestrating both cellular and microenvironmental changes.

In contrast, in luminal A breast cancer subtype, our results reveal a more complex and, at times, paradoxical relationship with the *WWOX*/*HIF1A* ratio. We revealed context-dependent outcomes: favourable cases associate with metabolic flexibility and immune recruitment mediated by *PRKCB*, *HK3*, and cytokine signalling [96]. Poor prognosis cases, we found, upregulate *CYP4F2*, *FABP3*, and *MTHFR*, promoting immune evasion and metabolic dysfunction [97,98]. Notably, we uncovered paradoxical associations: PI3K-Akt signalling links to improved outcomes [99], while high *WWOX*/*HIF1A* ratios correlate with partial EMT via CDH2/DDR2 [12]—counteracted by *EMILIN1* upregulation, stabilizing ECM and inhibiting angiogenesis [100]. These ER+ tumours also demonstrate metabolic optimization through regulators such as *HK3*, *LDHAL6A*, *ADH6*, and *PRKCB*, which collectively shift metabolism toward greater efficiency [101]. Consequently, this constellation of gene expression supports better clinical outcomes [100,102]. This suggests that the prognostic impact of the *WWOX*/*HIF1A* axis is highly context-dependent and may be modulated by additional microenvironmental factors.

In luminal B breast cancers, we discovered a relationship that is paradoxical: high WWOX expression can correlate with worse outcomes, potentially due to overactive oxidative phosphorylation and excessive ROS production, or interference from oncogenic partners such as TRIM67, which promotes metastasis, ECM remodelling, and immune evasion via PI3K/Akt and MAPK pathway activation [95,103,104]. Here, elevated WWOX expression paradoxically increases invasiveness, most likely through dysregulation of ECM–receptor interactions and focal adhesion pathways [105]. In particular, altered ECM–receptor interactions are specifically implicated in poor prognosis. Nevertheless, we identified that favourable cases in this subtype are characterized by the expression of *FOXO3*, *DHFR*, *SIRT3*, and *RAD51*, which promote oxidative balance and enhance DNA repair.

**Figure 28:**
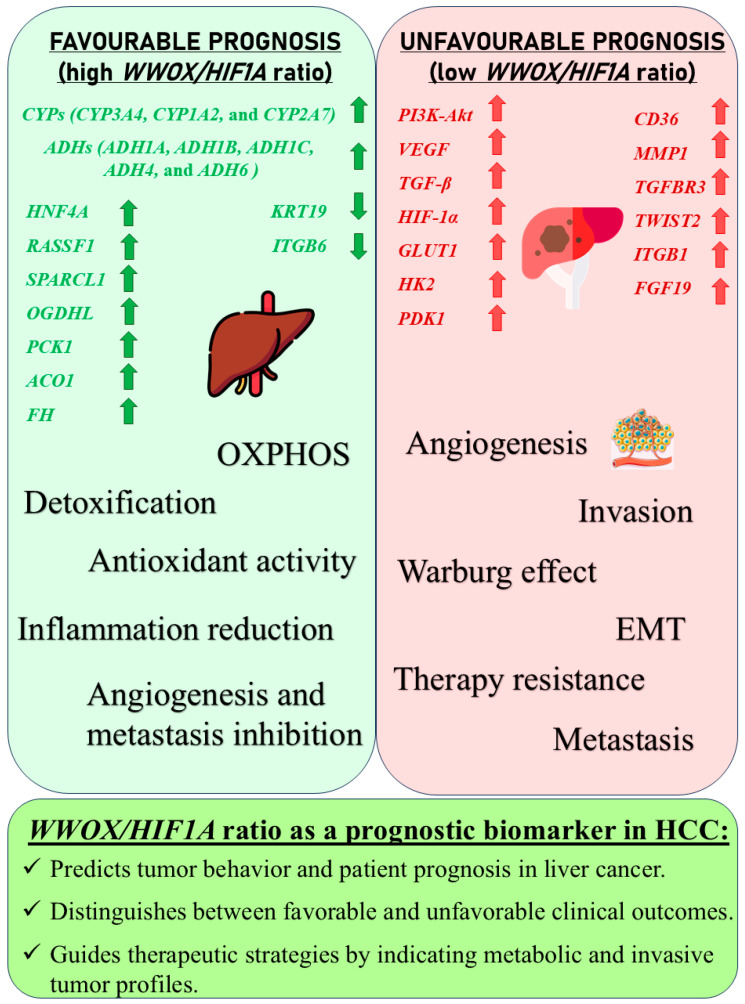
An illustration of the prognostic significance of the *WWOX*/*HIF1A* expression ratio in hepatocellular carcinoma. Green color indicates the change in expression of genes associated with favorable prognosis, and red with unfavor-able prognosis.

The impact of the *WWOX*/*HIF1A* ratio on EMT, invasiveness, and angiogenesis is not confined to breast cancer but extends to brain tumours and HCC as well. In case of HCC, we demonstrated that favourable HCC cases are characterized by high *WWOX*/*HIF1A* ratios that support oxidative metabolism and detoxification (Figure 28), including a 2.5-fold upregulation of *CYP3A4* [106] and induction of metallothionein genes such as *MT1G*, *MT1H*, *MT1F*, *MT1B*, and *MT1E* [107], which are crucial for antioxidant activity and metal homeostasis, in HCC [107,108,109]. They also play an essential role in inhibiting proliferation and invasion in HCC [107,108,109]. The good prognosis group shows higher expression of antioxidant and glutathione metabolism genes, helping limit hypoxia and inflammation that drive tumour progression [110]. So, high oxidative stress and low antioxidant capacity promote HCC [111], while higher antioxidant defences in patients with better prognosis limit tumour progression. Additionally, in cases with a poor prognosis, HIF1A-driven pathways, such as PI3K/AKT, TNF-α, and Wnt, promote tumour proliferation and survival by metabolic reprogramming, as well as upregulation of metastasis-promoting genes such as *MMP1*, *TWIST2*, and *ITGB1* [112]. Activation of PI3K/AKT increases glycolysis and supports tumour growth [113,114,115], and reduced expression of WWOX, by activating AKT, promotes tumour cell survival, proliferation, and treatment resistance [116]. The TNF-α pathway drives hepatocarcinogenesis through chronic inflammation [117] and the Warburg effect, mediated by NF-κB activation and increased expression of *GLUT1* and *HK2* [48]. Low *WWOX*/*HIF1A* ratios promote a microenvironment favouring EMT, invasiveness, and angiogenesis via HIF-1α-driven VEGF activation, leading to faster progression and poorer prognosis in our HCC subtype.

**Figure 29:**
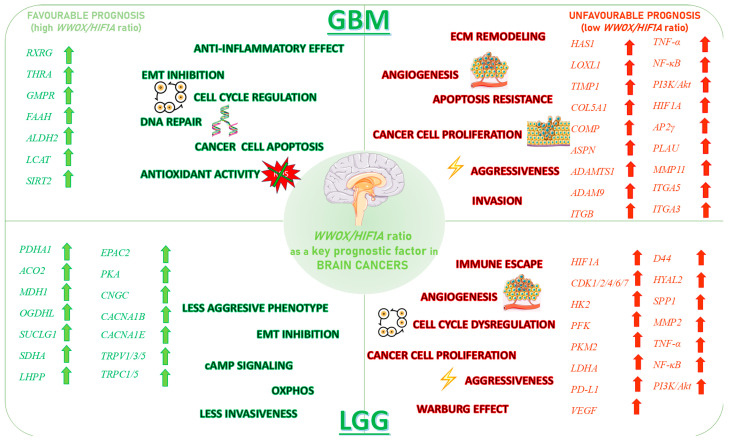
An illustration of the prognostic significance of the *WWOX*/*HIF1A* expression ratio in brain tumours. Green color indicates the change in expression of genes associated with favorable prognosis, and red with unfavorable prognosis.

We also showed that high *WWOX*/*HIF1A* ratios promote apoptosis and metabolic stability while suppressing EMT in brain tumours (GBM/LGG, Figure 29). Favourable cases, we noted, are marked by THRA-mediated metabolic regulation [118], RXRG-driven tumour suppression [119], GCGR-maintained metabolic homeostasis [120], and FAAH-mediated resolution of inflammation [121]. Our favourable prognosis LGG cases cohort with high *WWOX*/*HIF1A* ratios demonstrates enrichment of the oxytocin signalling pathway, which reduces TGF-β activity and thereby mitigates EMT and limits invasiveness [122,123]. These favourable cases are also characterized by upregulation of calcium signalling genes such as CACNA1B and CACNA1E, both of which are predictive of improved survival outcomes [124]. In contrast, gliomas with poor prognosis exhibit enhanced EMT, angiogenesis, and immune checkpoint expression, reinforcing an immunosuppressive and invasive phenotype. In these tumours, HIF1A upregulation drives pro-survival signalling cascades, including TNF, NF-κB, PI3K-Akt, and p53 dysregulation, which collectively facilitate proliferation and resistance to cell death. Concurrently, ECM-modifying genes such as *HAS1*, *LOXL1*, *TIMP1*, *MMP11*, *ADAMTS1*, *PLAU*, and integrins *ITGA5* and *ITGB1* are elevated, thereby contributing to structural remodelling and the creation of a permissive metastatic niche. Specifically, HAS1 synthesizes hyaluronic acid to support migration, while LOXL1 stiffens the ECM to enhance cell motility [122,125]. Similarly, in LGG, we found that a low *WWOX*/*HIF1A* ratio fosters invasive phenotypes through HIF-1α-induced VEGF signalling and overexpression of CXCR chemokine receptors (CXCR3/4/5/6), which collectively drive angiogenesis and tumour dissemination.

In summary, our findings establish that high *WWOX*/*HIF1A* ratios consistently promote oxidative metabolism, maintain differentiated cellular states, and suppress tumour proliferation across cancer types, whereas low ratios facilitate a metabolic shift toward glycolysis, promote EMT and dedifferentiation, and are associated with more aggressive tumour behaviour. This conserved pattern across multiple tumour types underscores the therapeutic potential of targeting the *WWOX*/*HIF1A* axis to reprogram tumour metabolism, restrict malignancy and disrupt metastatic progression, and improve clinical outcomes, although further research is warranted to fully elucidate the context-dependent effects observed across different tumour subtypes.

### 4.2. Cell Proliferation and Signalling Pathways Across Tumour Types

We found that the *WWOX*/*HIF1A* expression ratio critically regulates oncogenic signalling pathways and cell proliferation across diverse tumour types. Our analyses demonstrate that a consistently low ratio activates pro-survival signalling cascades and uncontrolled cell cycle progression, while a high ratio promotes genomic stability and balanced proliferation. Specifically, in basal-like breast cancer, we observed that low *WWOX*/*HIF1A* ratios correlate with upregulation of key proliferative pathways—including PI3K-Akt, MAPK, Ras, and ErbB signalling—which collectively drive tumour aggressiveness and rapid growth [126,127,128,129]. Additionally, our data reveal that oncogenes like *EGFR* and *ERBB2* further promote therapy resistance and proliferation, while *PIK3CA* activating mutations amplify PI3K-Akt signalling [126,130].

We observed a similar scenario in HER2-positive breast cancer, where our data confirm that low *WWOX*/*HIF1A* ratios intensify oncogenic signalling. Specifically, we identified that genes like BIRC6 and TGM2 enhance cell survival and migration under hypoxic conditions, while HIF1A promotes glycolytic metabolism and immune evasion, mechanisms we directly link to therapy resistance in our models. In the luminal A breast cancer subtype, our analyses reveal a nuanced relationship: PI3K-Akt signalling (particularly pAKT) paradoxically associates with improved prognosis, highlighting subtype-specific pathway roles [99,131,132]. However, we consistently observed that tumours with poor outcomes exhibit dysregulation in DNA replication, cell cycle control, and repair pathways, including p53 signalling axis aberrations driving malignancy [133].

In luminal B tumours, our analyses show genes such as *TP53I11* and *RAD51* correlate with better prognosis through enhanced apoptosis and homologous recombination repair (HRR), respectively [134,135]. Nonetheless, we found that *TRIM67* promotes aggressive phenotypes by amplifying PI3K/Akt and MAPK signalling, supporting proliferation and therapy resistance [104].

Additionally, we observed the influence of the *WWOX*/*HIF1A* axis in brain tumours. In GBM, our data indicate low *WWOX*/*HIF1A* ratios activate multiple proliferative and survival pathways regulated by *HIF1A* and its cofactors, including Wnt, TGF-β, AP2α, and AP2γ, collectively driving tumour progression and resistance to apoptosis [125,136]. We identified that key genes such as *CDK4* and *PTCH1* contribute to uncontrolled cell division and Hedgehog signalling, with targeted therapies against *EGFR* and *CDK4/6* showing promising efficacy [17,137,138,139]. In LGG, low ratios hyperactivate cyclin-dependent kinases (*CDK1/2/4/6/7*), inducing genomic instability [140]. Conversely, we observed that favourable cases exhibit enriched cAMP and calcium signalling, where *EPAC2* reduces invasiveness and *CACNA1B/E* predict improved survival [124,141]. In this regard, our data show that HCC subtype follows this pattern, as low *WWOX*/*HIF1A* ratios activate PI3K/AKT, TNF-α, and Wnt pathways, driving proliferation and therapy resistance [113]. We found that *WWOX* deficiency specifically promotes AKT-mediated survival, while *FGF19* enhances proliferation [142]. On the other hand, our analyses identified tumour suppressors like *LHPP* that inhibit growth, and *CYP3A4* emerges as a favourable HCC prognostic marker [106,143].

Collectively, our findings demonstrate that the *WWOX*/*HIF1A* ratio critically modulates oncogenic signalling across multiple tumour types, influencing prognosis, therapeutic response, and disease progression through the regulation of key proliferation and survival pathways.

### 4.3. Genomic Integrity and DNA Repair Across Tumour Types

In addition to its established roles in metabolism and signalling, our analyses identified through our analyses that the *WWOX*/*HIF1A* expression ratio emerges as a pivotal determinant of genomic integrity and DNA repair fidelity across multiple cancer types. We consistently associated high *WWOX*/*HIF1A* ratios with enhanced DNA repair capacity, as evidenced by the upregulation of key genes such as *FOXO3*, *DHFR*, *RAD51*, and *TP53I13*, which collectively counteract replication stress and oxidative damage [135,144]. This genomic safeguarding is particularly evident in basal-like breast cancer, where we demonstrated that elevated *WWOX* expression not only supports detoxification and nucleotide metabolism but also reinforces DNA repair pathways. For instance, *MGMT* upregulation contributes to the reversal of chemotherapy resistance by enhancing direct repair mechanisms [12,88], while *TP53I13* and *SOD2* further promote genomic stability and resilience to oxidative stress [89]. Conversely, we linked low *WWOX*/*HIF1A* ratio with genomic instability, unchecked cyclin-dependent kinase (CDK) activity, and impaired DNA repair—phenomena that are particularly pronounced in LGG and GBM [140]. In these brain tumours, loss of WWOX expression removes repression of pro-invasive genes such as *MMP1*, thereby facilitating *HIF1A*-driven ECM remodelling, which exacerbates tumour aggressiveness and further undermines genomic stability.

We observed a similar dichotomy in HER2-positive breast cancers, where a favourable *WWOX*/*HIF1A* ratio facilitates robust DNA repair and counteracts hypoxia-induced signalling, which would otherwise promote angiogenesis and glycolytic reprogramming linked to poor prognosis. In contrast, tumours with low ratios are more susceptible to genomic instability and the downstream consequences of impaired repair. In luminal A breast cancers, we associated high *WWOX*/*HIF1A* ratios are associated with the maintenance of DNA repair and genomic integrity, whereas poor prognosis cases are marked by significant *WWOX* downregulation and elevated *HIF1A*, resulting in dysregulated DNA replication and repair pathways that drive unchecked proliferation [12,133]. In luminal B breast cancer, we documented that genes including *FOXO3* [144], *DHFR* [145], *RAD51* [135], and *DHX9* [146] support genomic maintenance by regulating apoptosis and DNA repair mechanisms; however, the protective effects of *WWOX* in this context can be compromised by dominant-negative truncated protein forms [13], potentially undermining these processes.

The situation in HCC further underscores the importance of the *WWOX*/*HIF1A* axis in genomic maintenance. We established that tumours with low *WWOX*/*HIF1A* ratios exhibit elevated ROS levels and hypoxia, which together impair DNA repair and promote genomic instability. Downregulation of metallothionein genes such as *MT1G* and *MT1H* correlates with poor outcomes by reducing apoptosis, weakening p53 pathway control, and enabling Wnt/β-catenin activation [107,108,109]. By contrast, we observed favourable prognosis groups to demonstrate upregulation of glutathione metabolism pathways that buffer ROS, protect against DNA damage, and inhibit invasion [110,111].

Taken together, our observations highlight the *WWOX*/*HIF1A* axis as a central regulator of genomic stability, integrating metabolic control and DNA repair pathway selection. Its disruption not only creates vulnerabilities that drive tumour progression and therapeutic resistance but also points to new opportunities for targeted interventions aimed at restoring genomic integrity and improving long-term patient outcomes across diverse cancer types.

### 4.4. Immune Regulation and Inflammation Across Tumour Types

We established that the *WWOX*/*HIF1A* ratio orchestrates tumour immune responses, fundamentally shaping immune evasion, inflammation, and immune cell infiltration across cancers. Our mechanistic analyses revealed that WWOX loss stabilizes HIF1α, driving transcriptional reprogramming of metabolic and immune pathways—linking metabolic adaptation to immune modulation [80]. In basal-like and HER2-positive breast cancers, we demonstrated that a low *WWOX*/*HIF1A* ratio is particularly detrimental, as it triggers HIF1α-mediated upregulation of immunosuppressive mediators such as *TLR4*, *NOTCH1*, *PTX3*, and *IL6R*, alongside glycolytic enzymes like *GLUT1* and *HK2* [147,148,149]. This creates an inflammatory microenvironment that blocks immune surveillance and promotes metastasis. We further observed that *VCAM1* overexpression consolidates metastasis-permissive niches by impeding T-cell infiltration [96,150].

For luminal breast cancers, we found that high *WWOX*/*HIF1A* ratios demonstrate a more robust anti-tumour immune response, as evidenced by increased expression of *CD8A* and *FOXP3*, which are instrumental in recruiting cytotoxic T-cells and suppressing pro-tumourigenic inflammation [151,152]. However, we identified an exception in poor-prognosis luminal A tumours, where *CYP4F2* overexpression metabolically suppresses T-cell function—undermining immune surveillance even with favourable ratios. In poor-prognosis luminal A tumours, for example, overexpression of *CYP4F2* can metabolically suppress T-cell function, thereby undermining immune surveillance and facilitating immune evasion even in the presence of a favourable *WWOX*/*HIF1A* ratio.

In gliomas, we documented brain-specific immunomodulatory impact of the *WWOX*/*HIF1A* axis, which is also context-dependent. In GBM, high *WWOX*/*HIF1A* conditions leverage *FAAH*-mediated lipid signalling to resolve inflammation and induce tumour cell death, providing a potential mechanism for immune-mediated tumour suppression [121,153]. Conversely, in LGG, a low *WWOX*/*HIF1A* ratio is associated with increased expression of immune checkpoint molecules such as PD-L1 and pro-inflammatory cytokines like TNF-α. This, in turn, activates NF-κB and PI3K survival pathways, fostering an immunosuppressive niche that supports tumour persistence [154,155,156,157]. Notably, we also linked co-expression of *CD44*, *HYAL2*, *SPP1*, and *MMP2* functionally links ECM remodelling to T-cell exclusion, further exacerbating immune evasion [158].

In HCC we uncovered a self-sustaining immunosuppression cycle: alcohol-induced HIF1α [159] stabilization and TNF-α-driven [48] lactate production converge with WWOX deficiency to promote GLUT1 overexpression and T-cell exhaustion markers. The convergence of WWOX deficiency, GLUT1 overexpression, and markers of T-cell exhaustion highlights a critical vulnerability in HCC, suggesting that therapeutic strategies targeting glycolytic-immune crosstalk may hold significant promise.

Collectively, our findings position the *WWOX*/*HIF1A* axis as a master regulator of tumour-immune dynamics, whose disruption drives immune escape through context-dependent mechanisms. This highlights its therapeutic potential for restoring anti-tumour immunity across cancers.

### 4.5. Underexplored Genes and Clinical Potential Across Tumour Types

Our comprehensive analyses across multiple tumour types identified a wealth of clinically relevant genes that remain underutilized in current therapeutic strategies Table 1, presenting significant opportunities for advancing cancer treatment. While the *WWOX*/*HIF1A* ratio effectively distinguishes molecular subgroups and correlates with clinical outcomes across the cancers studied, its independent prognostic value in multivariate models did not reach statistical significance in most cohorts, likely due to sample size constraints and the overriding influence of established clinical predictors such as age and stage. These findings warrant external validation in larger, prospective datasets.

Within basal-like breast cancer, we identified several potential therapeutic targets not yet exploited clinically. *CD81* is overexpressed and linked to poor prognosis, yet it remains untapped in treatment paradigms [160]. The tumour suppressor GATA3, critical for differentiation, proliferation, and apoptosis, is similarly underutilized in basal-like therapies [86]. Our findings also highlight MGMT’s limited role in aggressive basal-like, ER-negative cases, where promoter methylation [90] and BRCA1 dysfunction reduce its expression [161]. Additionally, we identified DIABLO/Smac as a potential therapy enhancer that merits further study [162]. While *WWOX*/*HIF1A* ratio-based gene and pathway patterns stratify risk effectively, multivariate analysis (Supplementary Figure S1) shows that clinical stage and WWOX expression—but not the ratio itself—are independent prognostic factors, underscoring the need to integrate molecular and clinical data for risk assessment.

In HER2-positive disease, our findings associate genes such as *ARG2*, *S100A1*, *MT2A*, *EIF4EBP1*, *KLF4*, *BTG3*, and *BAG1* to be associated with favourable prognosis through their roles in metabolic regulation, oxidative stress mitigation, and tumour suppression [91,92,93]. However, these genes are not currently targeted by standard HER2-directed therapies such as trastuzumab or kinase inhibitors. There is also potential in targeting hypoxia-related pathways (e.g., HIF1A inhibition), inflammatory mediators (e.g., *IL6R*), or enhancing WWOX expression may improve outcomes in high-risk patients with unfavourable molecular profiles. Moreover, HER2-positive cancers showed that a high *WWOX*/*HIF1A* ratio is associated with improved molecular features and clinical outcomes, but multivariate survival modeling found age and stage to be the only significant independent predictors, suggesting the need for larger studies to clarify the standalone effect of the ratio.

For luminal A breast cancer, we observed that genes associated with favourable prognosis—including key regulators of glucose metabolism *HK3*, *LDHAL6A*, apoptosis regulator *PRKCB*, and alcohol dehydrogenase *ADH6*—are not directly targeted by current treatments. Conversely, genes linked to poor outcomes, such as *MYH7*, *FABP3*, *CYP4F2,* and *MTHFR*, are largely absent from therapeutic regimens despite their impact on hypoxia adaptation, immune modulation, and genomic stability. Integrating these markers into personalized medicine approaches could substantially enhance therapeutic efficacy [98,163,164,165,166]. Uniquely, a higher *WWOX*/*HIF1A* ratio in luminal A was independently linked to poorer prognosis in multivariate analysis, setting it apart from other subtypes and highlighting a complex, potentially paradoxical, interaction between metabolic pathways and disease outcome.

In luminal B breast cancer, our data show that both *WWOX* and *TRIM67* remain underutilized in clinical practice. Although *WWOX* is recognized as a tumour suppressor, our analyses reveal a paradoxical association with poorer outcomes in high *WWOX*/*HIF1A* ratio groups, necessitating deeper mechanistic exploration. We also identified *TRIM67* as a promising prognostic and therapeutic candidate, implicated in metastasis and therapy resistance, yet not clinically implemented. While established treatments for luminal B include hormone therapy, targeted agents, and chemotherapy [167], our results highlight genes such as *FOXO3*, *DHFR*, *CD8A*, *ESR2*, *TP53I11*, *DHX9*, *RAD51*, and *FOXP3* that offer additional promise for future targeted therapies. Notably, we found *FOXO3* to be the most frequently mutated gene among those tested, with significant implications for tumour biology [168]. Our analysis also shows that high *CD8A* expression is linked to an elevated TME score and poorer prognosis. The luminal B tumours exhibit the highest ESR1 and lowest ESR2 expression among all subtypes [169], with ESR2 being particularly scarce—a feature that distinguishes luminal B from ERα-negative cancers, where ESR2 may have clinical relevance [170]. Nevertheless, only ERα (*ESR1*) expression is currently assessed in routine clinical practice [171]. Additionally, our data indicate that *TP53I11* expression in primary tumours correlates with overall survival, while high *RAD51* expression is linked to worse outcomes due to its role in HR and genomic instability [172,173]. The complex, paradoxical relationship between the *WWOX*/*HIF1A* ratio and prognosis in Luminal B—similar to Luminal A—highlights the need for further study of subtype-specific metabolic and signalling interactions. Although a high ratio and related gene signatures associate with poor outcomes, only tumour stage and select gene expressions like *TP53I11* are independently predictive, indicating that the *WWOX*/*HIF1A* axis functions within a broader context of subtype-specific risk factors.

In GBM, our analyses demonstrate that the *WWOX*/*HIF1A* axis presents a viable therapeutic target. We propose that restoration of *WWOX* or inhibition of *HIF1A* could recalibrate pathways regulating apoptosis, EMT, and metabolism. Our findings suggest that targeting extracellular matrix components, inflammatory signalling, and metabolic vulnerabilities may further improve outcomes in this heterogeneous cancer. We identified key genes such as *MMP1*, *PTCH1*, *CDK4*, and *CDKN1B* as associated with GBM prognosis and functionally relevant to the *WWOX*/*HIF1A* ratio. For example, our data indicate that WWOX loss disinhibits *MMP1*, facilitating ECM degradation driven by *HIF1A* upregulation [174]; *PTCH1* may be aberrantly activated with a decreased *WWOX*/*HIF1A* ratio [17]; and *CDK4* activation drives unchecked proliferation [137]. We note that *EGFR* and *CDK4/6* inhibitors are currently under clinical investigation for GBM [138,139]. In GBM, elevated WWOX and reduced HIF1A expression mark better prognosis, with clear differences in signalling and metabolic pathways between subgroups. While these molecular signatures distinguish tumour aggressiveness, only age and certain cell cycle genes (*CDK4*, *CDKN1B*) achieved independent prognostic significance in multivariate models, underscoring the need for further validation of molecular risk markers.

For LGGs, our results suggest that improved prognostic markers beyond traditional histopathology are needed. While IDH1/2 mutations, TP53 status, and 1p/19q codeletion are established determinants [175], our analyses highlight additional genes such as *WWOX*, *HDAC11*, *BIN1*, *PARK2*, *BCL2L2*, *SOD1*, and *APOE* as potentially valuable in relation to the *WWOX*/*HIF1A* ratio. We found that WWOX downregulation and a low *WWOX*/*HIF1A* ratio are linked to adverse outcomes, while *HDAC11* overexpression is associated with therapy resistance [176]. Early evidence from our studies supports roles for BIN1, *PARK2*, and *BCL2L2* in LGG progression and treatment response [177,178,179], although further validation is needed [180,181]. The prognostic significance of SOD1 and APOE remains to be clarified. We propose that incorporating these genes into multi-gene prognostic models could enhance risk stratification and inform treatment decisions for LGG patients. In LGG, a higher *WWOX*/*HIF1A* ratio is associated with favourable molecular profiles—upregulation of WWOX, maintenance of epithelial integrity, downregulation of mesenchymal transition, and metabolic adaptation—all corresponding to improved prognosis. However, in multivariate analysis, this ratio was not an independent predictor of survival when accounting for clinical variables such as age, which remained the strongest prognostic factor.

In HCC, our data link several genes to prognosis through their roles in tumour progression or suppression, yet none are currently standard therapeutic targets. We identified *CD36*, *MMP1*, *TGFBR3*, *TWIST2*, *ITGB1*, and *FGF19* as associated with poorer outcomes: *CD36* enhances tumour stemness and growth [182], *MMP1* facilitates metastasis [183], *TGFBR3* and *TWIST2* promote EMT and invasion [184], *ITGB1* supports cell adhesion and survival [185], and FGF19 drives proliferation [142]. In contrast, *HNF4A*, *RASSF1*, *SPARCL1*, and *LHPP* are associated with favourable outcomes: *HNF4A* regulates hepatocyte differentiation [186], *RASSF1* acts as a tumour suppressor [187], SPARCL1 inhibits angiogenesis and metastasis [188], and *LHPP* suppresses tumour growth [143]. While these genes are not yet part of standard HCC therapies, some are being explored as therapeutic targets. For example, *FGF19* is implicated in the *FGFR4* pathway, which is targeted by inhibitors such as fisogatinib [189]. *MMP1*, *TGFBR3,* and *ITGB1*, given their roles in metastasis and EMT, are also attractive candidates for targeted inhibition. Restoring tumour suppressors such as *HNF4A* and *RASSF1* could further improve outcomes, though this remains an area of active research. A high *WWOX*/*HIF1A* ratio in HCC defines a subgroup with enhanced metabolic detoxification, efficient oxidative phosphorylation, and reduced tumour invasion. Despite these clear biological distinctions, the ratio was not a statistically significant independent predictor in multivariate analysis, with age remaining the principal prognostic factor.

## 5. Conclusions

In summary, the hypoxia inducible factor HIF1α is undoubtedly a key regulator of metabolic reprogramming in cancer cells, enabling their sustained and uninhibited growth, particularly under hypoxic conditions. By activating the transcription of genes involved in glycolysis, angiogenesis, and cell survival, *HIF1A* orchestrates the adaptation of cancer cell metabolism to low oxygen environments, thereby promoting tumour progression and resistance to therapy. The expression and activity of *HIF1A* are tightly regulated at multiple levels, ensuring precise control over its function in both physiological and pathological conditions. Oncogenic signalling pathways, such as PI3K/AKT/mTOR and RAS/RAF/MEK/ERK, JAK/STAT, Wnt/β-catenin, Notch, and NF-κB, significantly enhance the transcription of HIF1A, leading to increased protein levels even under normal oxygen conditions. This upregulation is often observed in cancer cells, where these pathways are frequently activated, contributing to cancer progression.

However, our attention was focused on the tumour suppressor *WWOX*, which is thought to play an important role as a negative regulator of *HIF1A*. Interestingly, the *WWOX*/*HIF1A* ratio appears to be associated with prognosis in patients with various subtypes of breast cancer, brain tumours, and HCC, suggesting it may impact important biological processes in these cancers.

The *WWOX*/*HIF1A* axis emerges as a central regulator of tumour metabolism and progression across diverse malignancies. Our extensive analysis reinforces the tumour suppressor WWOX as a key modulator of cancer metabolism through direct physical interaction with HIF1α via its WW domain, inhibiting its transactivation potential and promoting its degradation. This regulation prevents the Warburg effect—a metabolic shift toward aerobic glycolysis—and maintains oxidative phosphorylation.

The *WWOX*/*HIF1A* expression ratio serves as a robust prognostic biomarker, reflecting the balance between tumour-suppressive and oncogenic pathways. A high ratio correlates with favourable prognosis by promoting oxidative metabolism, DNA repair, and immune surveillance, while a low ratio drives poor outcomes via glycolysis, angiogenesis, and EMT. Critically, WWOX loss triggers HIF1α stabilization, inducing metabolic rewiring that fuels tumour growth and aggressiveness. This axis also influences systemic metabolic homeostasis, with dysregulation predisposing to metabolic disorders.

Our findings highlight that a high *WWOX*/*HIF1A* ratio correlates with favourable prognosis across multiple cancers by promoting oxidative metabolism, enhancing DNA repair, maintaining ECM integrity, and supporting immune surveillance. Conversely, a low ratio is linked to poor outcomes characterized by enhanced glycolysis, angiogenesis, EMT, immune evasion, and activation of oncogenic signalling pathways such as PI3K/Akt, MAPK, and Wnt. These molecular alterations are evident in breast cancer subtypes (basal, HER2-positive, luminal A and B), HCC, GBM, and LGG, underscoring the universal relevance of the *WWOX*/*HIF1A* axis in tumour biology.

Importantly, the *WWOX*/*HIF1A* ratio not only reflects tumour metabolic states but also integrates signals from the TME, including ECM remodelling and immune responses, which further influence tumour progression and therapeutic resistance. The axis’s modulation affects key processes such as apoptosis, cell cycle control, hypoxia adaptation, and inflammatory signalling, making it a promising target for therapeutic intervention.

Given the central role of WWOX in restraining HIF1α-driven metabolic reprogramming, therapeutic strategies aimed at restoring WWOX function or inhibiting HIF1α activity hold significant potential to disrupt cancer metabolism and improve patient outcomes. Future research should focus on elucidating the precise molecular mechanisms governing WWOX-HIF1α interactions, exploring their clinical utility as biomarkers, and developing targeted therapies that exploit this axis to overcome tumour aggressiveness and resistance.

A limitation of our study is that we rely solely on bulk RNA-seq data, resulting in the loss of cellular resolution, as this approach provides an average expression profile across all cells in a sample. This could obscure important cancer cell heterogeneity, masking rare subpopulations and making it difficult to attribute gene expression changes to specific cell types, which can reduce the reliability and replicability of our findings. Nevertheless, these results may constitute a starting point for further studies on the significance of the *WWOX*/*HIF1A* ratio in various types of cancer.

## Figures and Tables

**Table 1:** This table summarizes key gene and pathway findings related to the *WWOX*/*HIF1A* prognostic ratio and their clinical relevance in tumourigenesis across GBM, LGG, HCC, and BRCA. It also lists genes identified in this study as contributing to prognosis according to the *WWOX*/*HIF1A* ratio, offering an overview of their potential as biomarkers and therapeutic targets in these malignancies. Arrows indicate gene expression: ↑ denotes higher expression, ↓ denotes lower expression in each cancer subtype. Basal BC—Basal-like Breast Cancer; HER2 BC—HER2-positive Breast Cancer; Luminal A BC—Luminal A Breast Cancer; Luminal B BC—Luminal B Breast Cancer; GBM—Glioblastoma Multiforme; LGG—Low Grade Glioma; HCC—Hepatocellular Carcinoma.

Clinical Relevance		Basal BC	HER2 BC	Luminal A BC	Luminal B BC	GBM	LGG	HCC
***WWOX*/*HIF1A* prognostic Ratio**		higher associated with good prognosis	higher associated with good prognosis	lower associated with good prognosis	lower associated with good prognosis	higher associated with good prognosis	higher associated with good prognosis	higher associated with good prognosis
**Pathway Specificity (Good Prognosis)**		Lipid and Inflammatory Pathways, DNA Repair and Stability, Central Metabolism, Reactive Oxygen Species (ROS) and Damage, Energy Production	DNA Replication and Repair, Cell Cycle Regulation, Metabolism, Protein Synthesis, Reactive Oxygen Species, Lipid and Sugar Metabolism	Growth and Proliferation, Second Messenger Systems, Immune and Inflammatory Response, Cell Adhesion and Cytoskeleton Dynamics, Cancer-Specific Pathways, Infection-Related Pathways	Immune System and Inflammation, Cell Cycle and DNA Repair, Signal Transduction, Cancer-Specific Pathways, Neuroactive and Hormonal Signalling, Cell Adhesion and Cytoskeleton Dynamics, Metabolism and Biosynthesis, Gap Junctions and Vascular Function	Signal Transduction Metabolism, Cellular Processes, Stem Cell Regulation, Carbohydrate Metabolism	Energy Production and Metabolism, Amino Acid and Nitrogen Metabolism, Signal Transduction, Hormonal Regulation, Lipid and Arachidonic Acid Metabolism, Cellular Dynamics and Motor Proteins, Biosynthesis and Cofactors, Neuroactive Processes	Energy Production and Metabolism, Lipid and Fatty Acid Metabolism, Amino Acid Metabolism, Nitrogen and Sulphur Metabolism, Carbohydrate Metabolism, Vitamin and Cofactor Metabolism, Drug and Xenobiotic Metabolism, Cellular functions
**Pathway Specificity (Poor Prognosis)**		Growth and Proliferation, ECM and Cell Adhesion, Hypoxia and Metabolism, Immune and Inflammatory, Apoptosis and Senescence	Hormonal and Reproductive Signalling, Cytoskeleton and Cell Adhesion, Immune and Inflammatory Signalling, Cancer and Cellular Processes, Stem Cell and Longevity Regulation, Metabolism and Biosynthesis, Vascular and Muscle Function, Genetic Information Processing, Diabetes and Endocrine Disorders	DNA Replication and Repair, Cell Cycle Regulation, Reactive Oxygen Species (ROS) and Damage, Drug Metabolism, Nitrogen and Carbon Metabolism, Nucleotide and Cofactor Biosynthesis, Protein and Motor Functions, Hormonal Regulation, Histidine Metabolism, Endocannabinoid Signalling	DNA Replication and Repair, Cell Cycle and Senescence, Reactive Oxygen Species (ROS) and Damage, Drug Metabolism, Metabolism and Biosynthesis, Hormonal Regulation, Immune and Cellular Processes, Neuroactive and Cellular Signalling, Energy Production and Intermediates, Motor Proteins and Cellular Dynamics	Immune and Inflammatory Response, Cancer-Specific Pathways, Signal Transduction, Cell Adhesion and Cytoskeleton Dynamics, Cell Cycle and Apoptosis, Metabolism and Biosynthesis, Endocytosis and Intracellular Transport, Hormonal and Reproductive Signalling, Diabetes and Complications, Neuroactive Processes	Cell Adhesion and Extracellular Matrix, Immune and Inflammatory Response, DNA Replication and Repair, Cell Cycle and Apoptosis, Signal Transduction, Cancer-Specific Pathways, Metabolism and Biosynthesis, Diabetes and Complications	Signal Transduction, Immune and Inflammatory Response, Cancer-Specific Pathways, Cell Adhesion and Cytoskeleton Dynamics, Cell Cycle and Apoptosis, Metabolism and Biosynthesis, Neuroactive Processes, Pathogen Interaction and Resistance, Diabetes and Complications, Chromatin and Gene Regulation
**Therapeutic/Clinical Relevance**	**Markers with Direct Therapeutic Application**	*ERBB2*, *EGFR*, *PTEN*, *HIF1A*, *IL1RAPL1*, *NOTCH1*	*EGFR*, *PTEN*, *HER2*	*ESR1,PGR*, *FGFR2*	*PIK3CA*	*PTEN*	NA	*VEGF-D*, *FGF19*
	**Markers with Potential Therapeutic Application**	*CD44*, *BCAR4*, *PIK3CA*, *MMP2*, *PARP*, *MMP9*, *VEGF*	*MMP15*, *ADCYAP1*, *HIF1A*, *LYN*, *PRKG1*, *STK39*, *PLXNC1*	*CDK6*, *MMP10*, *PIK3CG*, *GPER*, *LYN*, *WWOX*, *ID01*	*KCNC1*	*ADAMTS1*, *CD44*, *IL6*, *NRP1*, *HIF1A*, *CD70*, *LYN*	*LYN*, *BRAF*	*BCL2L15*, *HNF4A*, *MMP1*, *TGFBR3*
**Functional Role in Tumourigenesis**	**Markers Promoting Tumour Growth**	*HIF1A*, *EGFR*, *ERBB2*, *PIK3CA*, *PTEN*, *MYCBP2*, *ITGA1*, *MMP2*, *ZEB1*, *ERBB2*, *TLR4*, *CCNE2*	*EGFR*, *PTTG1*, *ERBB2*, *MMP15*, *ADCYAP1*, *HIF1A*, *CDC6*	*ERBB2*, *CCND1*, *ADCYAP1*	*AURKA*, *CCNE2*, *HIF1A*, *MYCBP2*, *NRC1*	*HIF1A*, *EGFR*, *PIK3CD*, *AKT1*, *PTK7*, *MKNK2*, *RELB*, *S100A8*, *PTGS2*, *COL1A1*, *CD44*, *AXL*, *PLAU*	*IDH1*, *EGFR*, *CDK4/6*, *FGFR*	*FGF19*, *MYCL1*, *CD36*
	**Markers Promoting Tumour Suppression**	*CD81*, *GATA3*, *MGMT*, *DIABLO*, *SOD2*, *TP53I13*, *BARD1*, *PTEN*	*PTEN*, *RB1*	*CDH1*, *WWOX*, *CILP*, *TP53*	*FAS*, *CDH1*	*PTEN*	*TP53*	*HNF4A*, *RASSF1*
	**Markers Involved in Metastasis**	*ITGA1*, *MMP2*, *ZEB1*, *CD44*	*CD44*, *PLXNC1*, *ITGA4*	*CD44*, *MMP10*, *FAT1*, *ITGA4*	*KLK13*, *ITGA1*	*SPP1*, *MMP13*, *LOXL1*, *MMP14*, *ITGA4*	*MMP2*, *VEGF*	*TWIST2*, *MMP1*, *CDH6*, *TGFBR3*
**Identified genes from this study contributing to prognosis according to *WWOX*/*HIF1A* ratio**		↑ *CD81*, ↑ *GATA3*, ↑ *MGMT*	↑ *ARG2*, ↑ *S100A1*, ↑ *MT2A*, ↑ *EIF4EBP1*, ↑ *KLF4*, ↑ *BTG3*, ↑ *BAG1*	↑ *HK3*, ↑ *LDHAL6A*, ↑ *ADH6*, ↑ *PRKCB*, ↓ *MYH7*, ↓ *FABP3*, ↓ *CYP4F2*, ↓ *MTHFR*	↓ *WWOX*, ↓ *TRIM67*, ↑ *FOXO3*, ↑ *DHFR*, ↑ *CD8A*, ↑ *ESR2*, ↑ *TP53I11*, ↑ *DHX9*, ↑ *RAD51*, ↑ *FOXP3*	↓ *MMP1*, ↑ *PTCH1*, ↑ *CDK4*, ↑ *CDKN1B*	↑ *WWOX*, ↑ *HDAC11*, ↑ *BIN1*, ↑ *BCL2L2*, ↑ *PARK2*, ↑ *SOD1*, ↑ *APOE*	↓ *MMP1*, ↑ *CD36*, ↑ *TWIST2*, ↑ *FGF19*, ↑ *TGFBR3*, ↓ *ITGB1*, ↑ *HNF4A*, ↓ *RASSF1*, ↑ *SPARCL1*, ↑ *LHPP*

## Data Availability

TCGA RNA-seq data for breast cancer (BRCA), glioblastoma (GBM), low-grade glioma (LGG), and hepatocellular carcinoma (HCC) are publicly available from (http://gdac.broadinstitute.org/ data status of 28 January 2016). Gene sets used are available in Supplementary File S1.

## Supplementary Materials

The following supporting information can be downloaded at: https://www.mdpi.com/article/10.3390/biology14091151/s1, Supplementary Table S1: TCGA patient cohort clinical information; Supplementary Table S2: Genes involved in major cancer-related pathways and their expression in Basal breast cancer subtype in relation to prognostic *WWOX*/*HIF1A* ratio; Supplementary Table S3: Genes involved in major cancer-related pathways and their expression in HER2 breast cancer subtype in relation to prognostic *WWOX*/*HIF1A* ratio; Supplementary Table S4: Genes involved in major cancer-related pathways and their expression in luminal A breast cancer subtype in relation to prognostic *WWOX*/*HIF1A* ratio; Supplementary Table S5: Genes involved in major cancer-related pathways and their expression in luminal B breast cancer subtype in relation to prognostic *WWOX*/*HIF1A* ratio; Supplementary Table S6: Genes involved in major cancer-related pathways and their expression in hepatocellular carcinoma subtype in relation to prognostic *WWOX*/*HIF1A* ratio; Supplementary Table S7: Genes involved in major cancer-related pathways and their expression in glioblastoma in relation to prognostic *WWOX*/*HIF1A* ratio; Supplementary Table S8: Genes involved in major cancer-related pathways and their expression in low-grade glioma in relation to prognostic *WWOX*/*HIF1A* ratio; Supplementary Figure S1: Multivariate Cox Regression analysis of prognostic factors in basal breast cancer. Forest plot visualizing hazard ratios, confidence intervals, and significance for the *WWOX*/*HIF1A* ratio, gene expressions, age, and stage; Supplementary Figure S2: Multivariate Cox Regression analysis of prognostic factors in Her2 breast cancer. Forest plot visualizing hazard ratios, confidence intervals, and significance for the *WWOX*/*HIF1A* ratio, gene expressions, age, and stage; Supplementary Figure S3: Multivariate Cox Regression analysis of prognostic factors in Luminal A breast cancer subtype. Forest plot visualizing hazard ratios, confidence intervals, and significance for the *WWOX*/*HIF1A* ratio, gene expressions, age, and stage; Supplementary Figure S4: Multivariate Cox Regression analysis of prognostic factors in Luminal B breast cancer subtype. Forest plot visualizing hazard ratios, confidence intervals, and significance for the *WWOX*/*HIF1A* ratio, gene expressions, age, and stage; Supplementary Figure S5: Multivariate Cox Regression analysis of prognostic factors in hepatocellular carcinoma. Forest plot visualizing hazard ratios, confidence intervals, and significance for the *WWOX*/*HIF1A* ratio, gene expressions, age, and stage; Supplementary Figure S6: Multivariate Cox Regression analysis of prognostic factors in glioblastoma. Forest plot visualizing hazard ratios, confidence intervals, and significance for the *WWOX*/*HIF1A* ratio, gene expressions, age, and stage; Supplementary Figure S7: Multivariate Cox Regression analysis of prognostic factors in low-grade glioma. Forest plot visualizing hazard ratios, confidence intervals, and significance for the *WWOX*/*HIF1A* ratio, gene expressions, age, and stage; Supplementary File S1: Complete list of genes with corresponding SR plot output data for each cancer type, covering both good and poor prognoses.

## Abbreviations

The following abbreviations are used in this manuscript:

GBM	Glioblastoma multiforme
LGG	Low-Grade Glioma
HCC	Hepatocellular Carcinoma
ECM	Extracellular matrix
VHL	Von Hippel–Lindau
OXPHOS	Oxidative Phosphorylation
TME	Tumour Microenvironment
NK	Natural Killer
TNBC	Triple Negative Breast Cancer
NAFLD	Non-alcoholic Fatty Liver Disease
TCGA	The Cancer Genome Atlas
GDC	Genomic Data Commons
FDR	False Discovery Rate
EMT	Epithelial–Mesenchymal Transition
KEGG	Kyoto Encyclopedia of Genes and Genomes
TCA	Tricarboxylic Acid Cycle
ROS	Reactive Oxygen Species
