# Supplementary material for: Prognostic Significance of WWOX/HIF1A Ratio in Cancer Subtypes: Insights into Metabolism, ECM, and EMT
Source: Biology (Basel). 2025 Sep 1;14(9):1151. doi: 10.3390/biology14091151 (PMC12467568; doi:10.3390/biology14091151)
Supplement: Supplementary file 1 [file biology-14-01151-s001.zip › biology-3780101_Supplementary tables and figures.pdf]

Supplementary Table S1. TCGA patient cohort clinical information.

**Hepatocellular carcinoma**		
**parameter**	**Below WWOX/HIF1A ratio (120 patients)**	**Above WWOX/HIF1A ratio (251 patients)**
**Median age**	58	63
**race**	White :60<br>Asian:55<br>Black or African American:3<br>NA:2	White :124<br>Asian:103<br>Black or African American:14<br>NA:10
**DFS**	With tumour:49<br>Without tumour: 64<br>NA: 7	With tumour:62<br>Without tumour: 170<br>NA: 19
**Histological type**	hepatocellular carcinoma:116<br>hepatocholangiocarcinoma (mixed):4	hepatocellular carcinoma 245<br>hepatocholangiocarcinoma (mixed) 3<br>Na:3
**Gender**	Females:46<br>Males:74	Females:75<br>Males:176

**GBM**		
**parameter**	**Below WWOX/HIF1A ratio(63 patients)**	**Above WWOX/HIF1A ratio(96 patients)**
**Median age**	60	62

**race**	White :57<br>Asian:3<br>Black or African American: 3<br>Na:-	White :85<br>Asian:2<br>Black or African American:8<br>Na:1
**DFS**	With tumour:53<br>Without tumour: 4<br>NA: 6	With tumour:9<br>Without tumour: 77<br>NA: 10
**Histological type**	Untreated primary de novo gbm:62<br>Glioblastoma multiform:1	Untreated primary de novo gbm:95<br>treated primary gbm:1
**Gender**	Females:20<br>Males:43	Females:35<br>Males:61

**LGG**		
**parameter**	**Below WWOX/HIF1A ratio(40 patients)**	**Above WWOX/HIF1A ratio (475 patients)**
**Median age**	41.5	41
**race**	White :38<br>Asian:1<br>Black or African American:-<br>Na:1	White :437<br>Asian:7<br>Black or African American:21<br>Na:10
**DFS**	With tumour:30<br>Without tumour: 6<br>NA: 4	With tumour:245<br>Without tumour: 175<br>NA: 55
**Histological type**	Astrocytoma 24, oligoastrocytoma 5, oligodendroglioma 11	Astrocytoma 170, oligodendroglioma 180, oligoastrocytoma 125

**Gender**	Females:19<br>Males:21	Females:211<br>Males:264

BASAL			
PARAMETER		Below WWOX/HIF1A ratio	Above WWOX/HIF1A ratio
MEDIAN AGE		55	53
RACE	WHITE	38	35
	BLACK OR AFRICAN AMERICAN	10	2
	AMERICAN INDIAN OR ALASKA NATIVE	0	0
	NOT AVAILABLE	5	2
	ASIAN	5	0
DISEASE FREE STATUS	WITH TUMOUR	8	0
	TUMOUR FREE	42	34
	NA	8	5
HISTOLOGICAL TYPE	Breast Invasive Carcinoma	0	1
	Infiltrating Ductal Carcinoma	54	35
	Infiltrating Lobular Carcinoma	0	1

	Medullary Carcinoma	1	0
	Mixed Histology (NOS)	0	0
	Mucinous Carcinoma	0	0
	Other	3	2
GENDER	FEMALE	58	39
	MALE	0	0
MENOPAUSE STATUS	Pre (<6 months since LMP AND no prior bilateral ovariectomy AND not on estrogen replacement)	13	12
	Peri (6-12 months since last menstrual period)	2	1
	Post (prior bilateral ovariectomy OR >12 mo since LMP with no prior hysterectomy)	34	18
	Indeterminate (neither Pre or Postmenopausal)	5	6
	NOT AVAILABLE	4	2
ESTROGEN RECEPTOR STATUS	POSITIVE	6	8
	NEGATIVE	51	30
	Performed but Not Available or Not Available	1	1
	INDETERMINATE	0	0

PROGESTERON RECEPTOR STATUS	POSITIVE	5	4
	NEGATIVE	51	34
	Performed but Not Available or Not Available	1	1
	INDETERMINATE	1	0

HER2			
**PARAMETER**		Below WWOX/HIF1A ratio	Above WWOX/HIF1A ratio
**MEDIAN AGE**		56	55
**RACE**	WHITE	8	19
	BLACK OR AFRICAN AMERICAN	0	4
	AMERICAN INDIAN OR ALASKA NATIVE	0	1
	NOT AVAILABLE	0	15
	ASIAN	3	8
**DISEASE FREE STATUS**	WITH TUMOUR	35	11
	TUMOUR FREE	8	0
	NA	4	0

**HISTOLOGICAL TYPE**	Breast Invasive Carcinoma	1	1
	Infiltrating Ductal Carcinoma	43	10
	Infiltrating Lobular Carcinoma	3	
	Medullary Carcinoma	0	0
	Mixed Histology (NOS)	0	0
	Mucinous Carcinoma	0	0
	Other	0	0
**GENDER**	FEMALE	47	11
	MALE	0	0
**MENOPAUSE STATUS**	Pre (<6 months since LMP AND no prior bilateral ovariectomy AND not on estrogen replacement)	11	5
	Peri (6-12 months since last menstrual period)	2	0
	Post (prior bilateral ovariectomy OR >12 mo since LMP with no prior hysterectomy)	27	6
	Indeterminate (neither Pre or Postmenopausal)	4	0
	NOT AVAILABLE	3	0

**ESTROGEN RECEPTOR STATUS**	POSITIVE	27	4
	NEGATIVE	18	7
	Performed but Not Available or Not Available	0	0
	INDETERMINATE	2	0
**PROGESTERON RECEPTOR STATUS**	POSITIVE	17	4
	NEGATIVE	30	7
	Performed but Not Available or Not Available	0	0
	INDETERMINATE	0	0

Luminal A			
**PARAMETER**		**Below WWOX/HIF1A ratio**	**Above WWOX/HIF1A ratio**
**MEDIAN AGE**		57	61
**RACE**	WHITE	104	72
	BLACK OR AFRICAN AMERICAN	9	4
	AMERICAN INDIAN OR ALASKA NATIVE	0	0
	NOT AVAILABLE	18	12
	ASIAN	3	6

**DISEASE FREE STATUS**	WITH TUMOUR	8	7
	TUMOUR FREE	114	79
	NA	12	8
**HISTOLOGICAL TYPE**	Breast Invasive Carcinoma	1	0
	Infiltrating Ductal Carcinoma	108	71
	Infiltrating Lobular Carcinoma	16	14
	Medullary Carcinoma	0	0
	Mixed Histology (NOS)	6	3
	Mucinous Carcinoma	1	0
	Other	2	6
**GENDER**	FEMALE	134	94
	MALE	0	0
**MENOPAUSE STATUS**	Pre (<6 months since LMP AND no prior bilateral ovariectomy AND not on estrogen replacement)	37	12
	Peri (6-12 months since last menstrual period)	5	4
	Post (prior bilateral ovariectomy OR >12 mo since LMP with no prior hysterectomy)	82	64

	Indeterminate (neither Pre or Postmenopausal)	4	11
	NOT AVAILABLE	6	3
**ESTROGEN RECEPTOR STATUS**	POSITIVE	132	86
	NEGATIVE	1	6
	Performed but Not Available or Not Available	1	2
	INDETERMINATE	0	0
**PROGESTERON RECEPTOR STATUS**	POSITIVE	126	78
	NEGATIVE	7	12
	Performed but Not Available or Not Available	1	2
	INDETERMINATE	0	2

luminal B			
**PARAMETER**		**Below WWOX/HIF1A ratio**	**Above WWOX/HIF1A ratio**
**MEDIAN AGE**		57	61
**RACE**	WHITE	25	45
	BLACK OR AFRICAN AMERICAN	4	6
	AMERICAN INDIAN OR ALASKA NATIVE	0	0

	NOT AVAILABLE	11	22
	ASIAN	3	6
**DISEASE FREE STATUS**	WITH TUMOUR	5	10
	TUMOUR FREE	34	57
	NA	4	10
**HISTOLOGICAL TYPE**	Breast Invasive Carcinoma	1	1
	Infiltrating Ductal Carcinoma	36	68
	Infiltrating Lobular Carcinoma	4	4
	Medullary Carcinoma	0	0
	Mixed Histology (NOS)	1	1
	Mucinous Carcinoma	0	1
	Other	1	4
**GENDER**	FEMALE	43	79
	MALE	0	0
**MENOPAUSE STATUS**	Pre (<6 months since LMP AND no prior bilateral ovariectomy AND not on estrogen replacement)	11	13
	Peri (6-12 months since last menstrual period)	1	2

	Post (prior bilateral ovariectomy OR >12 mo since LMP with no prior hysterectomy)	31	57
	Indeterminate (neither Pre or Postmenopausal)	0	3
	NOT AVAILABLE	0	4
**ESTROGEN RECEPTOR STATUS**	POSITIVE	42	79
	NEGATIVE	1	0
	Performed but Not Available or Not Available	0	0
	INDETERMINATE	0	0
**PROGESTERON RECEPTOR STATUS**	POSITIVE	31	63
	NEGATIVE	12	16
	Performed but Not Available or Not Available	0	0
	INDETERMINATE	0	0

Supplementary table S2. Genes involved in major cancer-related pathways and their expression in Basal breast cancer subtype in relation to prognostic WWOX/HIF1A ratio.

**ECM Genes**	**Ratio good prognosis**	**Ratio bad prognosis**	**Log2fc**
CCR7	68.37	119.13	-0.80
HIF1A	2277.80	3763.00	-0.72
PLEKHA2	602.91	931.90	-0.63
KDR	514.26	766.50	-0.58
CCL25	1.44	0.93	0.63
TGFB2	387.35	216.48	0.84
WWOX	369.64	163.81	1.17
RATIO WWOX/HIF1A	0.17	0.05	1.86

**Mesenchymal Genes**	**Ratio good prognosis**	**Ratio bad prognosis**	**Log2fc**
FOXC2	11.16	22.66	-1.02
DDR2	135.52	263.50	-0.96
CDH11	787.47	1330.29	-0.76
FN1	48561.63	70172.95	-0.53
RATIO WWOX/HIF1A	0.17	0.05	1.86

**Epithelial Genes**	**Ratio good prognosis**	**Ratio bad prognosis**	**Log2fc**
MUC1	1373.11	3196.00	-1.22
LAMA3	203.45	412.65	-1.02
LAMA1	55.07	91.98	-0.74
LAMA2	127.89	210.58	-0.72
RATIO WWOX/HIF1A	0.17	0.05	1.86

**Metabolism Genes**	**Ratio good prognosis**	**Ratio bad prognosis**	**Log2fc**
PIK3CG	41.52	112.22	-1.43
PRKCB	100.57	192.55	-0.94
LDHAL6A	0.22	0.39	-0.81
ADH1A	0.29	0.50	-0.78
ADH1B	23.23	38.51	-0.73
HIF1A	2277.80	3763.00	-0.72
ADH1C	0.61	0.91	-0.58
KDR	514.26	766.50	-0.58
SLC2A1	1522.16	2266.60	-0.57
LDHC	9.78	14.49	-0.57
GAPDHS	1.52	2.16	-0.51
ALDH3B2	560.96	388.92	0.53
ENO2	481.45	330.30	0.54
ALDOB	1.93	1.31	0.56
GCK	1.96	1.22	0.69
WWOX	369.64	163.81	1.17
RATIO *WWOX/HIF1A*	0.17	0.05	1.87
OGDHL	16.42	3.31	2.31

Supplementary table S3. Genes involved in major cancer-related pathways and their expression in HER2 breast cancer subtype in relation to prognostic WWOX/HIF1A ratio.

ECM Genes	Ratio good prognosis	Ratio bad prognosis	Log2fc
TGFB2	199.15	327.72	-0.72
PIK3R1	4058.87	2194.43	0.89
WWOX	555.13	199.14	1.48
RATIO WWOX/HIF1A	0.19	0.05	1.80

Epithelial Genes	Ratio good prognosis	Ratio bad prognosis	Log2fc
LAMA1	39.44	77.65	-0.98
KRT5	324.41	621.41	-0.94
LAMA4	1571.75	2402.64	-0.61
KRT8	20971.55	29691.15	-0.50
LAMA3	254.84	177.61	0.52
RATIO WWOX/HIF1A	0.19	0.05	1.80

Mesenchymal Genes	Ratio good prognosis	Ratio bad prognosis	Log2fc
DDR2	86.43	226.88	-1.39

MMP9	594.67	1279.36	-1.11
MMP3	124.52	261.52	-1.07
FN1	91950.89	162096.94	-0.82
CDH11	1829.75	3048.04	-0.74
SNAI1	87.51	126.03	-0.53
ITGB6	2623.9	1658.09	0.66
GSC	29.66	18.02	0.72
FOXC2	9.61	5.54	0.79
RATIO WWOX/HIF1A	0.19	0.05	1.8

Metabolism Genes	Ratio good prognosis	Ratio bad prognosis	log2fc
OGDHL	0.99	3.17	-1.68
PIK3CG	34.19	105.25	-1.62
ALDOC	105.33	242.79	-1.20
GAPDHS	0.40	0.91	-1.18
VEGFA	1217.25	2504.42	-1.04
LDHAL6B	0.54	1.10	-1.02
PFKFB2	358.06	677.54	-0.92
GCK	0.92	1.68	-0.86
HK3	50.27	87.89	-0.81
SLC2A1	1292.59	2140.97	-0.73
ADH1B	33.34	52.77	-0.66
ACLY	3510.88	5514.76	-0.65
PRKCA	78.68	122.46	-0.64
PRKCB	92.38	142.64	-0.63
PCK1	2.32	3.48	-0.59
LDHB	1163.92	1730.19	-0.57
PTK2	2102.93	2993.05	-0.51
LDHAL6A	0.37	0.52	-0.51
PC	774.75	501.17	0.63
ALDH3A1	1.78	1.14	0.64
PCK2	2007.03	1150.76	0.80
ENO3	40.39	23.03	0.81
HRAS	865.92	486.44	0.83
PIK3R1	4058.87	2194.43	0.89

PFKP	2434.84	1314.90	0.89
ADH6	0.47	0.23	1.05
ALDH3B2	5756.22	2570.61	1.16
WWOX	555.13	199.14	1.48
RATIO WWOX/HIF1A	0.19	0.05	1.80
ADH1C	36.05	6.84	2.40
LDHC	32.96	3.83	3.10

Supplementary tables S4. Genes involved in major cancer-related pathways and their expression in luminal A breast cancer subtype in relation to prognostic WWOX/HIF1A ratio.

**Epithelial Genes**	**Ratio good prognosis**	**Ratio bad prognosis**	**Log2fc**
RATIO *WWOX/HIF1A*	0.074	0.31	-2.04

**Warburg Genes**	**Ratio good prognosis**	**Ratio bad prognosis**	**Log2fc**
RATIO *WWOX/HIF1A*	0.07	0.31	-2.04
WWOX	167.20	422.78	-1.34
LDHC	2.48	5.24	-1.08
ALDH3A1	1.93	3.01	-0.64
G6PC2	0.27	0.41	-0.62
HK3	29.74	20.40	0.54
LDHAL6A	0.80	0.55	0.55
ADH6	0.51	0.32	0.65
PRKCB	116.22	65.73	0.82
HIF1A	2369.90	1115.35	1.09
ADH1C	15.72	6.77	1.22

**ECM Genes**	**Ratio good prognosis**	**Ratio bad prognosis**	**Log2fc**
RATIO *WWOX/HIF1A*	0.07	0.31	-2.04
WWOX	167.20	422.78	-1.34
CCR7	75.46	41.11	0.88
HIF1A	2369.90	1115.35	1.09

Mesenchymal Genes	Ratio good prognosis	Ratio bad prognosis	log2fc
CDH2	87.15	58.55	0.57
DDR2	256.41	165.86	0.63

Supplementary tables S5. Genes involved in major cancer-related pathways and their expression in luminal B breast cancer subtype in relation to prognostic WWOX/HIF1A ratio.

Epithelial Genes	Ratio good prognosis	Ratio bad prognosis	Log2fc
RATIO *WWOX/HIF1A*	0.04	0.24	-2.53
LAMA2	425.70	279.72	0.61
KRT5	228.42	80.50	1.50

Mesenchymal Genes	Ratio good prognosis	Ratio bad prognosis	Log2fc
CDH2	76.12	53.58	0.51

ECM Genes	Ratio good prognosis	Ratio bad prognosis	log2fc
RATIO *WWOX/HIF1A*	0.04	0.24	-2.53
WWOX	120.81	295.82	-1.29
PIK3R1	1506.84	2136.71	-0.50
TGFB2	360.08	235.65	0.61
HIF1A	3015.56	1355.62	1.15

Metabolism Genes	Ratio good prognosis	Ratio bad prognosis	Log2fc
RATIO *WWOX/HIF1A*	0.04	0.24	-2.53
WWOX	120.81	295.82	-1.29
PFKFB1	6.76	11.35	-0.75
ALDH3A1	1.17	1.91	-0.70
PGAM2	16.73	26.66	-0.67
SLC2A1	1385.29	2096.46	-0.60
ENO2	865.57	1280.36	-0.56
FBP2	0.26	0.38	-0.56
PIK3R1	1506.84	2136.71	-0.50
LDHB	1874.66	1275.70	0.56
LDHAL6B	0.86	0.58	0.56
ALDH3B2	2524.61	1458.44	0.79
PCK1	7.69	3.61	1.09
ADH1B	149.43	69.86	1.10
HIF1A	3015.56	1355.62	1.15
ADH1A	1.08	0.46	1.22
ADH1C	4.41	1.79	1.30
OGDHL	2.69	0.93	1.53
LDHC	3.56	0.73	2.28

Supplementary tables S6. Genes involved in major cancer-related pathways and their expression in hepatocellular carcinoma subtype in relation to prognostic WWOX/HIF1A ratio.

**ECM Genes**	**Ratio good prognosis**	**Ratio bad prognosis**	**Log2fc**
HIF1A	1053.68	2530.65	-1.26
TGFB2	63.91	138.54	-1.12
SRC	542.97	977.04	-0.85
CSF1	272.34	484.31	-0.83
TGFB1	532.99	924.02	-0.79
PLEKHA2	243.96	394.83	-0.69
CCR7	18.59	26.23	-0.5
SMAD3	1066.13	1493.57	-0.49
SLK	676.14	909.88	-0.43
ABL1	979.21	1316.61	-0.43
VEGFC	91.95	120.48	-0.39
RAC1	4610.94	5167.48	-0.16
SDC4	9218.47	10261.73	-0.15
DAG1	3842.78	4182.01	-0.12
HSPA8	19598.69	21090.45	-0.11
KDR	626.06	654.17	-0.06
PPM1F	801.72	779.44	0.04
PIK3R1	2136.69	1987.87	0.1
PTK2B	401.72	370.51	0.12

WWOX	298.19	181.23	0.72
CCL25	19.42	9.2	1.08
RATIO WWOX/HIF1A	0.27	0.08	1.81

**Epithelial Genes**	**Ratio good prognosis**	**Ratio bad prognosis**	**Log2fc**
KRT19	15.90	136.06	-3.10
LAMA1	1.95	6.44	-1.72
HIF1A	1053.68	2530.65	-1.26
LAMA2	78.51	187.13	-1.25
MUC1	19.10	41.68	-1.13
COL4A1	3858.30	7496.73	-0.96
LAMA5	1080.79	1903.72	-0.82
LAMA4	493.70	808.02	-0.71
OCLN	508.41	779.84	-0.62
KRT5	1.06	1.60	-0.59
NID1	3358.34	4775.35	-0.51
DSP	5456.01	6653.56	-0.29
CDH1	4104.75	4695.98	-0.19
KRT8	18188.17	18861.04	-0.05
KRT18	16193.34	15658.33	0.05
LAMA3	316.96	251.83	0.33
WWOX	298.19	181.23	0.72
RATIO WWOX/HIF1A	0.27	0.08	1.81

**Mesenchymal Genes**	**Ratio good prognosis**	**Ratio bad prognosis**	**log2fc**
ITGB6	2.34	10.94	-2.22
CDH11	52.32	172.49	-1.72
FOXC2	2	5.41	-1.44
MMP9	129.65	342.19	-1.4
DDR2	12.78	33.7	-1.4
MMP2	350.64	913.8	-1.38
HIF1A	1053.68	2530.65	-1.26

TWIST1	3.09	6.86	-1.15
ZEB2	169.35	304.92	-0.85
SERPINH1	1929.86	3402.83	-0.82
SNAI1	44.36	77.61	-0.81
GSC	0.41	0.68	-0.74
ITGA5	2592.76	4164.03	-0.68
VIM	5529.89	8581.58	-0.63
LEF1	82.43	123.23	-0.58
TCF4	331.58	494.34	-0.58
SDC1	23430.02	15809.97	0.57
WWOX	298.19	181.23	0.72
RATIO WWOX/HIF1A	0.27	0.08	1.81

Metabolism Genes	Ratio good prognosis	Ratio bad prognosis	Log2fc
PFKFB3	305.10	919.60	-1.59
HK2	54.05	149.58	-1.47
HIF1A	1053.68	2524.91	-1.26
ALDH1A3	34.19	75.74	-1.15
PKM2	1953.39	4125.78	-1.08
ALDH3B2	0.57	1.20	-1.08
ENO2	43.67	87.32	-1.00
LDHAL6B	0.57	1.11	-0.96
PIK3CG	14.22	26.73	-0.91
PFKP	134.48	244.66	-0.86
ACSS1	280.58	470.77	-0.75
ALDH3B1	230.63	384.12	-0.74
PGM2	349.41	546.34	-0.64
PRKCB	43.76	68.28	-0.64
HK1	322.05	489.63	-0.60
PFKFB2	239.68	345.65	-0.53
SUCLG2	3229.32	2256.75	0.52
FH	6826.24	4571.86	0.58
AKR1A1	5769.62	3848.20	0.58
PGM1	4957.34	3276.72	0.60
ALDH3A2	8451.58	5536.30	0.61

PKLR	4631.00	2957.73	0.65
ACSS2	2575.23	1623.14	0.67
SDHB	3398.23	2114.04	0.68
WWOX	298.19	183.52	0.70
FBP1	5254.04	3192.45	0.72
ADH7	1.07	0.65	0.72
GCK	4.30	2.55	0.76
ALDH7A1	4671.85	2729.88	0.78
PC	6478.37	3770.23	0.78
ADH6	5295.28	2804.43	0.92
ADH1A	13316.06	6987.90	0.93
ALDOB	84980.60	41020.01	1.05
ALDH2	26134.36	12528.55	1.06
G6PC	10816.42	4932.58	1.13
PCK2	12111.94	5285.82	1.20
ALDH3A1	124.20	53.97	1.20
LDHC	4.79	1.84	1.38
OGDHL	2471.64	895.94	1.46
ADH1B	43018.29	14627.70	1.56
RATIO *WWOX/HIF1*	0.27	0.08	1.80
PFKFB1	372.68	100.60	1.89
PCK1	15115.44	3848.89	1.97
ADH1C	10783.13	2507.31	2.10
ADH4	13340.74	1722.55	2.95

Supplementary table S7. Genes involved in major cancer-related pathways and their expression in glioblastoma in relation to prognostic WWOX/HIF1A ratio.

**Epithelial Genes**	**Ratio good prognosis**	**Ratio bad prognosis**	**Log2fc**
HIF1A	4312.00	7472.91	-0.79
KRT8	4.68	7.66	-0.71
LAMA2	584.44	942.35	-0.69
LAMA4	1314.92	2085.21	-0.67
COL4A1	6662.68	9704.88	-0.54
WWOX	411.84	260.44	0.66
KRT5	4.94	2.59	0.93
RATIO WWOX/HIF1A	0.09	0.04	1.22
CDH1	96.77	32.75	1.56

**ECM Genes**	**Ratio good prognosis**	**Ratio bad prognosis**	**Log2fc**
HIF1A	4311.99	7472.91	-0.79
CSF1	923.99	1429.35	-0.63
SDC4	1390.64	2022.07	-0.54
WWOX	411.84	260.44	0.66

RATIO WWOX/HIF1A	0.0887	0.038	1.22

**Mesenchymal genes**	**Ratio good prognosis**	**Ratio bad prognosis**	**Log2fc**
CDH11	1436.06	2041.07	-0.51
SDC1	257.20	375.67	-0.55
MMP3	1.33	2.02	-0.60
DDR2	152.79	232.55	-0.61
ITGA5	1261.96	1930.33	-0.61
HIF1A	4312.00	7472.91	-0.79
SNAI1	38.66	71.79	-0.89
RATIO WWOX/HIF1A	0.09	0.04	1.22
WWOX	411.84	260.44	0.66

**Metabolism Genes**	**Ratio good prognosis**	**Ratio bad prognosis**	**Log2fc**
PIK3CG	63.07	128.67	-1.03
HK3	88.43	167.85	-0.92
LDHAL6B	0.54	1.00	-0.90
ALDH1A3	27.81	51.40	-0.89
HIF1A	4312.00	7472.91	-0.79
SHC1	1509.72	2327.29	-0.62
FLT4	215.97	306.03	-0.50
ACSS1	1006.42	711.27	0.50
PFKFB2	934.99	628.78	0.57
OGDHL	105.99	70.79	0.58
GCK	105.07	70.01	0.59
PGAM2	401.29	267.14	0.59
WWOX	411.84	260.44	0.66
LDHAL6A	0.91	0.55	0.72
ALDH3A1	20.14	11.40	0.82
PKLR	1.09	0.60	0.87
ALDOC	7341.96	3958.58	0.89
ADH1B	2.03	1.00	1.03
RATIO WWOX/HIF1A	0.09	0.04	1.22

Supplementary tables S8. Genes involved in major cancer-related pathways and their expression in low grade glioma in relation to prognostic WWOX/HIF1A ratio.

**ECM Genes**	**Ratio good prognosis**	**Ratio bad prognosis**	**Log2fc**
TGFB2	339.14	916.77	-1.43
HIF1A	4238.50	8142.79	-0.94
KDR	361.09	680.04	-0.91
CCR7	3.26	5.85	-0.85
WWOX	614.83	348.85	0.82
RATIO WWOX/HIF1A	0.15	0.05	1.62

**Epithelial Genes**	**Ratio good prognosis**	**Ratio bad prognosis**	**log2fc**
RATIO WWOX/HIF1A	0.15	0.05	-4.38
KRT5	5.58	2.05	1.03
KRT19	6.44	3.17	1.67
KRT8	2.31	5.63	2.49
KRT18	6.60	17.47	4.13
LAMA3	46.45	38.70	5.27

OCLN	48.05	48.19	5.59
CDH1	71.33	53.08	5.73
DSP	99.47	147.63	7.21
MUC1	96.19	148.95	7.22
LAMA1	199.66	234.78	7.88
WWOX	614.83	348.85	8.45
LAMA2	249.19	616.85	9.27
LAMA4	652.17	1344.54	10.39
LAMA5	1157.68	1807.10	10.82
NID1	851.93	1888.74	10.88
TJP1	4166.04	4101.11	12.00
COL4A1	544.43	5261.51	12.36
HIF1A	4238.50	8142.79	12.99

**Mesenchymal Genes**	**Ratio good prognosis**	**Ratio bad prognosis**	**Log2fc**
MMP9	4.33	43.85	-3.34
S100A4	35.56	160.56	-2.18
SDC1	55.82	230.90	-2.05
SERPINH1	464.24	1601.25	-1.79
VIM	11809.28	38170.72	-1.69
ITGA5	307.00	909.25	-1.57
GSC	15.09	43.62	-1.53
FN1	4827.46	13898.03	-1.53
TWIST1	17.11	40.66	-1.25
SNAI2	32.89	71.17	-1.11
CDH11	993.46	2055.20	-1.05
HIF1A	4238.50	8142.79	-0.94
DDR2	147.20	279.78	-0.93
SNAI1	12.69	24.12	-0.93
LEF1	202.55	360.69	-0.83
ACTA2	456.03	748.01	-0.71
FOXC2	3.90	6.18	-0.66
CDH2	2484.81	3675.16	-0.56
WWOX	614.83	348.85	0.82

RATIO WWOX/HIF1A	0.15	0.05	1.62

Metabolism Genes	Ratio good prognosis	Ratio bad prognosis	Log2fc
HK3	14.81	46.24	-1.64
VEGFA	605.45	1479.62	-1.29
FLT4	139.53	304.28	-1.12
HIF1A	4238.50	8142.79	-0.94
ALDH1A3	6.88	13.13	-0.93
KDR	361.09	680.04	-0.91
SHC1	723.18	1350.36	-0.90
PIK3CG	32.68	59.67	-0.87
PGM2	342.00	574.72	-0.75
ALDH3B2	0.40	0.66	-0.72
LDHA	2437.81	3680.64	-0.59
HK2	596.01	877.06	-0.56
NOS3	111.01	158.20	-0.51
LDHB	17394.87	11772.42	0.56
ENO2	5532.85	3626.75	0.61
ACO2	6191.51	4043.79	0.61
PFKFB2	1226.29	788.02	0.64
PRKCB	1028.68	597.23	0.78
ACSS1	1630.67	934.39	0.80
WWOX	614.83	348.85	0.82
PCK1	2.33	1.28	0.87
PC	2172.96	1115.81	0.96
ALDH2	7904.40	3430.45	1.20
G6PC	0.95	0.40	1.23
ADH1B	12.25	4.97	1.30
PKLR	1.44	0.58	1.32
OGDHL	211.78	78.40	1.43
RATIO WWOX/HIF1A	0.15	0.05	1.62
G6PC2	3.74	0.82	2.20
ALDOC	23338.43	5012.09	2.22

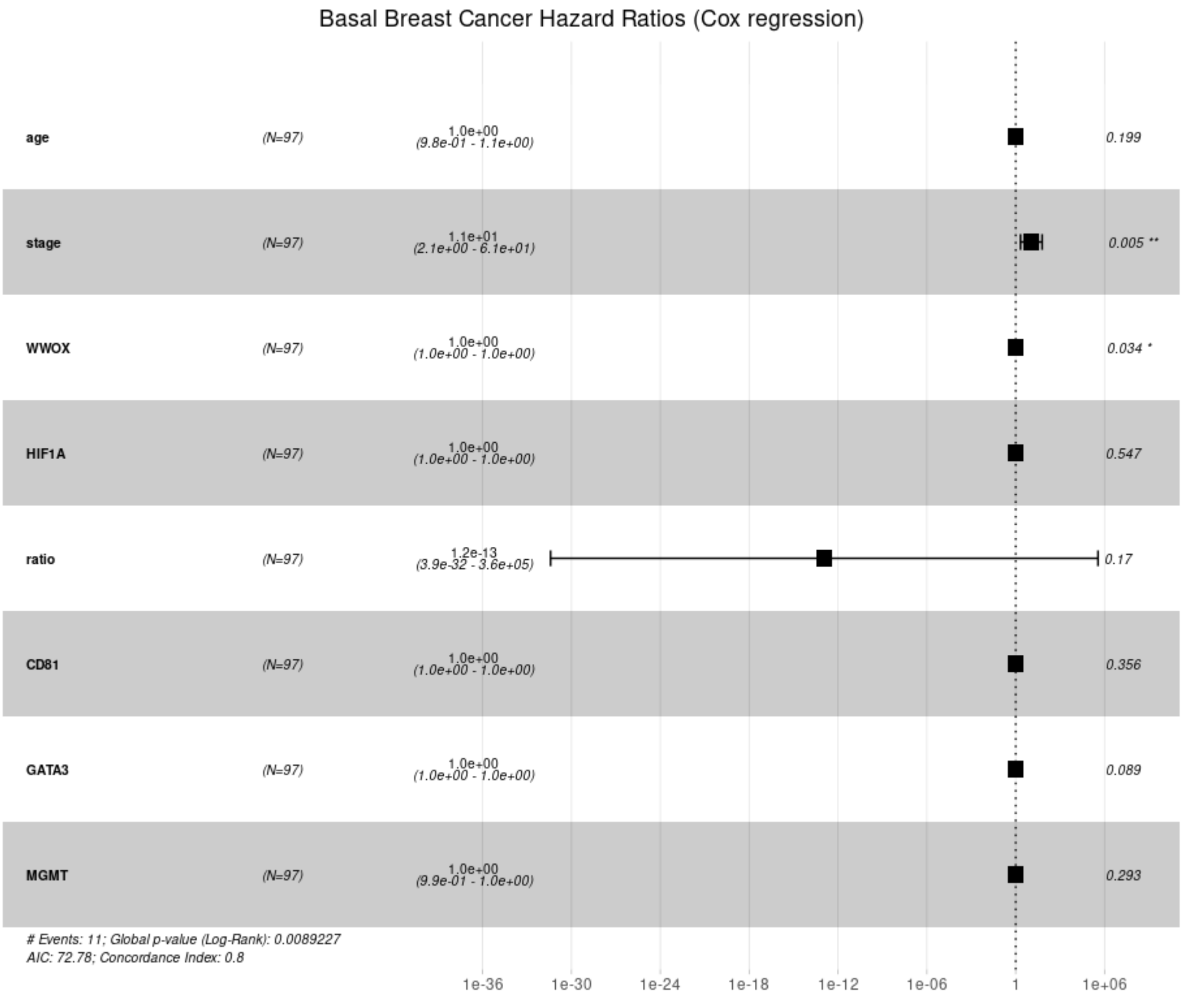

Supplementary Figure S1. Multivariate Cox Regression analysis of prognostic factors in basal breast cancer. Forest plot visualizing hazard ratios, confidence intervals, and significance for the WWOX/HIF1A ratio, gene expressions, age, and stage.

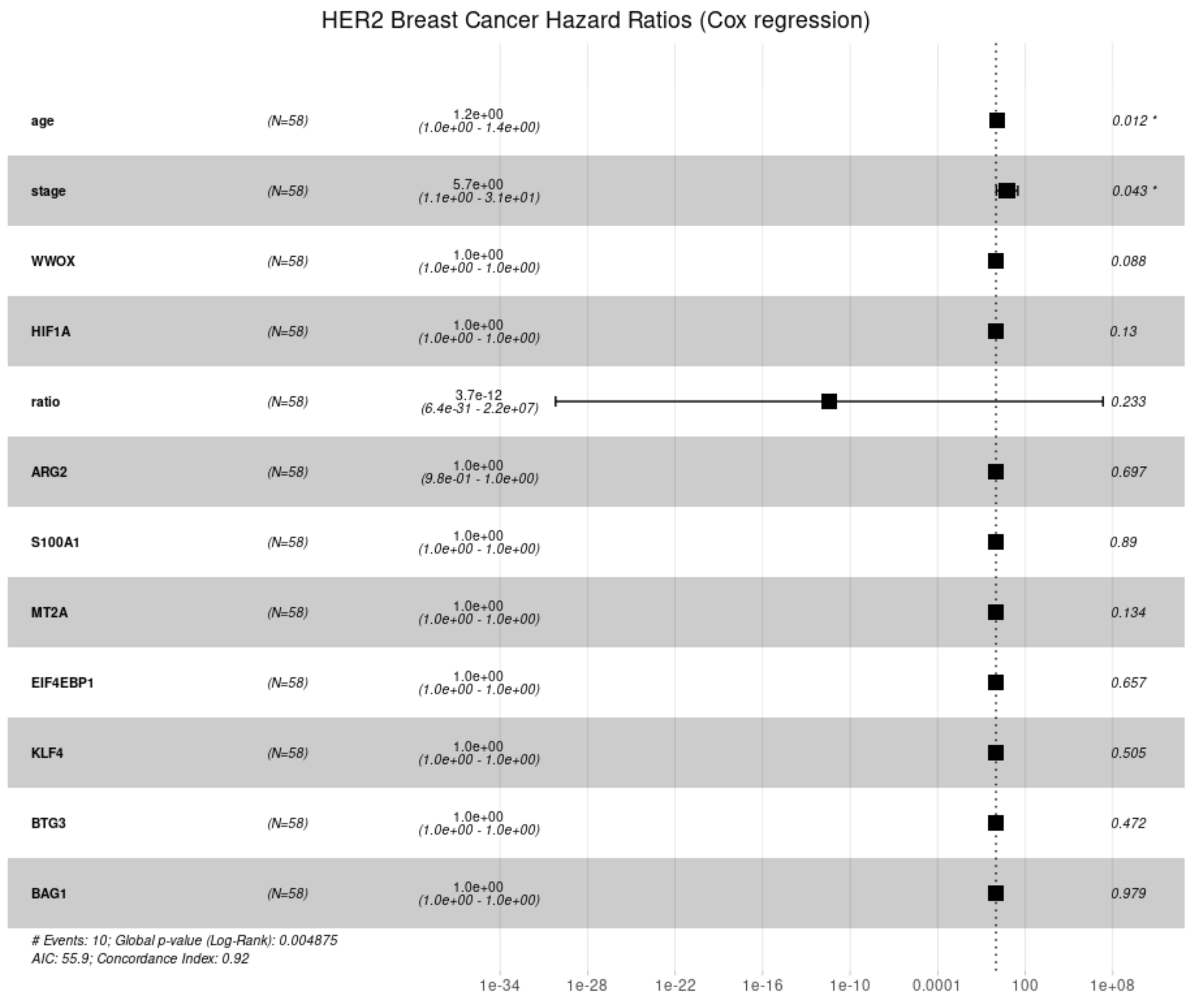

Supplementary Figure S2. Multivariate Cox Regression analysis of prognostic factors in Her2 breast cancer. Forest plot visualizing hazard ratios, confidence intervals, and significance for the WWOX/HIF1A ratio, gene expressions, age, and stage.

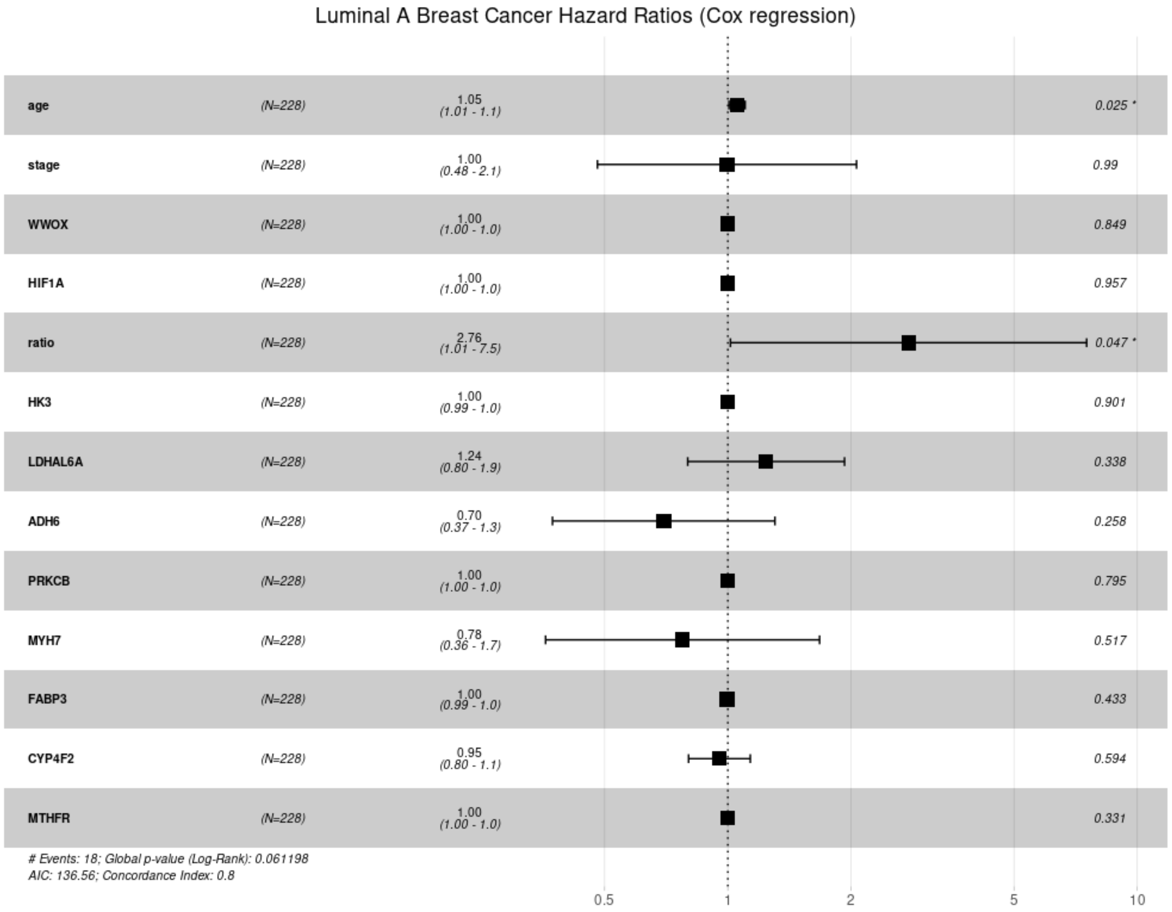

Supplementary Figure S3. Multivariate Cox Regression analysis of prognostic factors in Luminal A breast cancer subtype. Forest plot visualizing hazard ratios, confidence intervals, and significance for the WWOX/HIF1A ratio, gene expressions, age, and stage.

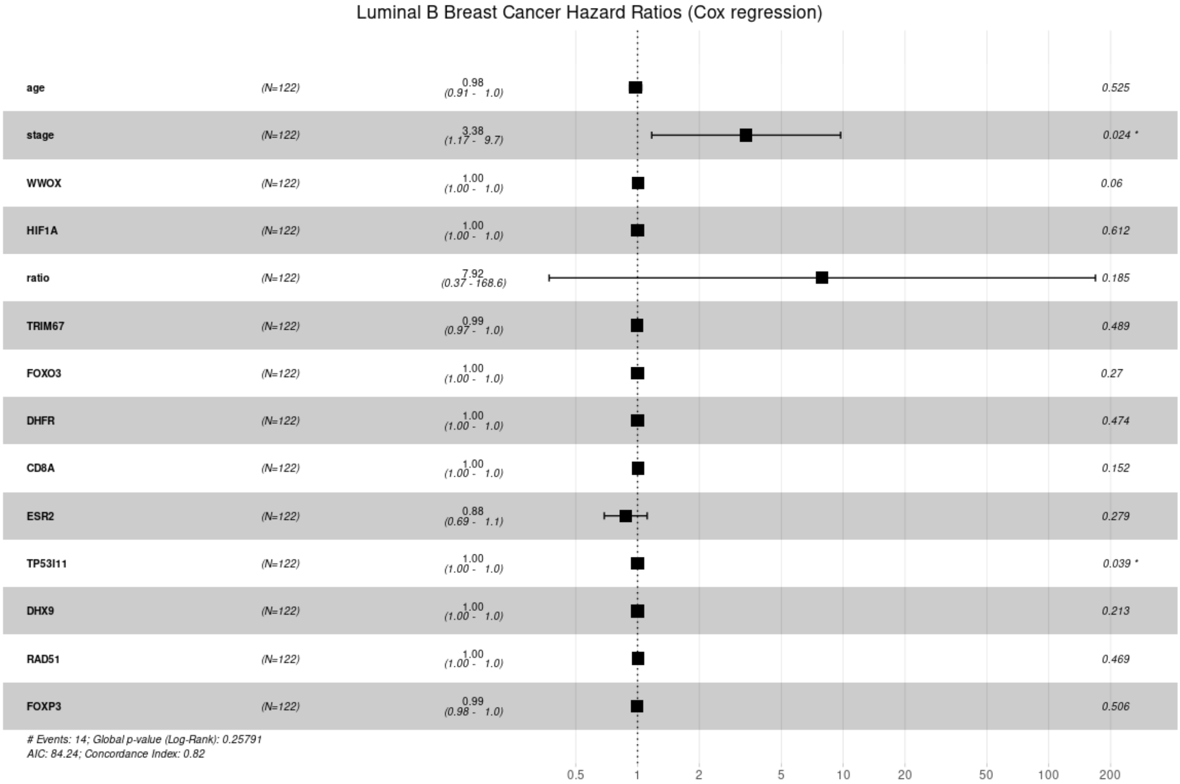

Supplementary Figure S4. Multivariate Cox Regression analysis of prognostic factors in Luminal B breast cancer subtype. Forest plot visualizing hazard ratios, confidence intervals, and significance for the WWOX/HIF1A ratio, gene expressions, age, and stage.

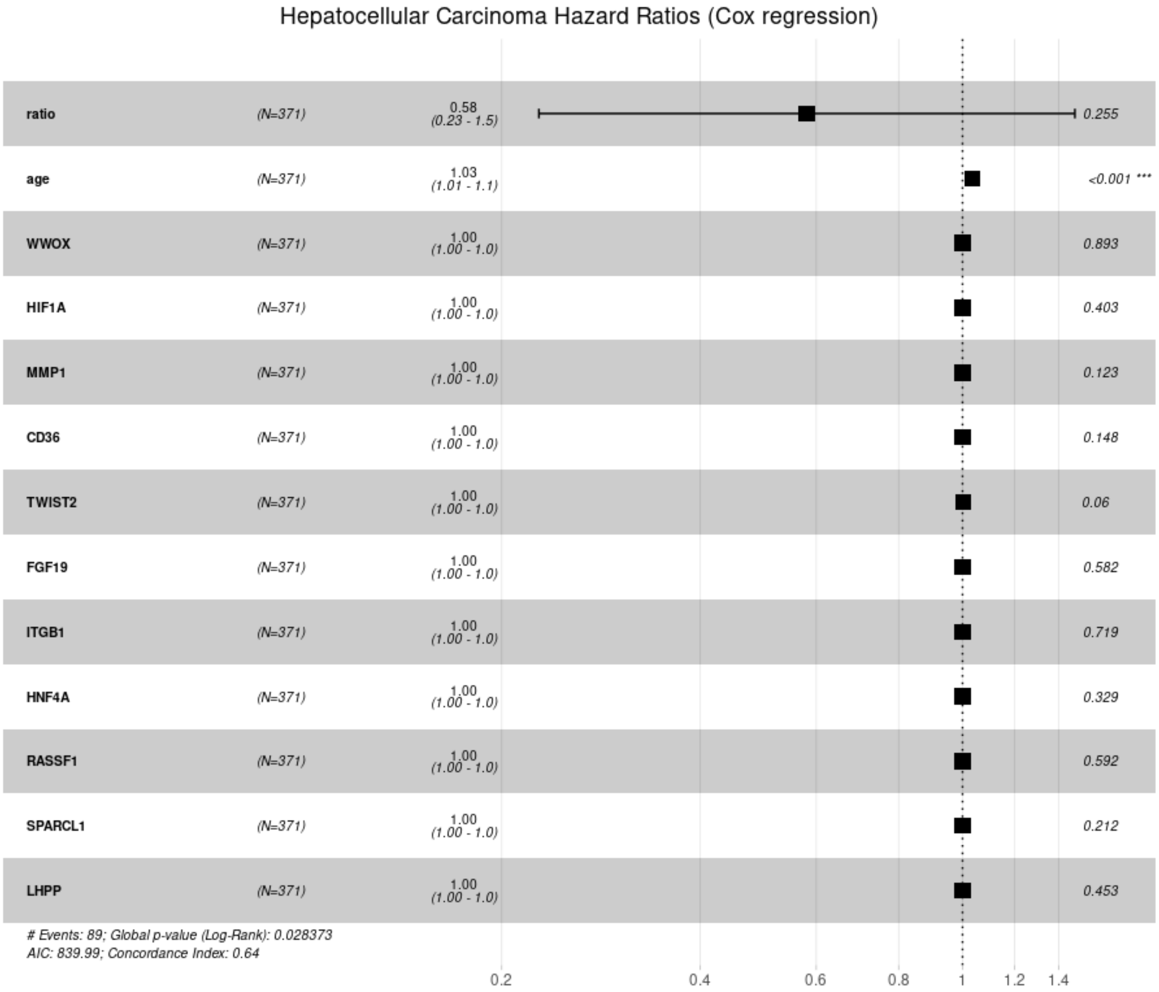

Supplementary Figure S5. Multivariate Cox Regression analysis of prognostic factors in hepatocellular carcinoma. Forest plot visualizing hazard ratios, confidence intervals, and significance for the WWOX/HIF1A ratio, gene expressions, age, and stage.

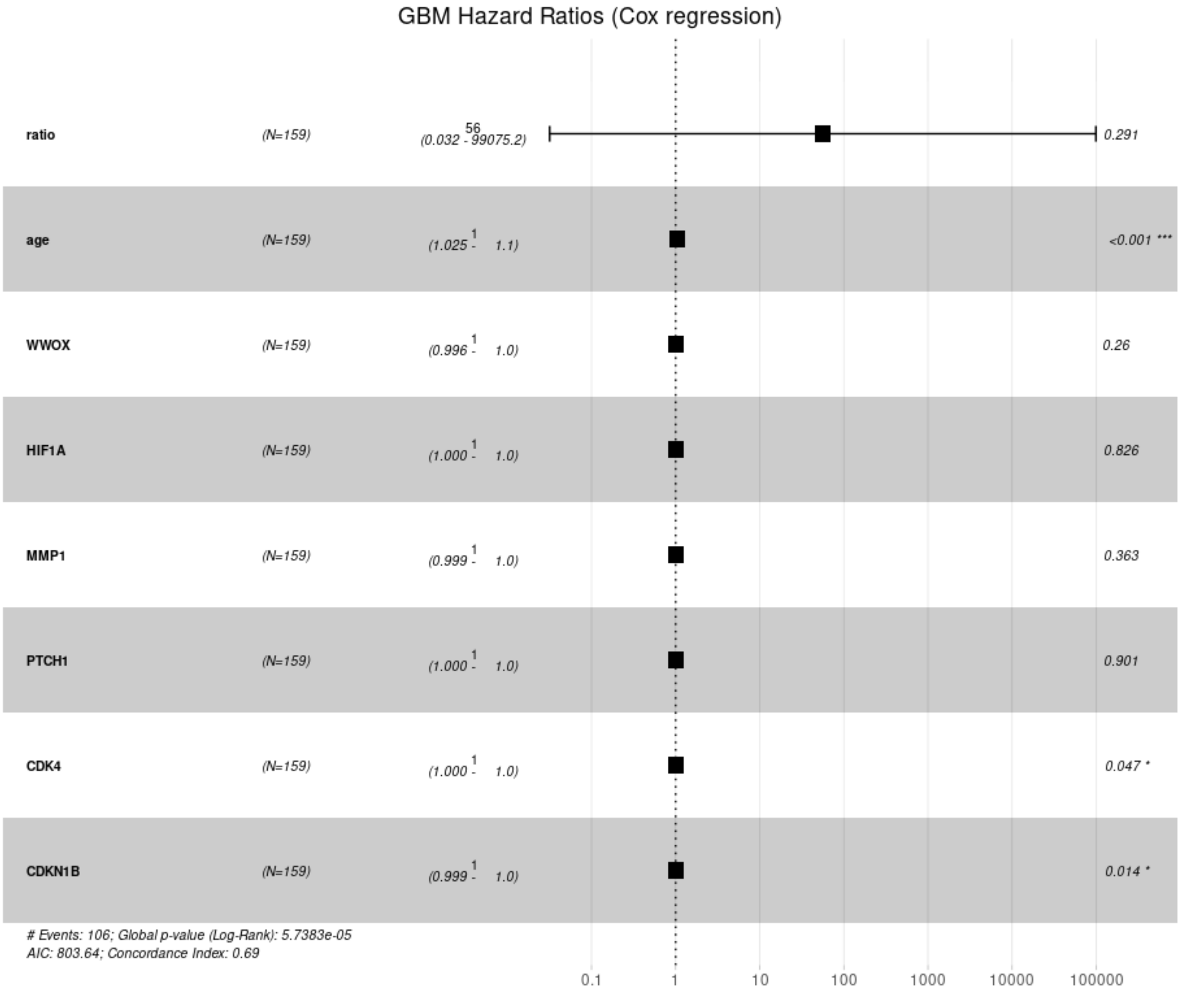

Supplementary Figure S6. Multivariate Cox Regression analysis of prognostic factors in glioblastoma. Forest plot visualizing hazard ratios, confidence intervals, and significance for the WWOX/HIF1A ratio, gene expressions, age, and stage.

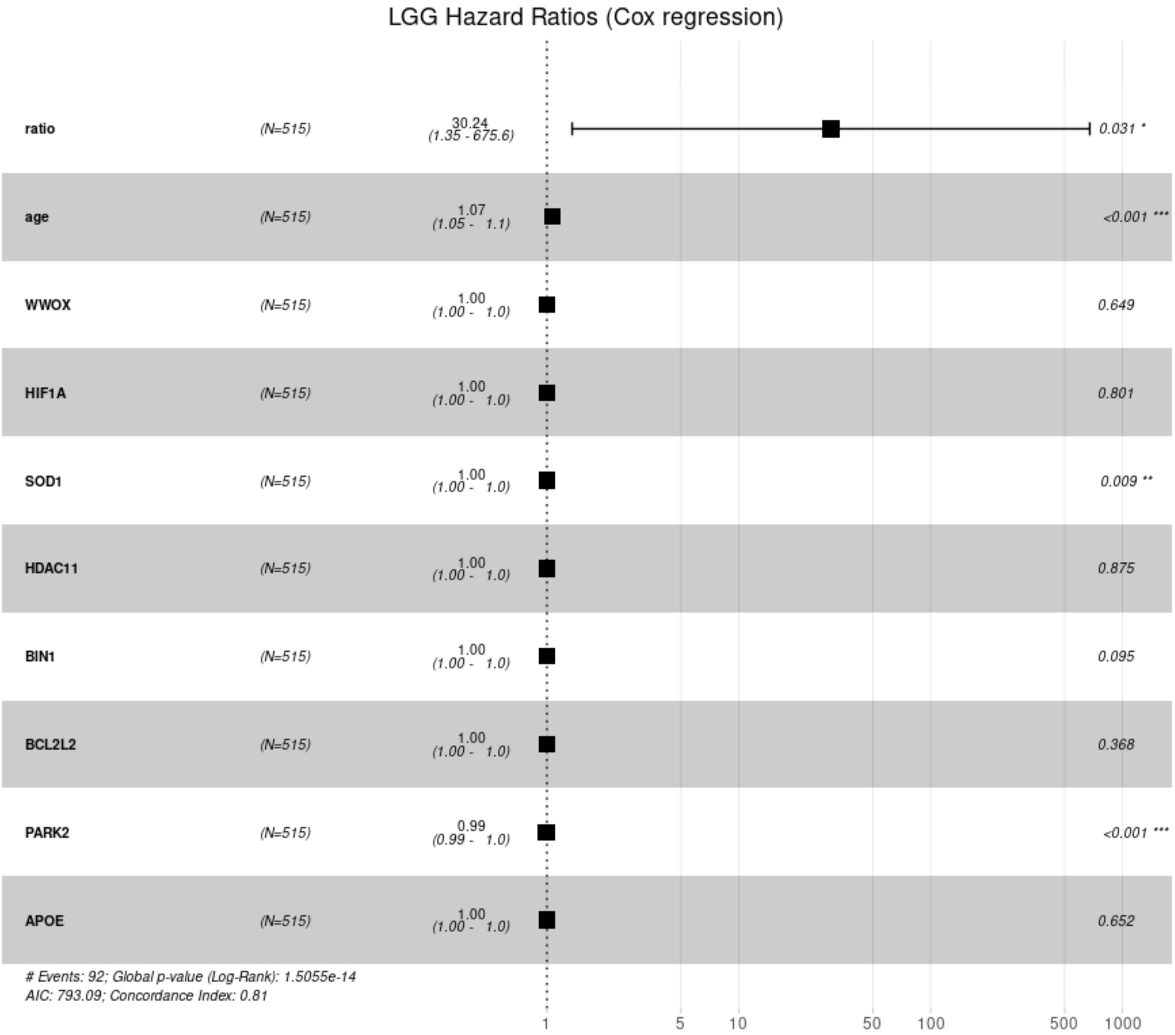

Supplementary Figure S7. Multivariate Cox Regression analysis of prognostic factors in low grade glioma. Forest plot visualizing hazard ratios, confidence intervals, and significance for the WWOX/HIF1A ratio, gene expressions, age, and stage.
